# Comparative Analysis of the Transcriptome across Distant Species

**DOI:** 10.1038/nature13424

**Published:** 2014-08-28

**Authors:** Mark B. Gerstein, Joel Rozowsky, Koon-Kiu Yan, Daifeng Wang, Chao Cheng, James B. Brown, Carrie A Davis, LaDeana Hillier, Cristina Sisu, Jingyi Jessica Li, Baikang Pei, Arif O. Harmanci, Michael O. Duff, Sarah Djebali, Roger P. Alexander, Burak H. Alver, Raymond Auerbach, Kimberly Bell, Peter J. Bickel, Max E. Boeck, Nathan P. Boley, Benjamin W. Booth, Lucy Cherbas, Peter Cherbas, Chao Di, Alex Dobin, Jorg Drenkow, Brent Ewing, Gang Fang, Megan Fastuca, Elise A. Feingold, Adam Frankish, Guanjun Gao, Peter J. Good, Roderic Guigó, Ann Hammonds, Jen Harrow, Roger A. Hoskins, Cédric Howald, Long Hu, Haiyan Huang, Tim J. P. Hubbard, Chau Huynh, Sonali Jha, Dionna Kasper, Masaomi Kato, Thomas C. Kaufman, Robert R. Kitchen, Erik Ladewig, Julien Lagarde, Eric Lai, Jing Leng, Zhi Lu, Michael MacCoss, Gemma May, Rebecca McWhirter, Gennifer Merrihew, David M. Miller, Ali Mortazavi, Rabi Murad, Brian Oliver, Sara Olson, Peter J. Park, Michael J. Pazin, Norbert Perrimon, Dmitri Pervouchine, Valerie Reinke, Alexandre Reymond, Garrett Robinson, Anastasia Samsonova, Gary I. Saunders, Felix Schlesinger, Anurag Sethi, Frank J. Slack, William C. Spencer, Marcus H. Stoiber, Pnina Strasbourger, Andrea Tanzer, Owen A. Thompson, Kenneth H. Wan, Guilin Wang, Huaien Wang, Kathie L. Watkins, Jiayu Wen, Kejia Wen, Chenghai Xue, Li Yang, Kevin Yip, Chris Zaleski, Yan Zhang, Henry Zheng, Steven E. Brenner, Brenton R. Graveley, Susan E. Celniker, Thomas R Gingeras, Robert Waterston

**Affiliations:** 1 Program in Computational Biology and Bioinformatics, Yale University, Bass 432, 266 Whitney Avenue, New Haven, Connecticut 06520, USA; 2 Department of Molecular Biophysics and Biochemistry, Yale University, Bass 432, 266 Whitney Avenue, New Haven, Connecticut 06520, USA; 3 Department of Computer Science, Yale University, 51 Prospect St, New Haven, Connecticut 06511, USA; 4 Department of Genetics, Geisel School of Medicine at Dartmouth, Hanover, New Hampshire 03755, USA; 5 Institute for Quantitative Biomedical Sciences, Norris Cotton Cancer Center, Geisel School of Medicine at Dartmouth, Lebanon, New Hampshire 03766, USA; 6 Department of Genome Dynamics, Lawrence Berkeley National Laboratory, Berkeley, California 94720, USA; 7 Department of Statistics, University of California, Berkeley, 367 Evans Hall, Berkeley, CA 94720-3860, USA; 8 Functional Genomics, Cold Spring Harbor Laboratory, Cold Spring Harbor, N, Y, 11724; 9 Department of Genome Sciences and University of Washington School of Medicine, William H. Foege Bldg. S350D, 1705 N.E. Pacific Street, Box 355065 Seattle, Washington 98195-5065, USA; 10 Department of Statistics, University of California, Los Angeles, CA 90095-1554, USA; 11 Department of Human Genetics, University of California, Los Angeles, CA 90095-7088, USA; 12 Department of Genetics and Developmental Biology, Institute for Systems Genomics, University of Connecticut Health Center, 400 Farmington Avenue, Farmington, CT 06030 USA; 13 Centre for Genomic Regulation, Barcelona, Catalonia, Spain; 14 Departament de Ciències Experimentals i de la Salut, Universitat Pompeu Fabra, Barcelona, Catalonia, Spain; 15 Center for Biomedical Informatics, Harvard Medical School, 10 Shattuck St. Boston, Massachusetts 02115, USA; 16 Department of Biostatistics, University of California, Berkeley, 367 Evans Hall, Berkeley, CA 94720-3860, USA; 17 Department of Biology, Indiana University, 1001 E. 3rd Street, Bloomington, Indiana 47405-7005, USA; 18 Center for Genomics and Bioinformatics, Indiana University, 1001 E. 3rd Street, Bloomington, Indiana 47405-7005, USA; 19 MOE Key Lab of Bioinformatics, School of Life Sciences, Tsinghua University, Beijing, China 100084; 20 National Human Genome Research Institute, National Institutes of Health, 5635 Fishers Lane, Bethesda, Maryland 20892-9307, USA; 21 Wellcome Trust Sanger Institute, Wellcome Trust Genome Campus, Hinxton, UK; 22 Center for Integrative Genomics, University of Lausanne, 1015 Lausanne, Switzerland; 23 Swiss Institute of Bioinformatics, 1015 Lausanne, Switzerland; 24 Medical and Molecular Genetics, King’s College London, London WC2R 2LS, UK; 25 Department of Genetics, Yale University School of Medicine, New Haven, Connecticut 06520-8005, USA; 26 Department of Molecular, Cellular and Developmental Biology, PO Box 208103, Yale University, New Haven, Connecticut 06520, USA; 27 Sloan-Kettering Institute, 1275 York Avenue, Box 252, New York, New York 10065, USA; 28 Department of Biological Sciences, Carnegie Mellon University, Pittsburgh, PA 15213 USA; 29 Department of Cell and Developmental Biology, Vanderbilt University, 465 21st Avenue South, Nashville, Tennessee 37232-8240, USA; 30 Developmental and Cell Biology, University of California, Irvine, CA 92697; 31 Center for Complex Biological Systems, University of California, Irvine, CA 92697; 32 Section of Developmental Genomics, Laboratory of Cellular and Developmental Biology, National Institute of Diabetes and Digestive and Kidney Diseases, National Institutes of Health, Bethesda MD 20892, USA; 33 Department of Genetics and Drosophila RNAi Screening Center, Harvard Medical School, 77 Avenue Louis Pasteur, Boston, Massachusetts 02115, USA; 34 Howard Hughes Medical Institute, Harvard Medical School, 77 Avenue Louis Pasteur, Boston, Massachusetts 02115, USA; 35 European Bioinformatics Institute, Wellcome Trust Genome Campus, Hinxton, CB10 1SD, UK; 36 Bioinformatics and Genomics Programme, Center for Genomic Regulation, Universitat Pompeu Fabra (CRG-UPF), Barcelona, Catalonia, Spain; 37 Institute for Theoretical Chemistry, Theoretical Biochemistry Group (TBI), University of Vienna; 38 Key Laboratory of Computational Biology, CAS-MPG Partner Institute for Computational Biology, Shanghai Institutes for Biological Sciences, Chinese Academy of Sciences, Shanghai 200031, China; 39 Hong Kong Bioinformatics Centre, The Chinese University of Hong Kong, Shatin, New Territories, Hong Kong; 40 5 CUHK-BGI Innovation Institute of Trans-omics, The Chinese University of Hong Kong, Shatin, New Territories, Hong Kong; 41 Department of Molecular and Cell Biology, University of California, Berkeley, California 94720, USA; 42 Department of Plant & Microbial Biology, University of California, Berkeley, California 94720, USA

The transcriptome is the readout of the genome. Identifying common features in it across distant species can reveal fundamental principles. To this end, the ENCODE and modENCODE consortia have generated large amounts of matched RNA-sequencing data for human, worm and fly. Uniform processing and comprehensive annotation of these data allow comparison across metazoan phyla, extending beyond earlier within-phylum transcriptome comparisons and revealing ancient, conserved features[1,2,3,4,5,6]. Specifically, we discovered co-expression modules shared across animals, many of which are enriched in developmental genes. Moreover, we used expression patterns to align the stages in worm and fly development, finding a novel pairing between worm embryo and fly pupae, in addition to the expected embryo-to-embryo and larvae-to-larvae pairings. Furthermore, we found that the extent of non-canonical, non-coding transcription is similar in each organism, per base-pair. Finally, we found in all three organisms the gene-expression levels, both coding and non-coding, can be quantitatively predicted from chromatin features at the promoter using a “universal model,” based on a single set of organism-independent parameters.

Our comparison used the ENCODE-modENCODE RNA resource (Fig. ED1). This resource comprises: (1) deeply sequenced RNA-Seq data from many distinct samples from all three organisms; (2) comprehensive annotation of transcribed elements and (3) uniformly processed, standardized analysis files, focusing on non-coding transcription and expression patterns. Where practical, these datasets match comparable samples across organisms and to other types of functional genomics data. In total, the resource contains 575 different experiments containing >67B sequence reads. It encompasses many different RNA types, including poly(A)+, poly(A)- and ribosomal-RNA-depleted RNA and short and long RNA.

The annotation in the resource represents capstones for the decade-long efforts in human, worm, and fly. The new annotation sets have numbers, sizes and families of protein-coding genes similar to previous compilations; however, the number of pseudogenes and annotated ncRNAs differ (Figs. ED2, ED3, S1). Also, the number of splicing events is greatly increased, resulting in a concomitant increase in protein complexity. We find the proportion of the different types of alternative splicing (e.g., exon skipping or intron retention) is generally similar across the three organisms; however, skipped exons predominate in human while retained introns are most common in worm and fly[7] (Figs. ED4, S1 and Table S1).

A fraction of the transcription comes from genomic regions not associated with standard annotations, representing “non-canonical transcription” (Table S2)[8]. Using a minimum-run/maximum-gap algorithm to process reads mapping outside of protein-coding transcripts, pseudogenes and annotated ncRNAs, we identified read clusters, i.e. transcriptionally active regions (TARs). Across all three genomes we found roughly one third of the bases gives rise to TARs, representing non-canonical transcription (Fig. ED3). To determine the extent that this transcription represents an expansion of the current established classes of ncRNAs, we identified the TARs most similar to known annotated ncRNAs using a supervised classifier[9] (Fig. S2, Table S2). We validated the classifier’s predictions using RT-PCR, demonstrating high accuracy. Overall, the predictions encompass only a small fraction of all TARs, suggesting that most TARs have features distinct from annotated ncRNAs and that the majority of ncRNAs of established classes have already been identified. To shed further light on the possible roles of TARs we intersected them with enhancers and HOT regions [8,10,11,12,13], finding statistically significant overlaps (Fig. ED5, Table S2).

Given the uniformly processed nature of the data and annotations, we were able to make comparisons across organisms. First, we built co-expression modules, extending earlier analysis[14](Fig. 1a). To detect modules consistently across the three species, we combined across-species orthology and within-species co-expression relationships. In the resulting multilayer network we searched for dense subgraphs (modules), using simulated annealing[15,16]. We found some modules dominated by a single species, whereas others contain genes from two or three. As expected, the modules with genes from multiple species are enriched in orthologs. Moreover, a phylogenetic analysis shows that the genes in such modules are more conserved across 56 diverse animal species (Figs. ED6, S3). To focus on the cross-species conserved functions, we restricted the clustering to orthologs, arriving at 16 conserved modules, which are enriched in a variety of functions, ranging from morphogenesis to chromatin remodeling (Fig. 1a, Table S3). Finally, we annotated many TARs based on correlating their expression profiles with these modules (Fig. ED5).

Next, we used the expression profiles of orthologous genes to align the developmental stages in worm and fly (Fig. 1b, ED7). For every developmental stage, we identified stage-associated genes, i.e. genes highly expressed at a particular stage but not across all stages. We then counted the number of orthologous pairs among these stage-associated genes for each possible worm-and-fly stage correspondence, aligning stages by the significance of the overlap. Strikingly, worm stages map to two sets of fly stages. First, they match in the expected one-to-one fashion to the fly (i.e. embryos-to-embryos, larvae-to-larvae). However, worm late embryonic stages also match fly pupal stages, suggesting a shared expression program between embryogenesis and metamorphosis. The ~50 stage-associated genes involved in this dual alignment are enriched in functions such as ion transport and cation-channel activity (Table S3).

To gain further insight into the stage alignment, we examined our conserved modules in terms of the “hourglass hypothesis”, which posits that all animals go through a particular stage in embryonic development (the tight point of the hourglass or “phylotypic” stage) during which the expression divergence across species for orthologous genes is smallest[4,5,17]. For genes in 12 of the 16 modules, we observed canonical hourglass behavior, i.e. *inter*-organism expression divergence across closely related fly species during development is minimal[5](Fig. S3). Moreover, we find a subset of TARs also exhibit this “hourglass” behavior (Fig. S2). Beyond looking at *inter*-species divergence, we also investigated the *intra*-species divergence within just *D. melanogaster* and *C. elegans*. Strikingly, we observed that divergence of gene expression between modules is minimized during the worm and fly phylotypic stages (Fig. 1c). This suggests, for an individual species, the expression patterns of different modules are most tightly coordinated (low divergence) during the phylotypic stage, but each module has its own signature before and after this. One can, in fact, directly see this coordination as a local maximum in between-module correlations for the worm (Fig. ED6). Finally, using genes from just the 12 “hourglass modules,” we found that the alignment between worm and fly stages becomes stronger (Fig. 1b, S3). The alignment shows a gap where no changes are observed, perfectly matching the phylotypic stage.

The uniformly processed and matched nature of the transcriptome data also facilitates integration with upstream factor-binding and chromatin-modification signals. We investigated the degree to which these upstream signals can quantitatively predict gene expression and how consistent this prediction is across organisms. Similar to previous reports[11,18,19], we found consistent correlations, around the TSS, in each of the three species between various histone-modification signals and the expression level of the downstream gene: H3K4me1, H3K4me2, H3K4me3 and H3K27ac are positively correlated, whereas H3K27me3 is negatively correlated (Figs. 2, ED8, S4). Then for each organism, we integrated these individual correlations into a multivariate, statistical model, obtaining high accuracy in predicting expression for protein-coding genes and ncRNAs. The promoter-associated marks, H3K4me2 and H3K4me3, consistently have the highest contribution to the model.

A similar statistical analysis with TFs showed the correlation between gene expression and transcription-factor (TF) binding to be the greatest at the TSS, positively for activators and negatively for repressors (Fig. ED8). Integrated TF models in each organism also achieved high accuracy for protein-coding genes and ncRNAs, with as few as five TFs necessary for accurate predictions (Fig. ED9). This, perhaps, reflects an intricate, correlated structure to regulation. The relative importance of the upstream regions is more peaked for the TF models than for the histone ones, likely reflecting the fact that histone modifications are spread over broader regions, including the gene body, whereas most TFs bind near the promoter.

Finally, we constructed a “universal model,” containing a single set of organism-independent parameters (Figs 2, S4). This achieved accuracy comparable to the organism-specific models. In the universal model, the consistently important promoter-associated marks such as H3K4me2 and H3K4me3 are weighted most highly. In contrast, the enhancer mark H3K4me1 is down-weighted, perhaps reflecting that signals for most human enhancers are not near the TSS. Using the same set of organism-independent parameters derived from training on protein-coding genes, the universal model can also accurately predict ncRNA expression.

Overall, our comparison of the transcriptomes of three phylo-genetically distant metazoans highlights fundamental features of transcription conserved across animal phyla. First, there are ancient co-expression modules across organisms, many of which are enriched for developmentally important “hourglass” genes. These conserved modules have highly coordinated intra-organism expression during the phylotypic stage, but display diversified expression before and after. The expression clustering also aligns developmental stages between worm and fly, revealing shared expression programs between embryogenesis and metamorphosis. Finally, we were able to build a single model that could predict transcription in all three organisms from upstream histone marks using a single set of parameters for both protein-coding genes and ncRNAs. Overall, our results underscore the importance of comparing divergent model organisms to human to highlight conserved biological principles (and dis-entangle them from lineage-specific adaptations).

## Methods

Detailed methods are in the supplement. (See first section of this for a guide.) Data sets described here can be obtained from the ENCODE project website at http://www.encodeproject.org via accession number ENCSR145VDW. More detail on data availability is in section F of the supplement.

## Extended Data

**Fig ED1:**
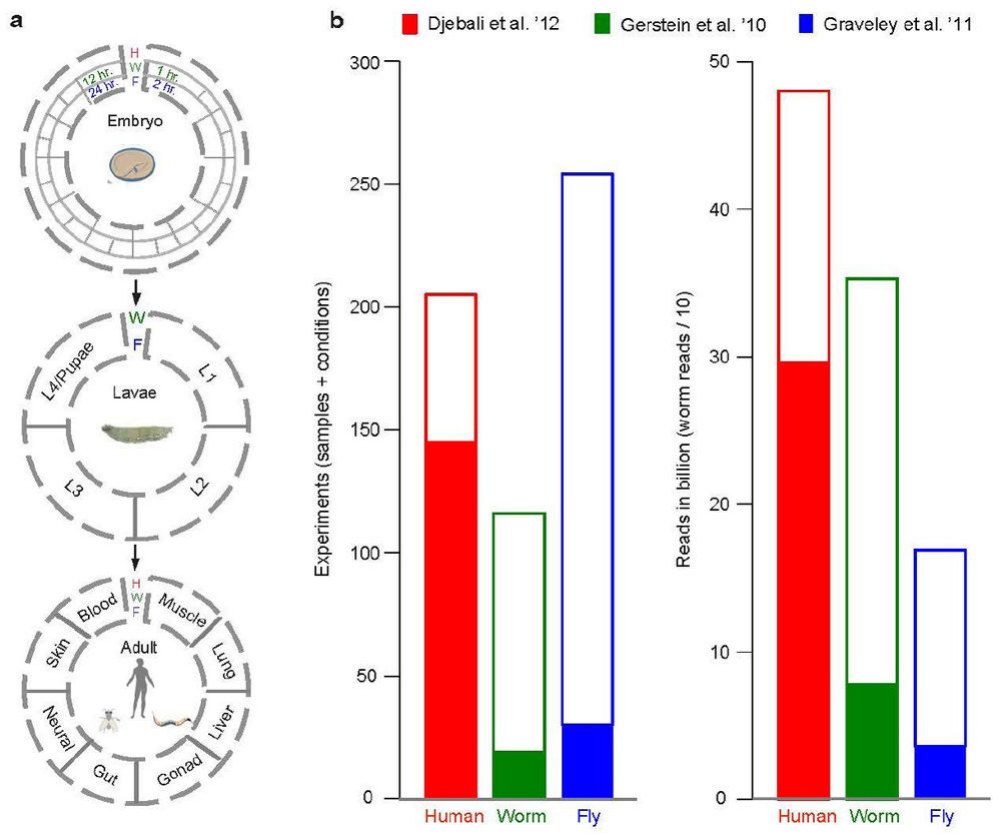
Overview of the data (A) Schematic of the RNA-seq data generated for human (red), worm (green), and fly (blue), showing how it samples developmental stages and various tissues and cell lines. (B) The number and size of data sets generated. The amount of new data beyond that in the previous ENCODE publications[8, 11, 20] is indicated by white bars, with previous ENCODE data indicated by solid bars. (See Supplement section B.2 for a detailed description of these data.)

**Fig ED2:**
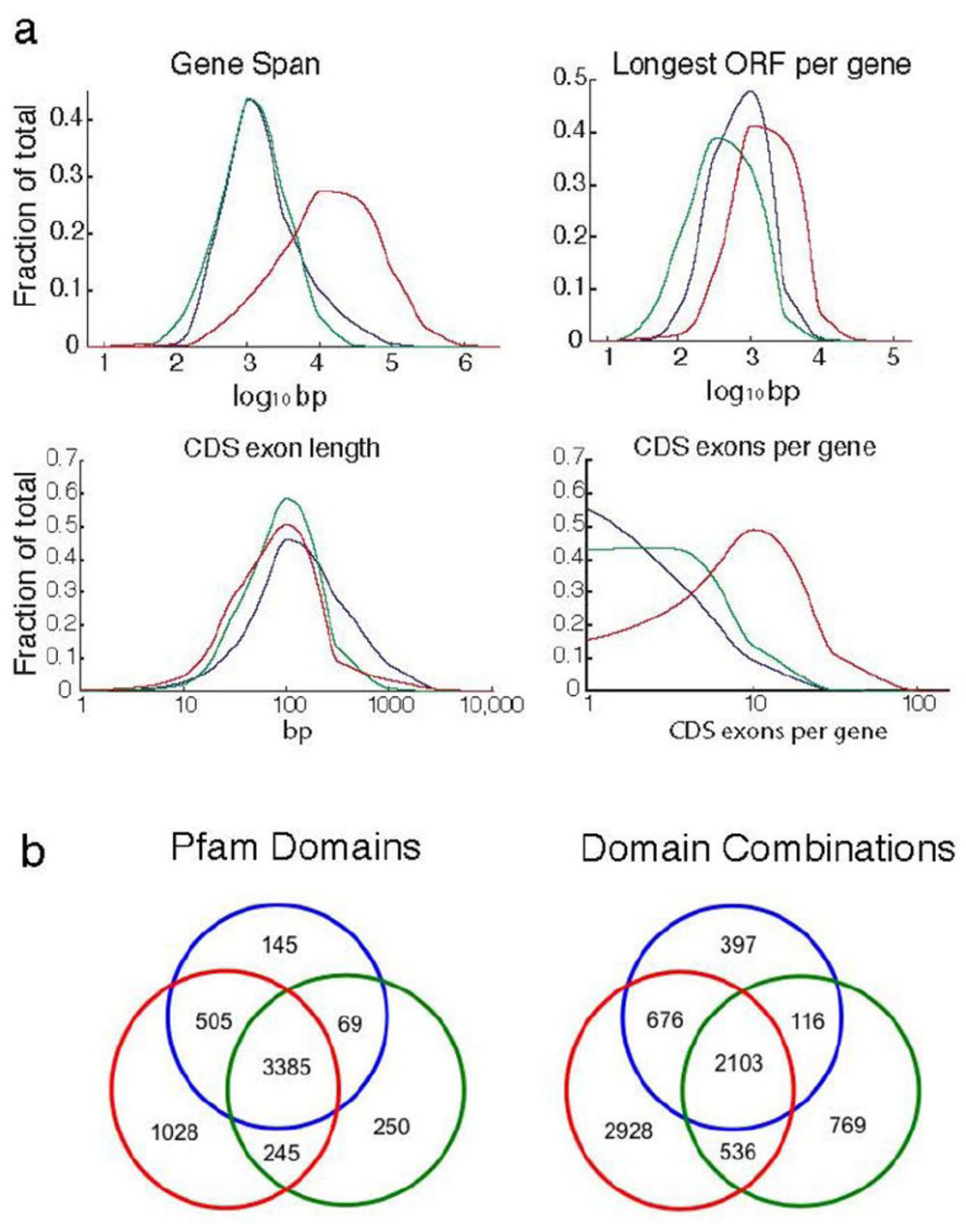
Summary plots for the protein-coding gene annotations (A) Distributions of key summary statistics - gene span, longest ORF per gene, CDS exon length, and CDS exons per gene; note that the x axes are in log scale. Both fly and worm genes span similar genomic lengths while human genes span larger regions (mostly due to the size of human introns). (B) Left: Venn diagram of protein domains (from the Pfam database version 26.0) present in annotated protein-coding genes in each species. Right: Shared domain combinations. (For more information on domain combinations, see Fig S1h and Supplement section B.4.1.)

**Fig ED3:**
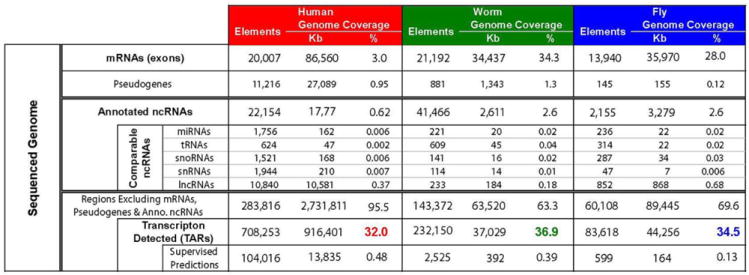
Summary of annotated ncRNAs, TARs, and ncRNA predictions in each species The number of elements, the base pairs covered and the fraction of the genome for each class (see also Supplement section C). There are comparable numbers of tRNAs in humans and worms but about half as many in fly. While the number of lncRNAs in human is more than an order of magnitude greater than in either worms or flies, the fractional genomic coverage in all three species is similar. Finally, humans have at least 5-fold more miRNAs, snoRNAs and snRNAs compared to worm or fly. The fraction of the genome covered by TARs (highlighted squares) for each species is similar. A large amount of non-canonical transcription occurs in the introns of annotated genes, presumably representing a mixture of unprocessed mRNAs and internally initiated transcripts. The remaining non-canonical transcription (249Mb, 16Mb, and 14Mb in human, worm, and fly) is intergenic and occurs at low levels, comparable to that observed for introns (Table S2). Overall, the fraction of the genome transcribed -- including intronic, exonic, and non-canonical transcription -- is consistent with that previously reported for human despite the methodological differences in the analysis (Fig. S2, Supplement section C).

**Fig ED4:**
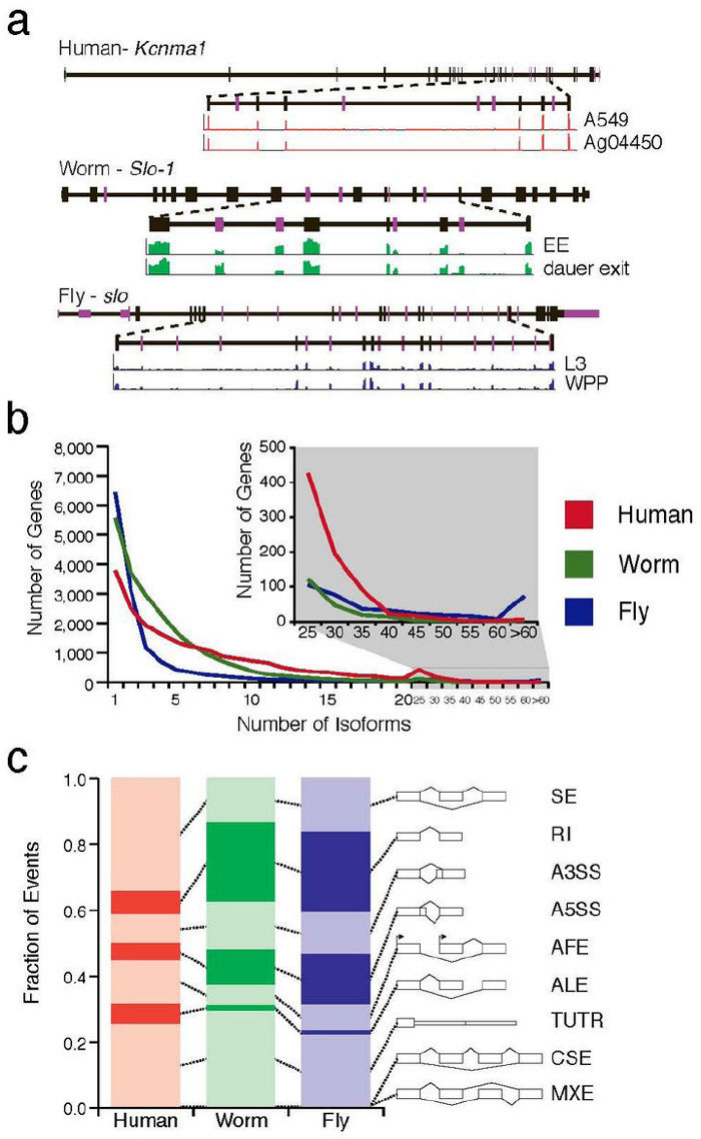
Analysis of Alternative Splicing (A) Representative orthologous genes do not share the same exon/intron structure or alternative splicing across species. (B) Distribution of the number of isoforms per gene. (C) Comparison of the fraction of various alternative splicing event classes in human, worm, and fly -- skipped exons “SE”, retained introns “RI”, alternative 3' splice sites “A3SS”, alternative 5' splice sites “A5SS”, alternative first exons “AFE”, alternative last exons “ALE”, tandem 3' UTRs “TandemUTR”, coordinately skipped exons “CSE”, and mutually exclusive exons “MXE”. (See Supplement section B.5 for a further discussion of splicing.)

**Fig ED5:**
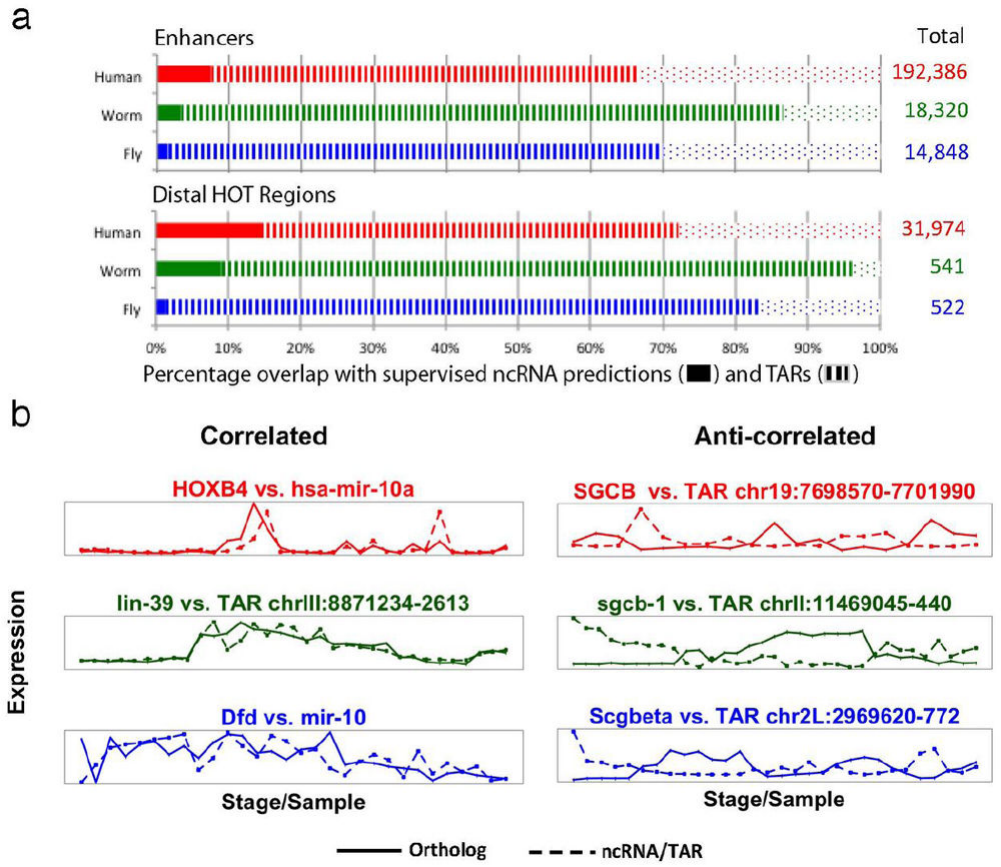
Characterizing Non-canonical Transcription (A) The overlap of enhancers and distal HOT regions with supervised ncRNA predictions and TARs in human, worm, and fly. The overlap of enhancers and distal HOT regions with respect to both supervised ncRNA predictions as well as TARs are significantly enriched compared to a randomized expectation. (B) The left side highlights ncRNA/TARs that are highly correlated with corresponding HOX orthologues in human (HOXB4), worm (lin-39), and fly (Dfd). The expression of mir-10 correlates strongly with Dfd in fly (r=0.66, p<6e-4 in fly), as does mir-10a in human, which correlates strongly with HOXB4 (r=0.88, p<2e-9). A TAR (chrIII:8871234-2613) strongly correlates with *lin-39* (r=0.91, p<4e-13) in worm. The right side shows TARs in human (chr19:7698570-7701990), worm (chrII:11469045-440), and fly (chr2L:2969620-772) that are negatively correlated with the expression of three orthologous genes: SGCB (r=-0.91, p<3e-16), sgcb-1 (r=-0.86, p<2e-7), and Scgb (r=-0.82, p<4e-8), respectively. (More details on all parts of this figure are in Supplement section C and Table S2.)

**Fig ED6:**
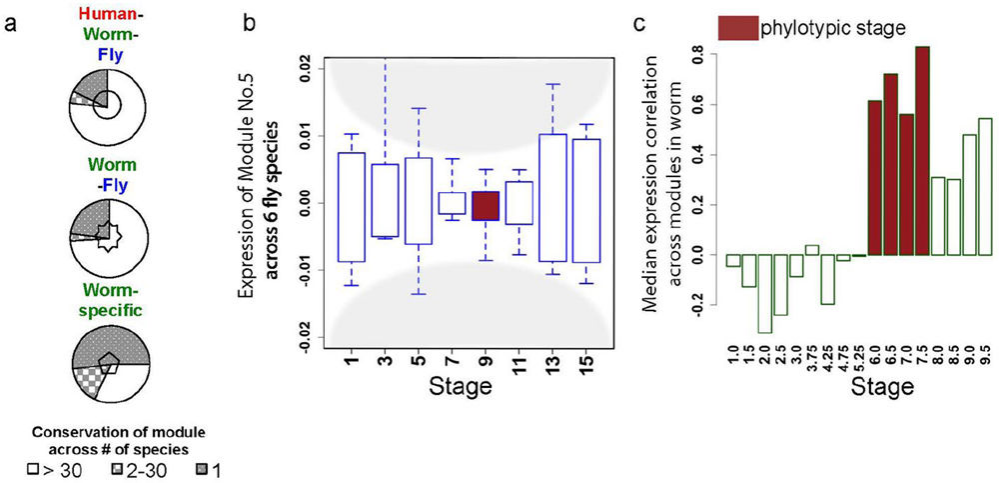
Details on Expression Clustering (A) Pie charts showing gene conservation across 56 Ensembl species for the blocks in the Fig. 1 heatmap enclosed with the same symbol (i.e. pentagon here matches pentagon in Fig.1a). Overall, species-specific modules tend to have fewer orthologs across 56 Ensembl species. (B) The expression levels of a conserved module (Module No. 5) in *D. melanogaster* and its orthologous counterparts in other 5 *Drosophila* species are plotted against time. The x-axis represents the middle time points of two-hour periods at fly embryo stages. The boxes represent the log10 modular expression levels from microarray data of 6 *Drosophila* species centered by their medians. The modular expression divergence (inter-quartile region) becomes minimal during the fly phylotypic stage (brown, 8-10 hours). (C) The modular expression correlations over a sliding 2-hour window (Pearson correlation per 5 stages, middle time of two-hour period in x-axis) among 16 modules in worm are plotted. The modular correlations (median shown as bar height in y-axis) are highest during the worm phylotypic stages (brown), 6-8 hours. One can, in fact, directly see this coordination as a local maximum in the between-module correlation for the worm, which has a more densely sampled developmental time course. (This figure provides more detail on Fig. 1a and 1c. More details on all parts of this figure are in Supplement section D and Figure S3.)

**Fig ED7:**
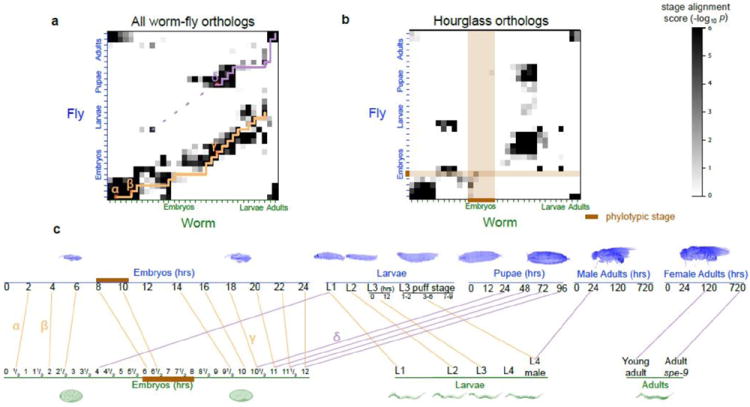
Details on Stage Alignment This figure provides further detail beyond Fig. 1b. (A) An alignment of worm and fly developmental stages based on all worm-fly orthologs (11,403 pairs, including one-to-one, one-to-many, many-to-many pairs). (B) Alignment of worm and fly developmental stages based on just worm-fly hourglass orthologs. Note the prominent gap in the aligned stages coincides with the worm and fly phylotypic stages (brown band). This make sense: since the expression values of genes in all hourglass modules converge at the phylotypic stage, no hourglass genes can be phylotypic-stage specific, and hence, the gap. (C) Key aligned stages from part (A). The correspondence between parts (A) and (C) is indicated by the small Greek letters. Worm “early embryo” and “late embryo” stages are matched with fly “early embryo” and “late embryo” respectively in the “lower diagonal” set of matches, and they are also matched with fly “L1” and “prepupa-pupa” stages respectively in the “upper diagonal” set of matches. (More details on all parts of this figure are in Supplement section D.4 and Table S3.)

**Fig ED8:**
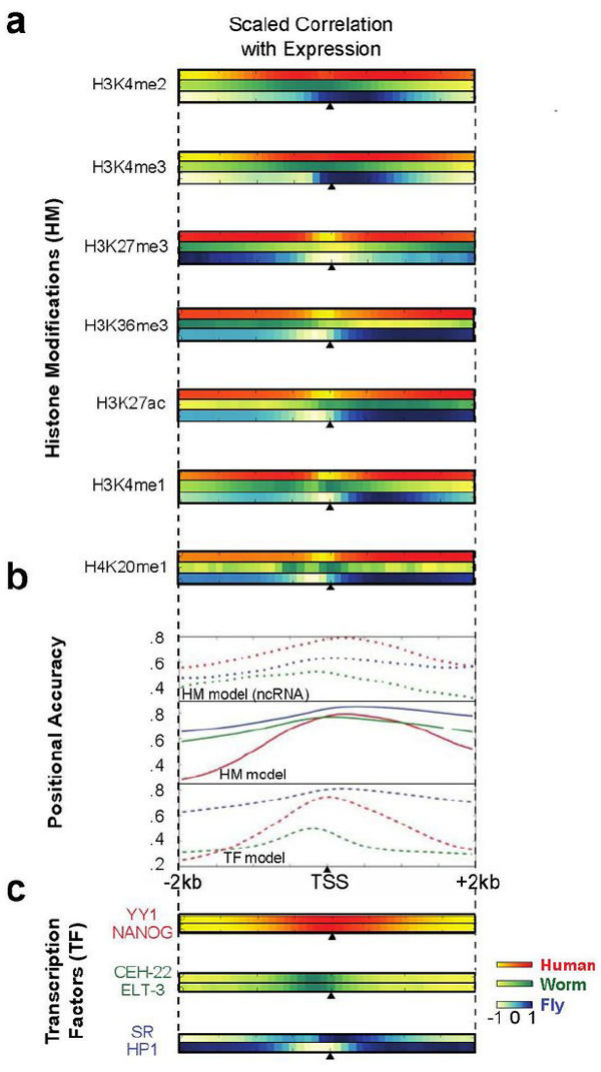
Further Detail on Statistical Models for Predicting Gene Expression This figure provides further information beyond that in Fig. 2. Binding/expression correlations of (A) various histone marks and (C) TFs. For instance, H3K36me3 shows positive correlation in worm and fly, but weak negative correlation in human at the promoter, with positive correlation over the gene body. (B) The positional accuracy from the TF and histone-mark models for predicting mRNA and ncRNA expression about the TSS. (More details on all parts of this figure are in Supplement section E and Fig. S4.)

**Fig ED9:**
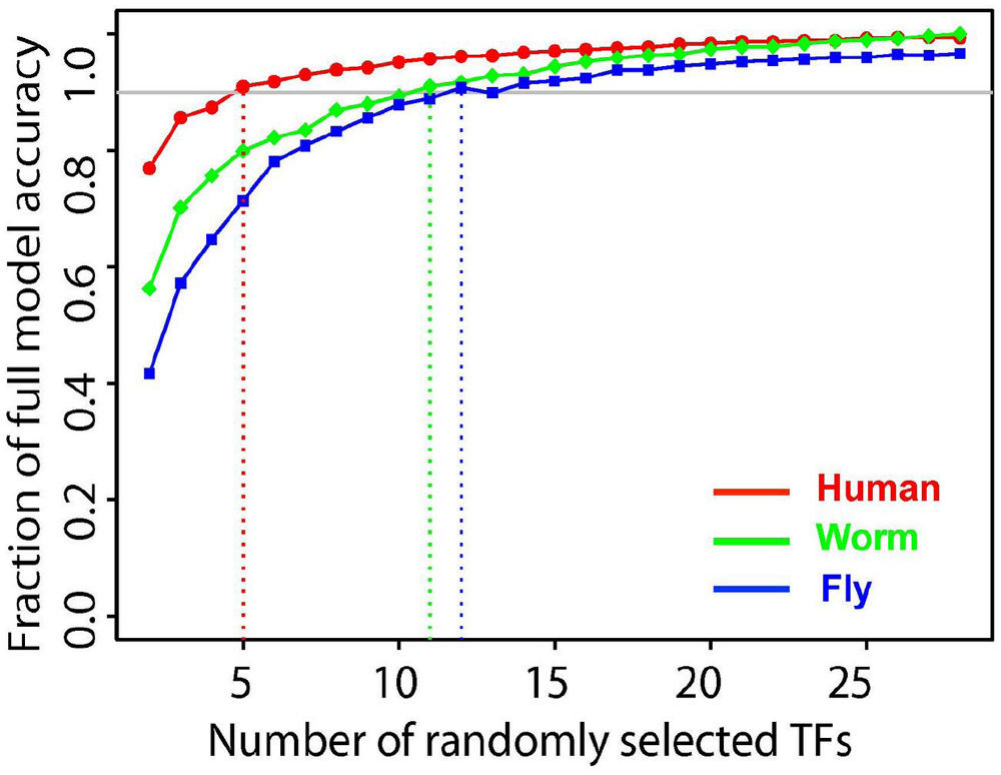
Average predictive accuracy of models with different number of randomly selected TFs We randomly selected n TFs as predictors and examined the predictive accuracy by cross-validation, where n varied from 2 to 28. The curve shows the average predictive accuracy (Fig. S4 indicates the standard deviation of all models with the same number of predictors). Surprisingly, models with as few as 5 TFs have predictive accuracy. This may reflect an intricate, correlated structure to regulation. However, it could also be that open chromatin is characteristic of gene expression and TFs bind somewhat indiscriminately. (More details on all parts of this figure are in Supplement section E.)

## Supplementary Material

1

## Figures and Tables

**Fig 1:**
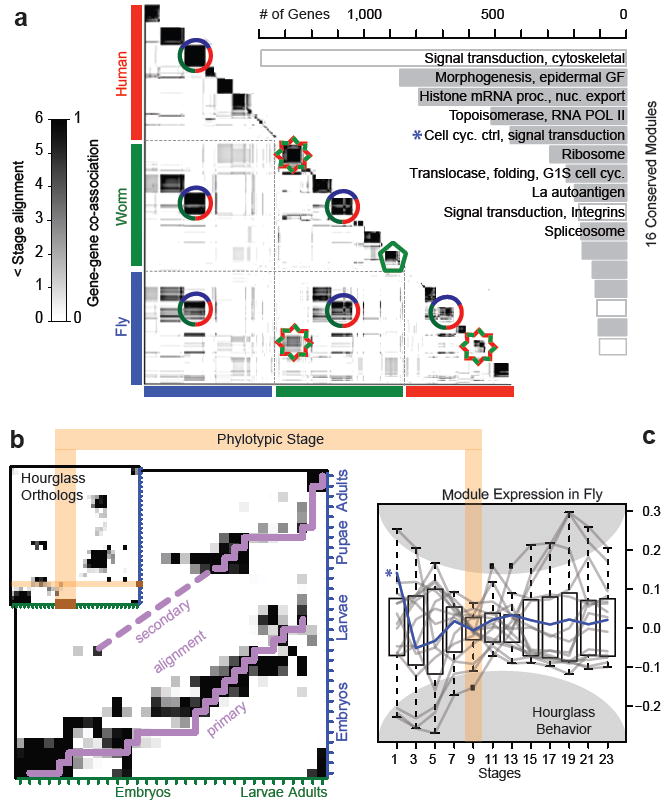
Expression Clustering (A) Left: Human, worm, and fly gene-gene co-association matrix; darker coloring reflects the increased likelihood that a pair of genes are assigned to the same module. A dark block along the diagonal represents a group of genes within a species. If this is associated with an off-diagonal block then it is a cross-species module (e.g. a three-species conserved module is shown with a circle and a worm-fly module, with a star). However, if a diagonal block has no off-diagonal associations, then it forms a species-specific module (e.g. green pentagon). Right: The GO functional enrichment of genes within the 16 conserved modules is shown. (B) Alignment of worm-and-fly developmental stages based on all worm-fly orthologs. Inset shows worm-fly stage alignment using only hourglass orthologs is more significant and exhibits a gap (brown) matching the phylotypic stage. (C) Normalized expression of the conserved modules in fly shows the smallest intra-organism divergence during the phylotypic stage (brown). (See Figs. ED 6 and 7 for further details.)

**Fig 2:**
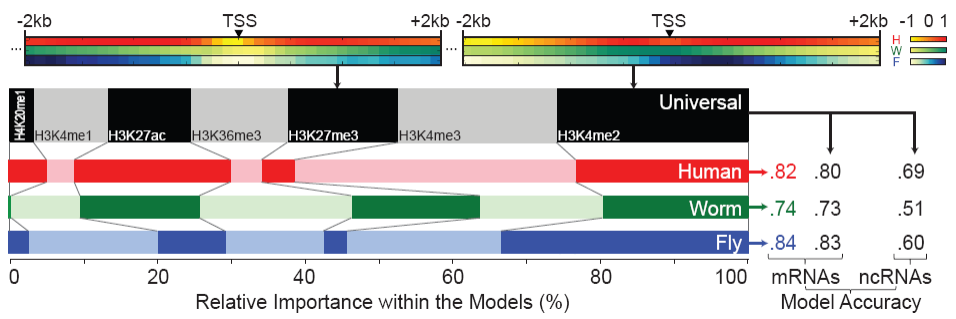
Histone Models for Gene Expression Top: Normalized correlations of two representative histone marks with expression. Left: Relative importance of the histone marks in organism-specific models and the universal model. Right: Prediction accuracies (Pearson correlations all significant, p<1e-100) of the organism-specific and universal models. (See Figs. ED 8 and 9 for further details.)
