# Supplementary material for: Comparative Analysis of the Transcriptome across Distant Species
Source: Nature. Author manuscript; Available in PMC 2015 Feb 28. (PMC4155737; doi:10.1038/nature13424)
Supplement: 1 [file NIHMS592121-supplement-1.pdf]

Comparative Analysis of the Transcriptome across Distant Species

Supplementary Information

A. Overview of the Supplement and Online Resources

The ENCODE and modENCODE consortia aim to identify all gene features and build the encyclopedia of DNA elements for the three distant metazoans - human, worm and fly. In this paper, we made use of all data from the consortia for an integrative comparison of transcriptomes in these three species. Through uniform analysis, we were able to identify comparable elements among different species, as well as distinguishable features uniquely belonging to a specific species. More details of the data sets and the uniform analysis are presented here in this Supplementary Information. The supplementary figures present information not included in the main figures or extended data (ED) figures. They also include several "shadow figures" of those, which are similarly drawn but contain more detailed information.

Note that the flow of main text is divided into 4 parts, in the order: Comparative ENCODE RNA Resource, ncRNAs & Non-Canonical Transcription, Expression Clustering and Stage Alignment, Modeling Gene Expression with Chromatin & TFs. This supplement is laid out in a parallel fashion. Sections B, C, D, E are devoted to the 4 parts respectively, providing more details and background context to the major results presented in the main text. Also, for each of these sections there are major supplement figures and tables, which, in turn, have further of sub-parts (i.e. Figures S1 to S4 and Tables S1 to S3, which have subparts such as Figure S2g). Finally, the supplement is connected to the extended-data figures, in that the caption of each of these refers to specific supplement sections, figures and tables as providing more detail.

We made comprehensive analyses of a large amount of genomic data across three species. This paper, including this supplement, can be viewed as a hierarchical information structure, designed to present both data and results in an organized fashion. In the hierarchy, the "main" text sits at the top, synthesizing everything broadly. It then points to more detailed descriptions of our methods and data, which were divided into sections A-G. Sections B-E each addresses one coherent aspect of the analyses, with the order of appearance concordant with the main text. Each of these individual sections often refers to a huge amount of supplementary calculations and data sets. Moreover, the data sets to which the paper refers the most are usually not the actual raw data per se, but subsidiary analysis products that summarize the data (e.g., RPKM and modules). Some of the data sets are presented in formal paper supplements, while others

are on our paper website (Section F). At the bottom of the hierarchy, section G, is the actual underlying raw data (usually RNA-Seq reads), stored in central repositories (such as the short read archive (SRA)). Raw data files are usually huge and unwieldy. Consequently, it makes most sense to approach the information in a particular freeze from the top down.

Supplementary Information 1
A. Overview of the Supplement and Online Resources 1
B. More Details for “Comparative ENCODE RNA Resource” 5
B.1. Context for “Comparative ENCODE RNA Resource” 5
B.2. RNA-Seq Data Generation 6
B.2.1. ENCODE Human RNA-Seq Data Generation 6
B.2.2. Worm RNA-Seq Data Generation 6
B.2.3. Fly RNA-Seq Data Generation 7
B.3. Annotation 7
B.3.1. Human-Worm-Fly Orthologs 8
B.3.2. Pseudogene Annotation 8
B.3.3. ncRNA Annotation 8
B.4. Comparison of Protein-Coding Genes 12
B.4.1. Domain Combinations 12
B.4.2. Broadly Expressed Protein-Coding Genes 13
B.5. Splicing 14
B.5.1. Comparison of Splicing In Annotation Sets 15
B.5.2. Splicing Quantification Alignment Pipeline 16
C. More Details on “ncRNAs & Non-Canonical Transcription” 20
C.1. Context for “ncRNAs & Non-Canonical Transcription” 20
C.2. Consistent ncRNA Pipeline Processing 21
C.2.1. TAR Calling 21
C.2.2. Calibrating PolyA+ RNA 22
C.3. Consistency with Previous ENCODE Estimates 23
C.3.1. Consistency of ROC Analysis and IDR Thresholding 23
C.3.2. Increase in Coverage Using Total RNA and Compartments 23
C.4. Supervised ncRNA Predictions 24
C.4.1. The Gold Standard ncRNA Data Sets 24
C.4.2. Data Sets and Predicted ncRNA Filter, Annotation and Validation 24
C.5. Relationship of HOT Regions and Enhancers to Non-coding Transcription 25
C.6. Mapping ncRNAs and TARs to Modules 26
D. More Details on “Expression Clustering & Stage Alignment” 26
D.1. Context for “Expression Clustering & Stage Alignment” 26
D.1.1. Expression clustering 27
D.1.2. Stage alignment 27
D.1.3. Analysis of the phylotypic stage 27
D.2. Constructing Co-expression Modules 28

D.2.1. Transforming Expression Profiles into Co-Expression Networks........ 28
D.2.2. The Potts Model........ 28
D.2.3. Simulated Annealing and Defining Confident Modules........ 29
D.2.4. Further Clustering of the Conserved Modules........ 30
D.3. Identification of Hourglass Behavior........ 30
D.3.1 Methods: Quantifying Modular Expression........ 30
D.3.2 Results........ 31
D.3.3 Significance & Discussion........ 31
D.4. Stage Alignment during Developmental Time-Course........ 32
E. More Details on "Modeling Gene Expression with Chromatin & TFs"........ 32
E.1. Context for "Modeling Gene Expression with Chromatin & TFs"........ 32
E.2. Relating Pol II Binding and H3K4me3 with Gene Expression........ 33
E.3. Predictive Models for Gene Expression........ 33
E.3.1. Data Preprocessing........ 33
E.3.2. Details on the Models........ 34
E.3.3. An Organism-Independent Universal Model........ 35
F. Associated Data Files........ 36
F.1. Associated Data Files for "Comparative ENCODE RNA Resource"........ 36
F.1.1. RNA-Seq Data Sets........ 36
F.1.2. Protein-Coding Gene Annotation........ 36
F.1.3. Fly Strict Non-coding Gene........ 36
F.1.4. Comparable and Non-comparable Non-coding RNA Annotations........ 36
F.1.5. Human-Worm-Fly Ortholog Lists........ 37
F.1.6. Table Summarizing All Annotations and Processed Values for Every Gene........ 37
F.2. Associated Data Files for "ncRNAs & Non-Canonical Transcription"........ 37
F.2.1. TARs........ 37
F.2.2 Clustering of ncRNAs and TARs with Modules........ 38
F.2.3. Supervised ncRNA predictions (novel ncRNA fragments)........ 38
F.3. Associated Data Files for "Expression Clustering & Stage Alignment"........ 38
F.3.1. Gene Co-expression Modules........ 38
F.4. Associated Data Files for "Modeling Gene Expression with Chromatin & TFs"........ 39
G. Online data........ 39
G.1. modENCODE.org & encodeproject.org........ 39
G.2. WormBase, FlyBase, SRA and Beyond........ 39
References........ 41
Supplementary Tables........ 57
Table S1 - Tables Giving More Detail on "Comparative ENCODE RNA Resource"........ 57
Table S1a - Tables of human RNA-Seq data........ 57
Table S1b - Tables of broadly expressed protein-coding genes........ 62
Table S1c - Tables of splicing annotation........ 64
Table S2 - Tables Giving More Details on "ncRNAs & Non-Canonical Transcription"........ 66
Table S2a (Shadow for ED Fig. 3) - Table of annotated ncRNAs in human, worm, and fly........ 66
Table S2c - Gold standard ncRNA numbers for supervised ncRNA predictions........ 70
Table S2d - Numbers of predicted ncRNA candidates in three organisms, sensitivity cut off 0.95........ 71

Table S2f - Supervised ncRNA predictions training on whole genome compared with previous predictions training on conserved regions of C.elegans. 73
Table S2g - Annotation source. 74
Table S2h - Intersection of enhancers and distal HOT regions with TARs and incRNAs 74
Table S3 - Tables Giving More Details on "Expression Clustering & Stage Alignment" 75
Table S3a - Statistics related to each of the conserved modules. 75
Table S3b - Early embryo specific worm genes that are aligned to both fly genes in specific in embryos (18-20hr) and pupae. 76
Supplementary Figures 84
Fig. S1 - Figures Giving More Details on the "Comparative ENCODE RNA Resource" 84
Fig. S1a - Evolution of protein-coding gene annotation in human (red), worm (green), and fly (blue). 84
Fig. S1b - Gene expression in HeLa-S3 cells. 85
Fig. S1c - Broadly expression scores 86
Fig. S1d - Maximum expression vs. variability score and distribution of variability score. 87
Fig. S1e - Normalized expression levels of broadly and specifically expressed protein-coding genes. 88
Fig. S1f - Comparison of alternative splicing 89
Fig. S1g - Distributions of splicing changes. 90
Fig. S1h - Venn diagrams of protein domains. 91
Fig. S2 – Figures Giving More Details on "ncRNAs & Non-canonical Transcription". 92
Fig. S2a - Comparison of poly(A)+, total RNA and short RNA for detecting ncRNAs. 92
Fig. S2b - ncRNAs and non-canonical transcription. 96
Fig. S2c - Expression distributions of the TARs and exons in all three species. 97
Fig. S2d - Supervised ncRNA predictions 98
Fig. S2e - Further example of highly correlated TAR triplet with 1-1-1 orthologous genes. 102
Fig. S3 - Figures Giving More Details on "Expression Clustering & Stage Alignment". 104
Fig. S3a - Clustering procedure. 104
Fig. S3b – Schematic illustration of Potts modules conserved across three species but with different gene duplication rate 105
Fig. S3c - Clustering of orthologs in 3 species. 106
Fig. S3d - Hourglass patterns of 16 conserved modules. 107
Fig. S3e – Hourglass pattern of fly gene expressions, and schematic visualization of worm modular expressions 108
Fig. S3f - Worm-Fly Developmental Stage Alignment 110
Fig. S4 – Figures Giving More Details on "Modeling Gene Expression with Chromatin and TFs". 112
Fig. S4a - H3K4me3 vs. expression. 112
Fig. S4b - More Details on the Predictive Model for Gene Expression. 114

B. More Details for "Comparative ENCODE RNA Resource"

B.1. Context for "Comparative ENCODE RNA Resource"

The data used to prepare this manuscript includes the last ENCODE and modENCODE rollout [1, 2, 3], together with new data specifically generated for the transcriptome comparison between human, worm and fly (ED Fig. 1). This data compendium covers comprehensive sampling conditions in the three organisms and provides high transcriptome coverage. In total, we have done 575 experiments and produced more than 65 billion reads from the three organisms, where a majority of new data are from Illumina GAIIx or HiSeq2000 paired-end reads of length 100 base pairs each. The total amount of new data is ~380 experiments and ~34 billion reads. The scale of the data makes it possible to create a complete transcriptome annotation including coding transcripts and ncRNAs for each organism. Furthermore, the data has been generated with the focus of matching conditions as much as possible across all three organisms, such as matching life stages or matching tissue types. These matched data sets are necessary for comparing the highly diverged transcriptomes from human, worm and fly and for finding out the preserved transcription principles across all three organisms. The transcriptome data compendium has also been integrated with histone modification data and transcription factor binding data for a more comprehensive interpretation.

Some comparative transcriptome studies have been reported before. However, the comparisons have been limited to within closely related organisms (i.e., just mammals or yeast) [4, 5, 6, 7, 8, 9, 10, 11, 12, 13] or between worm and fly [14], hence the promise of the model organisms for providing insight into preserved principles applicable to human genome annotation has yet been studied. In addition, we generated a data set with much higher transcriptome coverage than any previous studies. Whereas each previous study has generated no more than 16 billions reads with read length less than or equal to 76 nucleotides, we created a data set of more than 68 billions read with longer read length (76 or 100 bp long). It would not be possible to carry out the comparison described here with only 25% of the reads that we used. Such high trancriptome coverage allows us to compare the transcriptions of annotated and novel ncRNAs across the three organisms, and to carry out ortholog clustering and stage mapping analyses, as detailed in the manuscript. Compared to the full spectrum of transcriptome covered in current study, most previous studies only focused on comparison of protein coding genes [4, 6, 7], or some specific set of ncRNAs such as miRNAs [5]. Cross organism comparison based on microarrays is much more problematic given probe bias and normalization issues [14].

Compared to the previous modENCODE rollout data [2, 3, 15], the numbers of tissues and conditions included in the current worm and fly data sets have been increased by about an order of magnitude. In particular, the experimental conditions have been increased from 19 to 116 for worm, and from 30 to 254 for fly. The current data sets cover much broader range of tissues and more accurately defined life

stages. The total RNA-Seq reads in both organisms have also been greatly increased (3.5 fold for worm and 4 fold for fly). Furthermore, two new types of fly data have been included since the previous rollout. First, 40 CAGE data sets have been newly added. CAGE data measures the expression levels at transcription start sites (TSS), and hence greatly improves the TSS annotation. This improvement is essential for accurate integration with the histone modifications and TFs and can be seen in Fig. 2. Second, the new fly data includes 116 tissue data sets, which are necessary to match the human and worm tissues for the comparative studies. The scale of human data has also had a notable increase (from 17 cell lines to 41 cell lines, and from 145 libraries to 205 libraries, Table S1), though less significant than those of worm and fly due to the relatively short time (about one year) since the previous human ENCODE rollout [16]. However, it worth to mention that 23 new primary cell lines have been added to the current human data set, in addition to the 8 primary cell lines in the previous rollout. Primary cell lines are capable of providing more wild type profiles of transcriptome than immortalized cell lines due to their more normal karyotype. The entire data compendium encompasses many RNA types, including poly(A)+, poly(A)- and total RNA; both short (<50 nt) and long RNA (>200 nt); and CAGE data.

Many of the analyses included in this paper focus primarily on the long poly(A)+ RNA-Seq prepared from whole-cells which are most comparable across the three organisms when used collectively. There are some analyses (such as the supervised ncRNA predictions) that make use of all the available RNA-Seq data. The sample preparation methods in each organism and the scope of the data generated are detailed as follow. A complete list of the data sets can be seen in Associated Data Files, where the accession numbers of all the data are also included.

B.2. RNA-Seq Data Generation

B.2.1. ENCODE Human RNA-Seq Data Generation

We performed subcellular compartment fractionation (whole cell, nucleus and cytosol) before RNA isolation to interrogate the human transcriptome. The RNAs >200 nucleotides from each of these subcompartments were prepared in replica. Long RNAs were further fractionated into polyadenylated and non-polyadenylated transcripts. The RNAs were depleted of rRNAs and libraries were prepared using the dUTP protocol [17]. Libraries were sequenced in mate-pair fashion on the Illumina GAIIx or Hi-Seq to an average depth of 100 million mate pairs per replicate. Additional information related to sample preparation can be obtained in the reference [16]. Detailed protocol information is also available by downloading the Production Documents for libraries of interest from GEO. In total 205 RNA-Seq libraries were constructed from 42 cell lines with different subcellular compartments and RNA populations. For details of data sets, see Associated Data Files.

B.2.2. Worm RNA-Seq Data Generation

We have used RNA-Seq to capture information about the transcribed genome of C. elegans. To increase the detection of stage/condition specific transcripts we have assayed animals in biological replicates from all the major stages of the life cycle (early embryo, late embryo, L1, L2, L3, L4, young adult, old adult, dauer entry, dauer, dauer exit, male enriched embryo, L4 male and adults lacking germline). In

addition, we developed improved embryo synchronization methods and assayed embryonic development at higher resolution (25 samples every 30 minutes across embryonic development). To look for genes that might be involved in the response to pathogens, we assayed adults exposed to a variety of bacteria for different lengths of time. These libraries were constructed from whole organisms and isolated tissues using poly(A) enriched RNA populations and were not oriented with respect to the strand of the transcript (although that could be inferred for most transcripts from the inherent strandedness of features such as splice junctions, splice leaders and poly(A) tails). Total RNA-Seq libraries were also constructed from various tissues (muscle, neuron, skin, pharyngeal gland, embryonic seam cells, sheath cells and epithelial cells) and life stages (early embryo, late embryo, L2, L4 and young adult). In total 106 RNA-Seq libraries were constructed. For details of data sets, see Associated Data Files.

B.2.3. Fly RNA-Seq Data Generation

Dissected organ systems were extracted from Oregon R larval, pupal and adult staged animals synchronized with appropriate age indicators. To detect RNAs not observed under wild-type conditions we used environmental perturbations to challenge a population of mixed adult males and females with temperature variation, heavy metals, drugs and pesticides. Larvae were treated with heavy metals, drugs and the pesticide rotenone. In addition to the previously described cell lines [18] we have sequenced three adult ovary-derived lines OSS, OSC and fGS. RNA was isolated using TRIzol (Invitrogen), DNased, and purified on a RNAeasy column (Qiagen). poly(A)+ RNA was prepared from an aliquot of each total RNA sample using an Oligotex kit (Qiagen). For complete details see Brown et al., (2013) [19].

Strand-specific libraries were generated and sequenced on an Illumina HiSeq2000 using paired-end chemistry and 100 bp cycles. Sequencing was done for poly(A)+ RNA and biological duplicates were used. In total 354 RNA-Seq libraries were constructed. For details of data sets, see Associated Data Files.

B.3. Annotation

Evolution of protein-coding gene annotation in human, worm and fly is summarized in Fig. S1. The length of a gene is defined as the number of nucleotides from the start of the message to the poly(A) site. Based on the newly generated datasets, we developed annotation sets for worm and fly extending beyond WormBase WS220 and FlyBase 5.45.

The annotation of protein coding genes is fairly stable, whereas the annotation of non-coding elements is more fluid. In particular, with each iteration of the annotation, the overall number of protein coding genes has not substantially changed. For instance, for the human, the number of protein coding genes was 19,599 in the first version of GENCODE and five years later is now 20,007 (Fig. S1). In contrast, the numbers of non-coding loci in the corresponding gene-sets have increased considerably. Given this fact, we have taken a two-pronged approach to compare across organisms. For protein coding genes, we have used the current annotations and compared them without modification. However, for non-coding and pseudogene loci, we have uniformly reprocessed and harmonized the annotations to make them more comparable between organisms. Finally, to facilitate direct comparisons between the three species, we utilized sets of protein-coding gene orthologs with varying stringency in our analyses [20]. These

include triplets of 1-to-1-to-1 orthologs as well as one-to-many and many-to-many orthology relationships.

B.3.1. Human-Worm-Fly Orthologs

We have compiled a complete list of ~28k triplets of orthologous genes among human, worm and fly (6353 unique genes in human, 5083 unique genes in worm, 4839 unique genes in fly) for the purpose of this paper. The list was merged from the MIT list [20] and Ensembl. It contains all one-to-one, one-to-many and many-to-many orthologous relationships. In particular, there is a subset of ~2000 triplets of orthologous genes among human, worm and fly which includes one-to-one orthologous relationships only. The list of orthologs can be found in the Associated Data Files.

B.3.2. Pseudogene Annotation

Often referred to as "genomic fossils" [21, 22], pseudogenes are disabled copies of a parent protein-coding gene. Overall, the pseudogenes differ greatly between the three organisms. The human genome has 11216 pseudogenes, which are about 12-fold more than worm (881), and about 100-fold more than fly (145). Ratios of the pseudogenes between the three genomes do not match their relative genome sizes or gene counts.

The pseudogene annotation has been conducted using a combination of manual annotation and in silico pipelines: RetroFinder (human) and PseudoPipe (human, worm, and fly). Pseudogenes are classified as 'processed' where they have lost the parental gene structure and conversely 'unprocessed' ('duplicated') pseudogenes that retain the same exon-intron structures as their parent loci. If ambiguities arise, other features are used to resolve the provenance of a pseudogene.

B.3.3. ncRNA Annotation

To consistently characterize the non-coding RNAs across the three organisms, we took the perspective that, unlike protein coding genes, only a subset of ncRNAs, are annotated consistently across organisms. Thus we first built a subset of the annotations ("the comparable ncRNAs") that is directly comparable between the organisms. Next we added onto this set other ncRNAs that are well defined but inconsistently described between organisms. Finally, after removing all of these from the genome, as well as the exons from protein coding genes and pseudogenes, we have the fraction of the genome that is effectively un-annotated with respect to transcription, except for potentially being part of an intron. To find out how much transcription exists in this fraction we subjected each genome to uniform computational pipelines.

B.3.3.1. Uniform Comparison of Annotated ncRNAs

To consistently characterize the annotated ncRNAs and un-annotated transcription in the three organisms, we took the perspective that only a subset of ncRNAs, unlike protein-coding genes, are annotated consistently across organisms. Therefore we defined five biotypes for gold-standard annotations,

which are coding sequence (CDS), un-translated region (UTR), canonical ncRNAs (include miRNA, tRNA, rRNA, snRNA, and snoRNA), long non-coding RNAs (lncRNA), ancestral repeats for human, and unexpressed intergenic for worm and fly.

We made efforts to restrict the types of ncRNAs taken forward for further analysis mostly based on the consistency and comprehensiveness of their annotation across the three organisms. For human, most of these annotations correspond to GENCODE v10 [23], except for miRNAs, which are based on modified annotation derived from miRBase version 18 [24], see below. Ancestral repeats between human and mouse were extracted from 46-way vertebrates multiple alignment file. For worm and fly, gold standard ncRNA sets were obtained from the June 2012 data-freeze, again with the exception of miRNAs, which are based on a modified annotation derived from miRBase version 18, see below. For detailed summary see Table S2 and Fig. S2.

B.3.3.1.1. lncRNA

Human lncRNA sequences are annotated as 'lincRNA' by GENCODE, but we found these could be further divided into several subtypes depend on their genomic locations. lncRNAs overlapping a pseudogene or transposable element by more than one nucleotide were re-classified according to the overlapping RNA type. Antisense lncRNAs are those having greater than 50% of their sequences overlapped with known coding transcripts on the opposite strand. Intronic ncRNA fragments refer to ncRNA fragments fully embedded within an intron of a protein-coding gene on the same strand. lncRNAs found to overlap a known biotype but not fulfill the criteria for re-classification as stated above, are referred to as ambiguous lncRNAs. All the remaining IncRNAs that do not overlap any of the above annotations are by definition intergenic lncRNAs.

Worm lncRNAs were obtained from [25], while fly lncRNAs were extracted from FlyBase annotation "ncRNA" (with score > 0, meaning they were cDNA sequences or EST supported longer than 200 nt). Worm and fly lncRNAs were assigned subtypes in the same manner as the human IncRNAs. We also counted the nucleotides covered and assigned the gold standard annotations to 100 bp bins similar to the process in our previous work and count the corresponding bin numbers (Table S2) [26].

B.3.3.1.2. miRNAs

Diverse pathways generate short regulatory RNAs that associate with Argonaute effector proteins [27], including small interfering RNAs (siRNAs), microRNAs (miRNAs), and piwi-interacting RNAs (piRNAs). As clade-specific pathways generate many classes of siRNAs that are not directly comparable across these species, we focus our comparison on miRNAs. There are currently approximately 8 times as many pre-miRNAs annotated in humans (1,756) compared to either worms (221) or flies (235).

Human/fly/worm pre-miRNA hairpins were taken from miRBase v18 [24] and supplemented with human mirtrons annotated from [28]. Pri-miRNA (pri-miRNA) transcript annotations were collected from the literature and revised as necessary based on evidence. In many cases, the full pri-miRNA coordinates are not known, with the 3' end of the pri-miRNA beyond the mature miRNAs often incomplete. Our comparisons focused on intergenic "stand-alone" pri-miRNA transcripts, and excluded intronic

miRNAs whose primary transcripts might be coincident with protein-coding genes. This comparison indicates that intergenic pri-miRNA transcripts are substantially shorter in worm (~0.37kb) as compared to fly (7.4kb) or human (20kb). It should be noted that many intronic miRNAs have been suggested to be transcribed from internal promoters, independently of their host mRNAs [29, 30].

Human pri-miRNA annotations were from RefSeq and [31]. The latter study inferred miRNA TSS from ChIP-seq data of chromatin marks, and therefore did not have annotation of pri-miRNA TTS. We defined start position of a pri-miRNA from the midpoint of the TSS range. In case the middle point is larger than miRNA hairpin start position, a minimum value of the TSS range was used. The end positions of pri-miRNAs are the 3' end of miRNA hairpin bounded by the mature miRNAs. If a pri-miRNA locus was annotated by both RefSeq and [31], the RefSeq annotation was used. We manually inspected and removed loci lacking compelling support from ENCODE RNA-Seq and Chip-Seq data.

Fly pri-miRNA annotations were from [3, 32, 33, 34], where their start and end positions are the annotated TSS and TTS. Most of these annotations come from RNA-Seq data on total ribominus embryonic RNA [3]. As 3' Drosha cleavage products appear to be much less stable than 5' Drosha products, the latter are poorly represented in these annotations. A few other pri-miRNAs were identified from directed cloning [33] or inferred from in situ hybridization evidence [32]. We supplemented these with additional miRNA clusters that define minimum portions of other pri-miRNA transcripts [35], whose start and end positions are the 5'-most and 3'-most positions of encoded miRNA precursors.

Worm pri-miRNA annotations were from [36, 37]. In the work of Gu and colleagues [37], TSS were annotated mainly by CapSeq analysis, with CIP-TAP used if no CapSeq data was available. The TSS with the maximum CapSeq/CIP-TAP reads (and which is upstream of the miRNA hairpin 5' start position) was taken. The end positions were defined as the 3' ends of the miRNA hairpin from miRBase stem-loop annotation.

B.3.3.1.3. Other Short ncRNAs

Transfer RNA (tRNA) sequence annotations were obtained directly from each of the organism's annotation sets described above. These tRNA annotations are produced by the tRNAscan [38] and are consistent between human, worm, and fly. Similarly, small nucleolar RNAs (snoRNAs) were obtained from the GENCODE, WormBase, and FlyBase annotations, and are based on annotations derived at least in part from [39, 40, 41]. Small nuclear RNAs (snRNAs) are sufficiently well annotated [42, 43, 44, 45] in the three organisms to warrant inclusion in this analysis.

B.3.3.2. Non-Comparable ncRNA Annotation

Several types of non-coding RNAs were considered for the three species comparative analysis, but were ultimately not used due to non-trivial differences in the extent of the completeness of their annotation in one or more species. Such RNAs are biologically interesting and are worthwhile to study within the context of a single organism, however we deemed that there is currently either too little or too non-comparable annotation of the ncRNA types (including mitochondrial RNAs, piRNAs, rRNAs, Y RNAs, and misc_RNA) to be of significant value to this investigation.

B.3.3.2.1. Ribosomal RNAs

The sequence and structure of the human, worm, and fly ribosomal RNAs (rRNAs) have been known for some time [46, 47, 48]. However, the conventions adopted in annotating rRNAs in these genomes are inconsistent due to the large numbers of repeats of these sequences throughout the genome. For example, the human 45S rRNA precursor is annotated in UCSC on a clone contig (chrUn_gl000220) that cannot be confidently placed within the context of the canonical set of chromosomes, while it is known that there are multiple tandem repeats of the 45S precursor on several chromosomes in the human genome. Sequences for the human 5S rRNA, however, are comprehensively annotated in NCBI, UCSC, and GENCODE, with information about the coordinates of most, if not all, of the tandem repeats. As a result of this inconsistency of rRNA annotation between the species, we decided to omit analysis of rRNAs in this work for fear of being unable to confidently assign reads mapping to these variably annotated repetitive regions.

B.3.3.2.2. Piwi-interacting RNAs

We performed a cross-species annotation of piwi-interacting RNAs (piRNAs) by taking human intergenic piRNA clusters defined in previously reported annotations [49, 50]. As these were originally annotated from modest small RNA-Seq data (<50K reads), we inspected and re-annotated these loci with respect to ~100M human testis small RNA reads [5, 51] (GEO accession: GSM995304 and SRP006043). The original piRNA cluster annotations were deleted or refined to ensure that the called locus generated a majority of piRNA-sized reads from total RNA (e.g., >25 nt), and that there was nucleotide coverage of at least 40% of the inferred cluster by piRNAs. Finally, in order to focus the analysis on intergenic non-coding piRNA clusters, we subsequently removed clusters that could be attributed to 3' UTRs, or likely extensions of 3' UTRs [52]. Fly piRNA master loci were annotated from [53]. Worm 21U RNAs were annotated from [37, 54, 55]. All coordinates were converted (using UCSC liftOver and WormBase remap_gff_between_releases.pl) to hg19, dm3, and ce10 if different assemblies were used in the literature.

Using this procedure, we were able to confidently assign 88 human loci, 27 fly loci, and 35329 worm loci as piRNA clusters. The reason for the disparity in the numbers of observed loci stems from the fact that the molecular pathways that generate piRNAs (small RNAs associated with Piwi-class Argonautes [56]) are similar in flies and mammals, but radically different in nematodes. In the former, piRNAs are derived from long non-coding transcripts, transposable elements, and mRNA 3' UTRs, whereas in the latter, piRNAs ("21U" RNAs) are the products of extremely short transcription units. Given this difference in piRNA characteristics in the worm, as compared to human and fly, we felt it prudent to exclude piRNAs from further cross-species analysis.

B.3.3.2.3. Y RNAs

A study in which a homology search for Y RNAs was performed across 27 species identified very small numbers of Y RNAs in human and worm, and inferred evidence for Y RNAs in fly [57]. However Y

RNAs are currently not officially included in either the WormBase or FlyBase annotation sets so Y RNAs were excluded from downstream analysis.

B.4. Comparison of Protein-Coding Genes

We began our comparisons of by examining the basic characteristics of the protein-coding repertoire (Fig. ED3). Human and worm have ~20,000 genes whereas fly has about 2/3 of that number (Fig. ED3). Fly and worm genes span similar genomic lengths but human genes span larger regions (Fig. ED2). Most of this difference is due to the greater size of human introns, but longer human CDSs also contribute. Individual exon lengths have similar distributions between human and worm, whereas fly has some larger exons. Human differs most obviously from fly and worm, however, in the number of exons per gene. This is also reflected in the human genome containing more than twice as many coding bases and 5' UTR sequence as worm despite having the same number of genes.

Given the difference in CDS size and exon complexity between the organisms, we revisited the question of domain complexity raised in the initial genome papers [58, 59]. We found that <10% of the protein domains found in fly and worm were specific to each phyla, whereas nearly 20% of human protein domains were not found in the two models organisms (Fig. ED2, Fig. S1, and Associated Data File). While overall most domains are shared, we found that unique combinations of domains have substantially increased in human, with only 32% of human domain combinations shared by the other two organisms (Fig. ED2), whereas the corresponding numbers for fly and worm are 64% and 54%.

In analyzing the RNA-Seq reads, we found that overall the distribution of reads is quite similar in fly and worm with ~95% of the reads mapping to annotated protein coding genes (CDSs + UTRs). The majority of reads in human also derive from protein-coding genes, but the somewhat smaller fraction (78%) reflects the fact that there are much more sequences in human outside of protein coding segments as compared to worm and fly. The RNA-Seq data also reveals the fraction of genes in each organism broadly expressed across cell lines, tissues and developmental stages. Overall, we identified 6,912 (38%), 5,180 (25%) and 5,288 (38%) broadly expressed protein coding genes in human, worm, and fly respectively (Table S1 and Associated Data File). Across all three organisms, these genes tend to be more highly expressed and to have characteristic housekeeping functions, such as RNA processing, protein transport, and protein localization (Fig. S1 and Table S1).

B.4.1. Domain Combinations

All proteins from human, worm and fly transcriptomes were assigned protein domains by HMMER3 [60] using version 26.0 of Pfam domains (Fig. ED2 and Fig. S1). Domains with independent E-values greater than 10E-3 were filtered. Overlapping domains with same start sites or stop sites were filtered by removing the domain with higher E-value. To determine the domain structure complexity of each organism, we described each protein-coding transcript as a linear combination of domains, arranged by start sites of the domains determined by HMMER3. For example, for three distinct domains A, B and C, each combination A-B-C, A-A-B, C-A-B and A-A-A was considered as distinct domain combination while the permutations A-B-C and C-B-A are grouped together.

B.4.2. Broadly Expressed Protein-Coding Genes

We used RPKM values from 19 human cell lines, and 30 fly and 35 worm developmental stages to identify broadly expressed genes. Briefly, we calculated the coefficient of variation of logged normalized RPKM values for each gene. A threshold was then determined using a universal approach for the three species based on the distribution of the coefficient of variation. Genes with variability less than this threshold were identified as broadly expressed. A detailed description of the method is as follows.

In different samples, the numbers of expressed genes are likely to differ, which lead to differences in the absolute RPKM values assigned to a given expression level [61, 62]. We sought to normalize the RPKM values in each sample to account for this effect, using a similar approach to [61], under the assumption that a large number of broadly expressed genes will have on average the same expression levels in any two samples. The average RPKM value for each gene across different samples was determined and used as reference for normalization. We calculated the M values, defined as the log-fold difference in RPKM for each sample relative to the average, for genes that have RPKM>0.1 across all the samples. Instead of the trimmed mean of M values used in [61], we estimated the mode of the M value distribution by fitting a Gaussian within $-1.5<M<1.5$. The scale for each sample was then determined as $s=2^{(\text{Gaussian fit mean value})}$. As an example, the determination of the scale factor for HeLa-S3 cells is presented in Fig. S1. Finally, the normalized RPKM value for each gene is given as: nRPKM = RPKM/s. It should be noted that this normalization approach would give the same results for two samples where one has twice as high expression as the other for all genes. However, it is technically not possible, and for our purposes not desirable, to account for changes in genome-wide transcription level [62]. We find that this normalization procedure improves the sensitivity of broadly expressed genes identification (Fig. S1, Table S1 and discussion below).

We have assigned twelve different scores to each gene and assessed the sensitivity of these scores on identifying broadly expressed genes:
1. The minimum RPKM value across all samples,
2. The coefficient of variation of RPKM values across samples,
3. The coefficient of variation of $\log_2(\text{RPKM}+1)$ values,
4. An entropy score defined as: $\mathrm{S} = -\sum_i p_i \log(p_i)$, where $\mathrm{p_i} = \frac{\mathrm{RPKM_i}}{\sum_i \mathrm{RPKM_i}}$, and i denotes different samples,
5-8: the same scores as 1-4, based on nRPKM values, instead of raw RPKM.
9-12: The same scores as 1-4, based on TMM scaled RPKM values [61], instead of raw RPKM. The TMM scale factors were obtained using the calcNormFactors function in R/bioconductor package EdgeR using default parameters [63].

We have used chromatin features in mixed cell samples as an independent validation set in assessing these scores. Tri-methylation of lysine 36 on the histone H3 tail (H3K36me3) is an epigenetic mark associated with active transcription. Conversely, H3K27me3 is a mark that is associated with PolyComb mediated silencing. Therefore, genes expressed in the majority of cells in a mixed cell sample tend to show H3K36me3 enrichment and H3K27me3 depletion. However, for genes that are expressed in only

a subset of the cells, the H3K36me3 signal may be washed out and these genes may be repressed in the majority of cells and enriched for H3K27me3 mark [64]. We assigned genes enriched in H3K36me3 or depleted in H3K27me3 in fly and worm L3 samples as broadly expressed and vice versa. Even though this classification may not be completely accurate, it allows an assessment of different broadly expressed gene definitions. In particular, it provides a comparison of expression-based scores obtained from whole animals during developmental stages to a data set that is sensitive to tissue specific variation in a given stage. For both marks and both species, we found that the coefficient of variation of $\log_2$(nRPKM+1) showed the best performance in classifying broadly expressed genes (Fig. S1).

As an additional check, we investigated how stable each score is depending on the RNA-Seq samples used. We calculated all twelve scores described above for additional modENCODE RNA-Seq samples which were not included in this study. These sets include 72 strand-specific samples including cell lines and tissues for fly, and 45 samples including different treatment conditions for worm. We calculated the spearman correlation for each score between the developmental stages studied and these additional samples. For both species, we observed that the coefficient of variation of $\log_2$(nRPKM+1) has the highest correlation between two independent sample sets. The degree of correlation observed suggests that the list of genes we identify as broadly expressed using developmental stages is indeed a generally applicable list.

Based on the above observations, we defined broadly expressed genes based on the coefficient of variation of $\log_2$(nRPKM+1). The distribution of the coefficient shows a large peak at small variability for all three species (Fig. S1). Empirically, we found that the distribution around this peak is well described by a skew normal function for all three species (Fig. S1). We set a threshold at the coefficient value where the area under the skew normal fit drops to 99% of the area under the actual distribution of the coefficient of variation and defined the genes with variability less than this threshold as broadly expressed. The threshold for each species is marked with a vertical colored line in Fig. S1.

Gene Ontology (GO) terms for broadly expressed protein-coding genes were determined for each species using the getEnrichedGO function in R/bioconductor package ChIPpeakAnno [65]. P-values were adjusted for multiple hypothesis testing using the Benjamini & Hochberg step-up FDR-controlling procedure: (multiAdj = TRUE, multiAdjMethod = "BH"). The minimum number of terms for each GO category is set at 100: (minGOterm = 100). Terms for which the product of p-values for the three species is less than 10^{-100} and the individual p-values are all less than 0.01 are reported as top common GO terms.

The nRPKM scale and the threshold on the coefficient of variation, which were identified using coding genes, were applied to non-coding genes, pseudogenes and TARs directly to identify broadly expressed genes in these classes.

B.5. Splicing

With the great depth of sequence reads in the data sets, alternative splice junctions that are very rare relative to constitutive junctions were detected, perhaps reflecting imprecision in the splicing machinery. By design the annotation sets used give a conservative estimate of the number of alternative splicing

events for a given gene.

B.5.1. Comparison of Splicing In Annotation Sets

Using the protein-coding gene annotation sets described above, we compared the number of annotated isoforms per gene in the three organisms (Fig. ED4). (Note, by design the three annotation sets used give a conservative estimate of the number of alternative splicing events.) Consistent with recent reports [66, 67] we found that ~81% of human protein-coding genes express multiple mRNA isoforms. The corresponding fraction is similar in worm (~74%), but less in fly (~47%). However, fly has the greatest number of "outlier" genes that express a large diversity of isoforms. In particular, it has 100 genes with >50 isoforms. An extreme case is Dscam, which has >38,000 potential isoforms [68].

The proportions of different alternative splicing events are broadly similar between the three organisms. However, skipped exons are most abundant in humans while retained introns are most abundant in fly (Fig. ED4). This may be because in human, with large introns, exon definition predominates where the splicing machinery recognizes splice sites on either side of an exon [69], while in fly, the splicing machinery coordinately assembles on the splice sites on either side of an intron [70].

Another difference between the three organisms relates to mutually exclusive splicing. In fly, there are 12 mutually exclusive splicing events involving more than two exons. These range from clusters of three alternative exons to as many as 48 alternative exons in Dscam. In contrast, all mutually exclusive splicing events in human and worm involve only two exons. The mechanisms required to faithfully splice clusters of mutually exclusive exons containing only two and more than two exons are distinct [71], suggesting another fundamental difference in the splicing machinery of fly compared to that of human and worm.

As part of the resource we have also performed an extensive analysis of the relative abundances of the inclusions rate of the various splicing events between organisms using RNA-Seq data.

B.5.1.1. Comparison of Splicing of Orthologs

We compared alternative splicing events between the orthologous genes from human, worm and fly. Our short RNA-Seq reads can define individual splice junctions and exons, but do not allow unambiguous definitions across whole transcripts. Accordingly, we focused our analysis on junctions and exons. Overall, we did not find a conservation of splicing between orthologs, with no examples of orthologs preserving complete splicing structures across the three organisms. Moreover, there are only a few preserved splice sites in all three organisms (573 out of 37,517 unique splices in orthologs) (Table S1). Fig. ED4 depicts an example, the orthologous genes KCNMA1 (human), slo-1 (worm), and slo (fly). The exon/intron organization of the orthologous genes differs significantly between the three organisms, and there are no orthologous alternative exons.

To examine the conservation of splicing structures (Table S1), we considered the preservation of splice sites in orthologs across the three organisms. We started with 1935 1-1-1 orthologs, of which 1803 have isoform structure annotations. For each gene, nucleotide sequences of each transcript annotated in each

organism were extracted, and were aligned by Clustal Omega with default settings [72]. Splice sites in each transcript were then mapped onto the alignments. A splice site is considered as conserved between two organisms if its acceptor and donor positions in the multiple alignments are the same between at least one isoform from each organism.

B.5.2. Splicing Quantification Alignment Pipeline

We first aligned all poly(A)+ whole-cell RNA-Seq data for human, worm and fly using a uniform pipeline. Briefly, TopHat (version 1.4.1) [73] was used to align all reads to the transcriptome of each organism, reporting uniquely aligned reads and not allowing for novel junctions to be identified. The data used for splicing analysis included 22 cell lines and 16 tissues for human, 30 developmental stages, 29 tissues and 25 cell lines for fly, and 67 samples for worm. The reference genome assemblies for human, worm, and fly were hg19, ce6 and dm3, respectively. We restricted the set of splice junctions that could be aligned to those present in the human, worm, and fly annotation freezes. The command line parameters that were used for all alignments were -p 8 -z0 -a 6 -m 0 --min-intron-length 28 -g 1 -x 60 -n 2 --no-novel-juncs. In addition, the --library-type parameter was set individually for each library as appropriate. The --transcriptome-index parameter was set to the annotation gtf file for human, worm, or fly. After the alignments were completed, the bam files from biological replicates were merged using samtools [74]. Bam files for all samples of each organism were merged into a single bam file that was used to generate the data in Table S1. BedGraph files were generated using BedTools [75].

B.5.2.1. Splice-Event Parsing

MISO [76] is a Bayesian probabilistic framework that quantifies the expression levels of alternatively spliced genes from RNA-Seq data. We invoked MISO in its alternative splicing event level mode ("exon-centric" analysis) to gauge the relative frequency of splicing choices made within windows containing and defining various splice-event types; e.g., skipped exons, alternative first exons, etc.

From each coding-only GTF annotation file (for human, worm and fly), we constructed GFF3-based alternative event files for each event type (as required by MISO), where alternative events are defined by an enumeration of each alternative set of resultant, spliced exons within an event window.

In parsing GTF annotation files to construct specific event type alternative event files, it is convenient to first extract certain information from the GTFs that can facilitate the parsing task. These include constructing ordered lists of distinct exon and intron coordinates for each gene and collateral lists of Boolean strings for each gene, where each string indicates which exons and introns of the gene are present in a given transcript. Another useful derived list is constructed by conceptually projecting all exon boundaries of a given gene to the genomic coordinate axis. This partitions the gene extent into intervals ("gene features") that are either included or excluded in each of the gene's spliced transcripts—inclusion/exclusion status is recorded as a list of isoform Boolean strings for each gene along with feature-interval coordinates.

What follows is a very high-level description of our event parsing strategy. Rigorously defining the

various splicing event types can be a challenging task in itself, with experienced researchers often disagreeing as to whether a specific scenario qualifies as a particular event-type instance or not—to our knowledge there does not currently exist a set of generally-agreed-upon legalistic event definitions of the sort that can be trivially converted to scripts. Through a process of iterative refinement we have converged to, what we believe to be, a reasonable set of operational definitions that have been implemented as event-parsing scripts, though this continues to be an ongoing process and in some cases we have retained multiple parsing implementations so as to accommodate contrasting event-type definitions. Note that the resulting set of parsed events is neither exhaustive nor unique with respect to the exon set; exons may participate in more than one event type or none at all.

Skipped Exon (SE)

We have implemented two definitions ("conservative" and "liberal") for the cassette exon case. The conservative interpretation made use of the derived gene-feature representation and searched for instances of an alternative exon feature directly-flanked on either side by a constitutive intron feature and a bounding constitutive exon feature. The terms "constitutive" and "alternative" were interpreted with respect to all isoforms containing a given feature and were derived by considering column sums of the Boolean gene feature matrix over a subset of its rows. Conceptually, the script dragged a skipped exon event recognition template over (an appropriately row-summed version of) gene features. Finally, the bounding constitutive gene features were extended (flanking constitutive exon features were grown) to fill their maximal common constitutive extent.

The liberal interpretation made use of the Boolean exon and intron feature representations and coordinates. For each isoform of a given gene, our script considered each consecutive intron pair. For each other isoform it checked for the existence of an intron in its intron set corresponding to the excision of the exon implied by the original intron pair.

The conservative implementation entailed a relatively strict definition of constitutive flanking regions and alternative skipped exon, while the liberal case may be regarded as being consistent with the terminology "skipped exon." The liberal definition was used for counting events (Fig. ED4); and conservative definition was used for quantitating events using the RNA-Seq data (Fig. S1).

Mutually Exclusive Exon (MXE)

Generally, this event is defined by a window bounded by two constitutive exons, where the window's interior contains two or more alternative exons, with exactly one of these exons appearing in any particular isoform. Our implementation made use of the Boolean exon feature representation and coordinates for each gene. With windows defined by consecutive constitutive exons (determined by summing over rows of the Boolean exon feature matrix), the script considered alternative (as indicated by Boolean row-sum values) exons within the window interior and checked, using the Boolean exon feature matrix, that exactly one alternative exon within the window appears in each isoform (for each row, the sum of the Boolean exon feature matrix over the cluster-exon columns is equal to 1).

A “strict” interpretation further required that the alternative exons within a window cluster be mutually disjoint, while a more-liberal interpretation allowed the cluster exons to overlap (though with distinct start and end coordinates).

Coordinate Skipped Exon (CSE)

This event may be viewed as a generalization of the skipped exon event to multiple exons, or as a dual event to the mutually-exclusive case—all exons in a window flanked by constitutive exons are either included or not. Our implementation was a simple modification of the mutually exclusive script to require that, in each isoform, all alternative exons within a constitutive-exon-flanked window be either all included or all excluded.

Retained Intron (RI)

Our implementation used the Boolean exon feature representation and coordinates for each gene. For each distinct exon for a given gene (called “exon*”), we noted its start and end coordinates. Then for each isoform we checked to see whether there exists an exon with the same start coordinate as exon* and with end coordinate in the interior of exon* AND another exon (from the same isoform) with the same end coordinate as exon* and with start coordinate in the interior of exon*.

Alternative 5’/3’ Splice-site (A5SS and A3SS)

Our implementation used the Boolean exon feature representation and coordinates for each gene. Without loss of generality, it sufficed to consider the cases of alternative 5’ splice-site for a positively-stranded gene—the other cases follow by symmetry.

For each gene, the script looped through its isoforms, and for each exon-exon splice, it entered the two exons’ start and end coordinates into a hash, resulting in a list of all distinct exon-exon splices (distinct with respect to participating exon start and end coordinates).

The keys of the hash are ordered {start1}{start2}{end1}{end2}, where exon1 is geometrically located to the left of exon2. Then, for each fixed start1 key, we considered each start2 key and cases in which the number of end1 keys (for fixed start1 and start2) is greater than 1—these multiple end1 keys were taken to be the alternative 5’ splice-sites. The minimum end2 associated with these fixed start1 and start2 and multi-end1 splices was taken to be the right endpoint for the event window (start1 is the left starting point for the window).

Alternative First/Last Exon (AFE/ALE)

Our implementation used the Boolean exon and intron feature representations and coordinates for each gene. Without loss of generality, it sufficed to consider the cases of Alternative First Exon for a positively-stranded gene—the other cases follow by symmetry.

The script began by constructing a hash that, for each gene, associates introns to their left and right exons—hash keys are ordered {intron_start}{intron_end}{left_exon_start}{left_exon_end}{right_exon_start}{right_exon_end}. We also constructed a hash for first-introns—where hash keys are ordered {intron_end}{intron_start}.

For each first-intron intron_end, if there existed more than one associated intron_start, then for each intron_start from the intron_to_left_and_right_exon hash, considered each associated left_exon_start and right_exon_end, and accumulated the max left exon start and min right exon end. These coordinates define the boundaries of the event window. The events identified were then further filtered for those that had at least two unique start (AFE) or end (ALE) sites.

Tandem UTR

Our implementation used the Boolean exon and intron feature representations and coordinates for each gene. Without loss of generality, it sufficed to consider the cases of Tandem 3' UTR for a positively-stranded gene, with the negatively-stranded cases follow by symmetry.

For each gene, the script constructs a hash of distinct rightmost exon start and end coordinates over all isoforms. The hash keys are ordered {exon_start}{exon_end}. For each fixed exon_start, we register Tandem UTR events simply as cases where there exist multiple associated exon_ends.

B.5.2.2. Splicing Quantitation

We investigated how alternative splicing varies quantitatively between samples (distinct conditions, tissues or developmental stages) and organisms. Because of the limitations of short-read RNA-Seq noted above, we have only quantified individual splicing events consisting of an alternatively spliced exon and its immediate flanking constitutive exons. Furthermore, we only analyzed unambiguous events in which other annotated transcripts could not confound the interpretation and the events involve only two possible alternative splice-forms. To facilitate a consistent quantification, we uniformly processed the matched compendium with a splicing focused pipeline, calculating the percent inclusion of the alternative exon in each sample. We used MISO [76] to quantitate the percent inclusion for all alternative splicing events described above (e.g., cassette exons, alternative first exons, etc.) in all three organisms. Briefly, the GFF3 files for each splicing event described above were indexed using the index_gff.py script from MISO, quantitated using the run_events_analysis.py script in single read mode, and summarized using the run_miso.py script with the --summarize-samples option.

The majority of events in each species are either primarily included or skipped (Fig. S1). However, when examining each individual event class, there are some distinctions. For example, most skipped exon events in each organism have one splice-form that greatly predominates, though the nature of that differs between organisms. In particular, skipped exons are most often absent in fly and present in worm (Fig. S1). For tandem UTR events, human has a striking peak at an inclusion value of ~50% indicating that the long and short forms of the UTRs are expressed at equal levels on average.

We next examined the dynamics of alternative splicing for each splicing event by calculating the maximal change in percent inclusion over all pairs of samples for each species. We call this quantity the "switch score" and binned the splicing events into those that varied strongly (switch score >50%), moderately (25-50%), or weakly (0-25%). The majority of splicing events in fly and worm change dramatically between samples (Fig. S1).

Finally, we examined the conservation of the sequences associated with splicing, specifically skipped exons and their flanking introns. In all three species, the strongly varying exons (those associated with the highest switch scores) and the adjacent portions of their flanking introns are more conserved than moderately varying exons, and the exons that vary the least are also the least conserved (Fig. S1).

C. More Details on “ncRNAs & Non-Canonical Transcription”

C.1. Context for “ncRNAs & Non-Canonical Transcription”

The study of pervasive transcription – i.e. that a significant fraction of the genome is transcribed both in the form of primary and processed RNA has mostly been study in the context of the human genome [16, 77, 78, 79]. For example, recently, around 160,000 transcription initiation complexes are identified on the human genome associated with non-coding transcription[80]. This result has been quite controversial and been the subject of much debate [81, 82, 83]. Pervasive transcription has had limited investigation in other organisms, e.g. in worm [84, 85] and in fly [3]. It has had even more limited study in terms of cross-species comparisons; for example in [86], the authors compare the pervasive transcription in fly and human genomes using tiling microarrays and identified significant pervasive transcription in both species. Recently, an additional study was done in [4], where authors sequenced polyA+ and small RNAs from 6 tissues in 10 mammals and studied the evolution of the gene expression levels between different species, organs, and chromosomes. The authors detected 54% of the human genome to be expressed and at least 30% of any other genomes that they studies was detected to be transcribed. Even in this study the size of the generated data is only ~5% of what the modENCODE/ENCODE consortia generated, allowing much less detailed ncRNA analysis. Furthermore, no total RNA data sets were generated.

The modENCODE project offers the opportunity for the comparative studies since the size and quality of the RNA-seq compendium gives us the statistical power for uniformly detecting and comparing the novel pervasive transcription.

In this paper for the first time using the matched ENCODE RNA resource we are able to consistently compare the amount of pervasive unannotated transcription (non-canonical transcription) between the genomes of two model-organisms with the human genome. We have developed a novel analysis

methodology for the consistent comparison of the amount of transcription across three highly diverse genomes. For the first time we observe a consistent amount of unannotated transcription (as a fraction of the genome) in the human genome as compared to the worm and fly genomes. This novel result is even more striking given the large evolutionary separation and distinct genome architecture of the three genomes compared. Thus, we demonstrate that the phenomenon of pervasive transcription is not specific to humans but a general principle of transcription that is shared by metazoans. This is important as it addresses the on-going debate.

C.2. Consistent ncRNA Pipeline Processing

In order to avoid biases introduced because of differences between samples and varying sequencing depth for data sets from each the three organisms, we developed a methodology for uniformly identifying the unannotated transcribed regions. It is, however, not straightforward to do this comparison since the organisms are substantially diverged from each other. In addition, the conditions, developmental stages, and cell types are not directly comparable between the organisms. Finally, it is necessary to correct for the read coverage differences between different RNA-seq experiments.

In order to perform this comparison we start with all the available poly(A)+ whole-cell RNA-Seq data that was generated for worm, fly and human by the ENCODE and modENCODE consortia (see Associated Data Files). We start with the RNA-seq reads and uniformly align them using Bowtie [87] against the reference sequence and splice-junction library for each organism. In each organism we pool the aligned reads across all experimental conditions (i.e. cell lines, developmental stages).

C.2.1. TAR Calling

We first started with the pooled RNA-Seq data sets derived from only polyadenylated RNAs. Reads that mapped uniquely were used to generate the signal tracks. The signal tracks are then segmented into TARs using thresholding and filtered as per min-run parameter and merged using a maximum gap parameter. We covered a large range of values for the 3 parameters so as to identify a large set of TARs with different coverage. The TARs regions can be found in Associated Data Files F.2.1.

In order to identify the best set of parameters for TAR calling, we built receiver operating characteristics (ROC) curves to visualize the dependency between the fraction of the detected exons versus the fraction of the “non-canonical” transcription (in regions except for protein-coding exons, annotated ncRNAs or pseudogenes) To compute these quantities for each TAR set, we used following procedure: As a ground truth of the known expression, we used the protein-coding exons with expression higher than 0.01 RPKM so as to exclude the exons that are not expressed. This very low RPKM threshold is used only as a prefilter to remove the very lowly expressed coding exons that would interfere with the threshold/min-run/max-gap parameter selection procedure. When we apply this threshold, the percentage of exons that pass is 92% for human, 98% for worm, and 99% for fly. In addition, when this threshold is scaled with the number of reads, there are reasonable number of reads for each species: 70 reads per kilobase for human, 17 reads per kilobase for worm, and 100 reads per kilobase for fly. For each parameter set, we overlapped the corresponding TARs with the ground truth set of exons to identify the discovered exons.

The fraction of coverage of discovered exons to all ground truth set is the exon discovery rate. Next, we subtracted the set of exons of known protein-coding genes, pseudogenes, and non-coding RNAs from TARs to identify the non-canonical transcription. The fraction of coverage of the non-canonical transcription to the whole non-heterochromatin genome size is used as the fraction of non-canonical transcription. Since different parameter sets may yield the same exon discovery rate, we selected the TARs with the smallest coverage (smallest non-canonical transcription rate) to have the most conservative set of TARs.

The ROC curves are plotted for the three species and are shown in Fig. S2. For generating a comparable set of TARs between all species, we selected a conservative and a relaxed exon discovery rate (90% and 98%) and identified the unannotated exon discovery rates for these parameters. We quantified the expression values for the novel TAR expressions and observed that the TARs are expressed at much lower levels compared to the protein-coding genes (Fig. S2). In addition, only ~1% of the TARs are found to be broadly expressed in each of the three organisms (Table S1). More detail on the TARs with respect to genomic location are included in Tables S2.

C.2.2. Calibrating PolyA+ RNA

In order to assess whether our use of poly(A)+ library prep for RNA-Seq analysis would limit our ability to observe non-coding elements of the human, worm, and fly transcriptome, we leveraged matched human RNA-Seq data derived from poly(A)+ and long/short total-RNA preps produced as part of the human ENCODE phase 2 project. Specifically, GM12878 and K562 while-cell RNA-Seq data were obtained from the ENCODE RNA dashboard (http://genome.crg.es/encode_RNA_dashboard/hg19/). BAM files containing uniquely mapped reads from Long Poly(A)+, Long TotalRNA, and Short TotalRNA sample-preps were downloaded and intersected with pre-miRNA, tRNA, snRNA, snoRNA, lincRNA, and exonic-mRNA coordinates derived from the GENCODE v10 annotation. Read-counts for each sample were simply normalized by total number of uniquely mapped reads (reads-per-million); we decided against normalizing to RPKM as a comparison between annotated features within each data set was not required. The distributions of lengths of each type of RNA assessed (Fig. S2) were used to determine the ‘optimal’ method of sample preparation that one would typically choose to assess its expression. For miRNA, tRNA, snRNA, and snoRNA, with lengths averaging 100nt, the optimal sample preparation would be short-totalRNA; for longer and not necessarily polyadenylated lincRNAs the optimal preparation is long-totalRNA; and for mRNAs either long-totalRNA or poly(A)+. An assessment of the variability in expressions measured between the K562 and GM12878 cell lines from data prepared in the ‘optimal’ manner for each RNA type is provided, for reference, in Fig. S2.

The effect of poly(A)+ RNA purification during sample-preparation on our ability to detect coding and non-coding RNAs in the K562 cell line is shown in Fig. S2 and in the GM12878 cell line is shown in Fig. S2. It is clear in both cell lines that poly(A)+ RNA-Seq performs very poorly, compared to short-total RNA-Seq, at detecting miRNAs, tRNAs, and snRNAs. However, the poly(A)+ RNA-Seq expressions obtained for snoRNAs, lincRNAs, and mRNAs correlate much better with abundance estimates obtained from short-totalRNA, and long-totalRNA sequence data. Given this result we proceeded with calling TARs and further characterization of lincRNAs in the three species using the poly(A)+ data available.

C.3. Consistency with Previous ENCODE Estimates

C.3.1. Consistency of ROC Analysis and IDR Thresholding

Since we did not have replicate information for worm and fly, we performed this analysis only in human samples where we have biological replicates. Our results show a significant reproducibility of the TARs in human.

The differences in coverages calculated using contigs from IDR and ROC based TAR method are due to the fact that the ROC based method used only whole cell poly(A)+ samples, while the IDR method used all samples from human Jan-11 freeze. We have re-calculated the contigs' coverage values in Table S10 from Djebali et al. 2012 [16] using just the whole cell poly(A)+ samples. If we take 5 reads per contig as a cutoff (corresponding to IDR<0.1), we get 42.8% coverage by contigs from whole-cell poly(A)+ samples compared to 66.0% for all samples. Note, that 3.3% of the coverage is overlapping with annotated exons, so the unannotated contigs coverage is 39.5%. This is fairly close (albeit still higher) to the 32.3% of "all-inclusive" (98%) TAR coverage from Fig. ED3. We think the remaining discrepancy is mostly due to the difference in methodology used to do the calculation. Since our method first pools the reads from all samples before making TARs, we expect to find fewer cell line specific TARs than methods which do not pool the reads beforehand, like in Djebali et al 2012. This was actually confirmed by comparing our TARs to their contigs on the same set of poly(A)+ whole cell samples. While our TARs are almost entirely a subset of their contigs (91.4% of our TARs overlap their contigs), our TARs are less cell line specific. Indeed if we partition their contigs into the ones overlapping our TARs and the ones not overlapping our TARs, we find that 36.3% of the first set is found in only 1 cell line, while 91.4% of the second set is found in only 1 cell line.

We estimated the reproducibility of the TARs identified with the relaxed threshold (98% exon discovery rate) via IDR analysis [88]. For the IDR analysis, we utilized the replicates for 19 human samples. For each cell line, we computed the expression levels (RPKM) of the TARs using the RNA-Seq data for the replicates then we performed IDR analysis on all the pairwise combinations of the replicates, resulting in total 19 IDR computations. The TARs for which smallest IDR among all the pairwise computations is smaller than 0.1 are flagged as passing the IDR filtering. We identified that with this filtering, 97% of the TARs (in coverage) pass the IDR filtering and are reproducible. For worm and fly, we did not have the replicate data for all the data sets thus we were not able to perform the reproducibility analysis. The estimate of reproducibility for human TAR regions, however, should be a surrogate for the reproducibility for worm and fly TARs.

C.3.2. Increase in Coverage Using Total RNA and Compartments

In this paper we focused on data that was comparable across the three organisms, focusing on long poly(A)+RNA-Seq data from whole cells. The results of Djebali et al. 2012 used many more data sets than those used here. When they only include the poly(A)+ RNA-Seq used here they only got 42.8%, however, when all the data including cellular compartments and total RNA is included it increases to

66%. The estimation of the amount of non-canonical transcription represents a lower bound on the amount of transcription in all three genomes.

C.4. Supervised ncRNA Predictions

We applied a previously developed machine learning method, incRNA (Fig. S2) [26] to the whole genomes of human, worm and fly. This method is abbreviated "incRNA" which begins with "i" and is distinct from "lncRNA" which begins with "l". The supervised ncRNA prediction method performs very well in all three species (the AUC of ROC is 0.97~0.99), either using canonical ncRNAs or lncRNAs as the positive training set (Fig. S2). The predicted novel ncRNA candidates from the supervised ncRNA models (Table S2) were classified as ncRNA Type 1 (trained on canonical ncRNAs) and ncRNA Type 2 (trained on lncRNAs from the gold-standard sets). The incRNA predictions of lncRNAs were not as accurate as canonical ncRNAs because the current lncRNA gold-standard annotations were mostly derived from RNA-Seq assemblies or cDNA libraries, which were not fully studied or confirmed as noncoding transcripts. The novel predicted ncRNAs can be found in Associated Data Files F.2.3.

C.4.1. The Gold Standard ncRNA Data Sets

The following genomic elements are selected as gold-standard annotation sets for supervised ncRNA prediction models: confirmed coding sequences (CDS), un-translated regions (UTR), canonical ncRNAs (i.e. miRNA, tRNA, rRNA, snRNA, and snoRNA), long noncoding RNAs (lncRNA), ancestral repeats and unexpressed intergenic regions.

In human, the gold-standard annotations come from GENCODE v10, in addition to the miRNA annotations from miRBase V18. The ancestral repeats between human and mouse were extracted from 46-way vertebrates multiple alignments in UCSC genome browser.

In worm and fly, the ncRNA annotations discussed earlier, in addition to the miRNAs from miRBase V18. In addition to these, we added novel worm lncRNAs from [25]. The gold-standard long ncRNAs (> 200nt) of fly were extracted from the ncRNA annotations supported by cDNA or EST (Fly Base score > 0).

The ncRNAs can be further grouped into sub-classes. If more than 50% of a ncRNA is overlapped with coding exons on the opposite strand, it will be grouped into antisense ncRNAs. If a ncRNA is fully embedded in a coding gene's intron on the same strand, it will be grouped into intronic ncRNAs. If a ncRNA is overlapped with any known genomic elements (i.e. CDS, UTR etc) but did not fulfill the cutoff above, it will be grouped into ambiguous ncRNAs. The remainders will be grouped into intergenic ncRNAs.

C.4.2. Data Sets and Predicted ncRNA Filter, Annotation and Validation

Many high throughput data sets from ENCODE and modENCODE consortia were integrated in the supervised ncRNA prediction models: all available expression data which includes poly(A)+ RNA-Seq, poly(A)- RNA-Seq, small RNA-Seq; histone modification ChIP-seq or ChIP-chip data including various modification types (i.e. H3K4me3, H3K36me3,H3K27me3, etc). Subsequently, the predicted novel ncRNAs from the supervised ncRNA predictions were further merged and filtered. First, we removed the predictions overlapped with the exonic regions on the same strand or with known ncRNAs on either strand. Secondly, we classified the novel ncRNA candidates into several types based on their genomic locations: antisense, intronic, ambiguous and intergenic ncRNAs (Table S2). Moreover, we also compared the current supervised ncRNA predictions trained on the whole genome with the previous *incRNA* predictions trained on the conserved regions of worms. It should be noted that we do not refer to the name of the supervised prediction methodology as *incRNA* in the main text to avoid confusion of the name with the non-coding RNA biotypes. The current supervised ncRNA predictions covered most of the previous predictions (Table S2). To further validate the predictions, we carried out RT-PCR experiments in both fly embryos as well as various human tissues, and found most of the candidates were expressed (Fig. S2). Further validations of related ncRNAs predictions have also been carried out independently in all 3 organisms [2, 16, 26, 88, 89].

C.5. Relationship of HOT Regions and Enhancers to Non-coding Transcription

Many of the novel TARs and ncRNA predictions overlap with identified potential enhancers from Ho et al., (2015) [64]. Specifically, we found that 128,400 predicted enhancer regions in human overlap with novel TARs, and 15,863 and 10,380 enhancer regions in worm and fly overlap, consecutively, with TARs. To test whether these overlaps are statistically significant in each organism, we randomly shuffled all the enhancer annotations within the genome, and examined how many randomized regions overlap with TARs. This procedure was repeated for 1000 times. We then compared the observations from the real data with the randomization results, and calculated the z-scores and associated p-values of the observations. This test shows in all three organisms, the enhancers are significantly enriched for novel TARs (Fig. ED5). Similarly, we found the enhancer regions are significantly enriched for ncRNA predictions as well (14,357, 590 and 229 enhancers overlap ncRNA predictions in human, worm and fly, respectively - Fig. ED5). These novel TARs and predicted ncRNAs could represent the so-called enhancer RNAs (eRNAs), i.e. RNAs that are independently transcribed from enhancer regions [90]. (For consistency, we also constructed an eRNA set based on the enhancer set from the human ENCODE 2012 rollout[91].)

We also studied the overlap between the novel TARs and ncRNA predictions with the HOT (high-occupancy target) regions, which are regions that have an overrepresentation of different TFs binding sites [2, 91, 92]. In particular, we focused at the distal HOT regions, which are beyond 1kb upstream of the annotated transcription start sites. This is to avoid the transcription signals from HOT regions coupled with gene transcription. Using the same randomization method as introduced above, we found that the

distal HOT regions are significantly enriched for TARs (23,073 in human, 520 in worm and 435 in fly) and ncRNA predictions (4,604 in human, 49 in worm, and 7 in fly) (Fig. ED5).

TF binding sites distal to genes (i.e. enhancers) have been associated with RNA expression [90, 93]. We characterized some of the non-canonical transcription that contributes to this effect from enhancers [16, 64] and distal HOT (high-occupancy target) regions [94]. HOT regions have an overrepresentation of different transcription-factor binding sites [2, 91, 92] and have previously been suggested to be associated with transcription; distal HOT regions are the subset of HOT regions that are not in promoters. We overlapped both distal HOT regions and enhancers with our TARs and supervised ncRNA predictions and found a strong, statistically significant overlap in all three organisms. We then annotated the ncRNAs contributing to this overlap. This could represent "enhancer RNAs", i.e. RNAs that are independently transcribed from enhancer regions [90].

C.6. Mapping ncRNAs and TARs to Modules

With the TARs and non-coding RNAs uniformly characterized, we are now in a position to study how these elements function together as evinced from expression correlations over our many samples. Like conventional clustering analysis, our cross-species modules can be used to infer biological roles for genes (Fig. S2). See Suppl. D for discussion of the modules. For each species, we mapped its ncRNAs and TARs to modules based on co-expression correlations, and found those highly mapped ncRNAs may have related functions with modular genes so that we can annotate them based on modular functions. We have implemented our study as follows. First, for each module, we mapped the ncRNAs/TARs whose expression patterns are highly correlated or anti-correlated with at least one orthologous gene in the module (see correlation thresholds in Table S2). We then identified ncRNAs/TARs across species that are potentially functionally “orthologous” (analogous) by looking for those mapped to ortholog-enriched modules. We found a few examples with biological evidence to support that they function similarly in gene regulation (Fig. ED5, S2). Specifically, the 16 conserved modules cluster with 1706, 79, and 701 annotated ncRNAs and 8598, 9029, and 4750 TARs in human, worm, and fly, respectively. The co-expression of these ncRNAs and TARs with orthologous genes suggests that they might play related cellular roles. (We provide module annotations and associated GO terms for ncRNAs and TARs in the three organisms in the Associated Data Files F.2.2 and F.3.1)

D. More Details on “Expression Clustering & Stage Alignment”

D.1. Context for “Expression Clustering & Stage Alignment”

D.1.1. Expression clustering

There are many gene-expression clustering algorithms [95, 96, 97, 98, 99, 100]. These are based on a variety of underlying approaches (e.g. hierarchical clustering, PCA, etc). While these methods have provided valuable biological insights, they were mostly aimed at analysis within an individual species only. There are studies that integrate the expression profiles across various species. For instance, ref. [4] used the profiles to reconstruct expression phylogenies It focused on the distance between species but not on individual genes. Ref. [101] explored the conservation of co-expression links across species, but did not attempted to look at how these links form high-order structures such as clusters. Following the thought process of ref. [101], our work explored the conservation of co-expression patterns, but with a focus of modules.

D.1.2. Stage alignment

Though D. melanogaster and C. elegans are two well-studied model organisms, comprehensive characterization of the conservation in gene expression during development and cell differentiation between these organisms is lacking, as most data are generated in relatively small sets of cell lines, tissues using various different techniques. There are genome-wide mRNA expression profiling surveys shown that gene expression changes accompany morphological changes in the development of both D. melanogaster and C. elegans [11, 14, 102, 103, 104, 105, 106, 107, 108, 109]. Such studies have observed similarities in gene expression between some D. melanogaster early and late developmental stages [108], between some cell lines from D. melanogaster female adults and early embryos [18], and between C. elegans cells and their corresponding developmental stages [110]. To our knowledge, there exists no comparison of the whole life-span developmental expression profiles of fly and worm, especially no alignment has been found between the life-spans of these two species. McCaroll et al [14] only compared the genomic expression patterns in adult stages of fly and worm. Here, we provided a comprehensive comparison of multiple developmental stages between the two species which has not been conducted before (Fig. 1, Fig. ED7, Fig. S3, and Suppl. D.4). We further integrated the mapping with the hourglass hypothesis (below), and found that hourglass genes play an unique role in the alignment.

D.1.3. Analysis of the phylotypic stage

The published work regarding the molecular mechanism of phylotypic stage can be roughly classified into two categories.

The first category is centered on evolutionary analysis, namely “conservation of transcriptome” [111] and “stabilizing selection on gene expressions” [11]. These studies conclude that a group of highly conserved genes are expressed at a “stable”/“conserved” level during the phylotypic stage across different species. These genes underpin the molecular mechanism of phylotypic stage. These works provide us clues that cross species conservation is related with the phylotypic stage.
The second category focuses on the dynamics of gene expression, particularly the activation of key developmental regulators, during embryonic development [10]. Based on the transcriptome dynamics analysis, the authors observe that the embryonic development is segmented into a set of stages across 5

different Drosophila species. Due to the developmental timing species-specificity, the duration of the each corresponding stage varies in different species, leading to gene expression divergence among different species. However at one particular stage, the authors find that gene expression is consistent among all species. This work highlights that transcriptome dynamics follows similar patterns in all species, but the degree of transcriptional variation is dependent to the stage timing variance. The phylotypic stage stands out as a step where gene expression is conserved among different species, independent of its duration. The authors describe transcriptome divergence from the viewpoint of the within species transcriptome dynamics.

D.2. Constructing Co-expression Modules

An overall schematic of our cross-species clustering analysis is shown in Fig. S3 with details in this section.

D.2.1. Transforming Expression Profiles into Co-Expression Networks

Co-expression networks were separately constructed from the Pearson correlation matrices of the three species. We employed a local rank-based algorithm [112]. Given an N-by-N correlation matrix (Pearson correlation) for N genes in a species, each gene is connected to the top d genes with the highest values (absolute value) of correlation with it. If d is very small, the resultant network cannot form a giant connected graph. The value of d is chosen to be 5, which is the minimal number such that all three networks form a connected graph. Under this construction, though the number of nodes and edges in the three networks vary, the average number of links per node is similar (8.2 for human, 8.0 for worm and 8.2 for fly). In this analysis, the co-expression networks are not weighted, but we allow the edges to have positive and negative signs. For a given edge, either a positive (+1) or a negative sign (-1) were incorporated based on the sign of the Pearson correlation between two genes.

D.2.2. The Potts Model

We mapped our multiplex network to a coupled Potts model in which nodes can take spin values from 1 to q, standing for labels of different modules. A node represents a gene in one species. A pair of orthologs is represented by two nodes in two networks. q is a parameter chosen at the beginning as the maximum number of modules allowed. In this analysis, q=250. It was determined based on the size of clusters and genomes. In general, the exact value of q is not very important since small clusters arrived from a large value of q will be absorbed. An energy function is defined as the negative of a generalized modularity, written as:

$$-\left\{\sum_{i,j\in S_k}\left(A_{ij}^{S_k+}-p_{ij}^{S_k+}\right)\delta(\sigma_i,\sigma_j)+\sum_{i,j\in S_k}\left(A_{ij}^{S_k-}-p_{ij}^{S_k-}\right)\delta(\sigma_i,\sigma_j)+\kappa\sum_{(i,j')\in O(S_1,S_2)}\delta(\sigma_i,\sigma_j)\right.$$
$$\left.+\kappa\sum_{(i,j')\in O(S_1,S_3)}\delta(\sigma_i,\sigma_{j'})+\kappa\sum_{(i,j')\in O(S_2,S_3)}\delta(\sigma_i,\sigma_{j'})\right\}$$

Here, S_k (k=1,2,3) stands for human, worm, fly respectively. The expression inside the curly bracket is the modularity function for an individual signed expression network [113, 114], where A+ and A- are the adjacency matrices representing positive and negative links of a co-expression network. σ_i is the spin state of node i. The first two terms sum over the matrix elements of positive or negative parts of the signed adjacency matrices of the three species. p_{ij} is a null model of interaction, commonly defined as $p_i \bullet p_j/(2m)$, where m is the number of edges in the corresponding network, i.e. either the positive or the negative parts of an individual co-expression network. The extra terms represent the coupling (with coupling constant κ) between human-worm, human-fly, and worm-fly respectively (see the determination of κ in the next paragraph). The three sums sum over the three sets of orthologous pairs, represented by $O(S_1,S_2)$ etc. δ is the delta function. A high value in such a generalized modularity means that the three individual networks have high modularity, and nodes from different species in the same modules tend to form orthologous pairs. To take into account of the fact that many orthologous pairs are not one-to-one but many-to-many, the contribution of a pair of orthologs to the generalized modularity function is not 1, but normalized by the number of orthologs. For example, if gene W from worm is orthologous to gene H from human together with two other human genes, while at the same time gene H is orthologous to W as well as three worm genes, the weight assigned to the link between W and H will be (1/3+1/4)/2. For simplicity, this modification is not displayed in the above equation.

Every spin configuration corresponds to a way of assigning nodes to modules. The optimal assignment is the ground state of the system.

To determine the coupling constant κ, we employed a set of human, worm, fly triplets as a gold standard [101]. For each triplet, we examined whether the 3 components belong to a same module. In general, if κ is high, a high fraction of triplets will satisfy the criterion, but the modularity of individual networks will be low. This is because a node is strongly affected by its orthologs, rather than its neighbors in its own network. On the other hand, if κ is low, the modularity of individual networks will be high but the fraction of triplets satisfying the criterion will be low. By examining a range of values of κ, we balanced the tradeoff.

D.2.3. Simulated Annealing and Defining Confident Modules

To find the ground state, we employed a standard simulated annealing procedure very similar to one used in [115]. Spin values were randomly assigned initially, and updated via a heat bath algorithm. The initial temperature was chosen in a way such that the flipping rate (the probability that a node changes its spin state) is higher than 1-1/q. We lowered the temperature gradually with a cooling factor 0.9, until the flipping rate was found to be less than 1%. The resultant spin configuration was used as an

approximation to the actual ground state. Due to the probabilistic nature of simulated annealing, we repeated the annealing process 32 times, and represented the results by a co-appearance matrix as shown in Fig. 1. The matrix element represents the number of times in which a pair of genes (they could belong to 2 different species) are assigned to the same module. A confident score can be defined for a pair of assignment. To ensure the accuracy of the module assignment, we employed a stringent threshold where two nodes are assigned to the same module only if they have the same spin value in at least 95% of trial. Algorithmically, a module could be obtained by starting from a particular node, iteratively searching for neighbors that co-appear in at least 95% of the trials.

D.2.4. Further Clustering of the Conserved Modules

Instead of using all the protein-coding genes from the three species, we focused on a set of conserved genes (5,575 human genes, 4,486 worm genes, and 4,349 fly genes) that form 1-1-1 orthologs in the three species, as compiled by the consortia. In other words, given a human gene in the set, there exists a worm gene and a fly gene such that the three genes are mutually orthologous to each other. The result of the Potts model on this set of genes is shown in Fig. S3. The clustering algorithm arrived at 16 modules consisting of at least ten 1-1-1 triplets (Fig. 1 and Table S3). In general, modules with high 1-1-1 triplets signify a slow gene duplication rate that means they are more conserved (Fig. 1 and Fig. S3). The details of the 16 modules including gene names and enriched GO terms are shown in Associated Data Files:

genelist_16modules.xlsx,
GO_16modules_biological_process.xlsx,
GO_16modules_cellular_component.xlsx,
GO_16modules_molecular_function.xlsx.

D.3. Identification of Hourglass Behavior

D.3.1 Methods: Quantifying Modular Expression

For the study of the inter-organisms hourglass behaviour we used the microarray expression data sets across 6 fly species [11]. In the analysis of the intra-organsims hourglass behavior we used modENCODE RNA-seq embryo development data.

Using our cross species network clustering algorithm, we identified about 1,700 genes that co-evolved in both worm and fly, and clustered them into 16 modules. The expression levels of these modules were calculated using the following method. Given the gene expression matrix (N genes by m stages) in a module, $X=\{x_{ij}, i=1,2,..,N, j=1,2,..,m\}$, we calculated its first right singular vector, called "eigengene", $\{v_j, j=1,2,..,m\}$ of X via singular value decomposition (SVD), which can be considered as a normalized average expression vector with vector norm of one. We then defined the modular expression level at stage j as v_j. The eigengene was calculated on normalized expression of all genes of a module covering all developmental stages. To be precise, in the calculation of the eigengene, we did not presume the existence of a phylotypic stage.

D.3.2 Results

D.3.2.1 Identification of Inter-organism Hourglass Behavior

We found that the modular expression levels in 12 out of 16 modules have minimal variances across 6 fly species during phylotypic stages (Fig. ED6 and Fig. S3), which follow the hourglass pattern. However, this pattern was not obvious in the other 4 modules.

D.3.2.2 Identification of Intra-organism Hourglass Behavior

We found that among the fly modules, the divergence of gene expression at the phylotypic stage is minimal. This is an indirect evidence showing that modules in phylotypic stage are tightly regulated. As we have a time-course with higher resolution in worm, we studied how these modules are coordinated and regulated at each embryonic developmental stage by calculating the correlations between segments of module's eigengenes using a 2-hour sliding window (Fig. ED6C). Indeed, starting at 6 h of embryonic development, the genes' expression across all modules are strongly correlated, and this correlation persist for 2 hours. This time frame overlaps perfectly with the empirical phylotypic stage observed through the embryo development. The correlation between modules reduces after the phylotypic stage.

D.3.3 Significance & Discussion

In this paper we attempted to answer a number of questions regarding the gene expression behavior before, during and after the phylotypic stage from the point of view of gene functional pairing within a species. For instance, how the genes are regulated during the phylotypic stage? Is the conserved transcriptome the cause or consequence of the phylotypic stage? Why the genes change from their "usual" dynamics pattern and show an "unusual" gene expression stability? Our aim is to understand how and why genes change their expression dynamics during embryonic development (Fig. 1, Fig. ED6, Fig. S3, and Suppl. D.3).

The analysis of worm and fly suggests a very strong selective pressure to preserve the "hourglass" pattern. Unlike previous studies focused on making cross-species comparison, our work highlights that the selective pressure could be detected within a species. We propose that these ancestral genes have specialized and different expression patterns both before and after phylotypic stage. Also, during the phylotypic stage, all these ancestral genes are coordinated into a single functional module. This suggests that they are under coordinated regulation, which may relate the gene regulatory network (GRN) kernels found in [116]. In other words, selective pressure is applied to the relationships between genes.

Furthermore, we suggest the following hypothesis: before and after the phylotypic stage, ancestral genes are subject to species-specific regulations. This would allow ancestral genes to take on new roles; thus they are coupled with species-specific genes and present weaker expression correlations among themselves.

D.4. Stage Alignment during Developmental Time-Course

To match the developmental stages of fly and worm (Fig. ED7), we first estimated the expression levels of orthologous genes between fly and worm at different developmental stages by applying Cufflinks [117] to modENCODE timecourse RNA-Seq data. We next identified stage-associated orthologous genes — genes highly expressed at that stage (z-score>1.5) but not always highly expressed across all stages — for every fly and worm developmental stage. Then for every possible pair of fly and worm stages, we counted the number of orthologous gene pairs between worm-stage-associated genes and fly-stage-associated-genes, which would be used to test against the null hypothesis that the fly and worm stages have independent stage-associated genes using a hypergeometric test. Bonferroni correction was used to correct the resultant p-values to decide which fly and worm stages "match" (have dependent stage-associated genes). In the stage mapping of hourglass genes, for each pair of fly and worm stages, we counted the number of orthologous pairs between worm-stage-associated hourglass genes and fly-stage-associated hourglass genes. The subsequent hypergeometric test was performed using hourglass orthologs as background.

We show how hourglass orthologs, non-hourglass orthologs, and orthologs in the 16 modules are aligned in the stage mapping (Fig. S3). As a null model, random collections of genes were used to perform the same alignment procedure. We found hourglass genes show a much stronger alignment than the null models.

E. More Details on "Modeling Gene Expression with Chromatin & TFs"

E.1. Context for "Modeling Gene Expression with Chromatin & TFs"

Previous studies have studied the conservation of histone marks in two aspects: (1) their function as active or repressive markers are conserved [118, 119]; and (2) the DNA region (promoter or enhancer, etc) in which they are functional is conserved [119, 120]. But none of these studies applied a quantitative model to examine the effects of their conservation on the regulation of gene expression. In addition, each histone mark is investigated independently and the effect on gene expression has not been examined collectively. To our knowledge, our analysis quantitatively examined the impact of the conservation of multiple histone markers on gene expression in the same model for the first time.

A number of papers have been published to investigate the relationship between gene expression levels and TF binding or histone modification signals using quantitative models: [2, 118, 120, 121, 122, 123, 124, 125]. Models in these studies quantify the relationship between gene expression and histone modification signals. However, models are constructed in a single species and the conservation of histone marks was not compared in a quantitative manner.

For the first time we compared the TF model and HM model in three organisms. We demonstrate different patterns of predictive powers achieved by TF and HM signals – TF model achieve high predictive accuracy in narrow DNA regions close to TSS, while the HM model achieve fairly high accuracy in a much broader DNA region. In addition, it is novel to apply the TF model to predict non-coding gene expression and compared the predictive its capacity in the three organisms.

E.2. Relating Pol II Binding and H3K4me3 with Gene Expression

In order to compare the levels of Pol II binding proximal to protein-coding genes (around TSS) with the level of gene expression in different organisms, we used the early embryo Pol II ChIP-Seq and matching poly(A)+ RNA-Seq data for both worm and fly, while we used the H1esc (H1 embryo stem cell line) Pol II ChIP-Seq and poly(A)+ RNA-Seq data for human from the ENCODE project [1]. We also investigated the levels of H3K4me3 (methylation of histone H3 lysine 4) proximal to protein-coding genes with its expression in different species. Histone modification mark ChIP-Seq data came from embryo stages.

The most obvious and direct correlation to investigate is the rank correlation between gene expression and Pol II binding or H3K4me3 mark levels close to the gene. Using the matched embryo data sets, we directly plotted the level of Pol II binding against gene expression within each organism (Fig. S4). This shows substantial correlation (r=0.67 in human, 0.62 in fly, and 0.64 in worm). Similarly, we also found substantial correlation (r=0.43 in human, 0.77 in fly, and 0.58 in worm) between the level of H3K4me3 marks close to a gene's TSS and its expression.

E.3. Predictive Models for Gene Expression

E.3.1. Data Preprocessing

To compare the code for transcriptional regulation in human, fly and worm, we constructed predictive models to relate histone modification or TF binding with gene expression in each organism. Specifically, we focused on data from H1ESC cell line for human, and data from early embryo cells for fly and worm. In human, we used GENCODE TSS expression data measured by CAGE experiments in H1ESC cell line. The data contain expression profiles for RNA samples extracted from six different cellular components (whole-cell, cytosolic, nuclear, chromatin, nucleoplasm and nucleolus) using four different protocols (poly(A)+, poly(A)-, total, and short RNA). In our model, we choose the profile that is most correlated with H1ESC RNA Pol II binding data: i.e., the data corresponding to poly(A)+ cytosolic RNA sample. In fly and worm, we used the transcript expression data in early embryo cells measured using RNA-Seq

experiments. For both species, expression profiles at multiple time points are measured and we chose profiles that are most highly correlated with embryo RNA Pol II binding (Fig. S4). That is, we chose RNA-Seq data for fly embryonic stage at the 6 - 8 hour time point and poly(A)+ RNA-Seq data for worm N2 early embryo, respectively. Expression levels are normalized and represented as RPM (reads per million) for human CAGE data, and as RPKM (reads per kilobase per million) for RNA-Seq data. The genome-wide TF-binding and histone modification data are obtained from ChIP-seq or ChIP-chip experiments. We only included data from the matched cell line (H1ESC for human) or development stage (early embryo cells for fly and worm). Only the sequence-specific TFs are used in our models.

The histone modification and TF binding data are processed in the following ways. We separated the DNA regions around the TSS (4kb centering at TSS) of each annotated gene into small bins, each of 100 bp in size, which results in 40 bins for each gene. To calculate the signal of TF binding or histone modifications (HMs), we averaged the coverage (number of reads that cover a nucleotide) of the 100 nt in each of the 40 bins.

E.3.2. Details on the Models

Then, we constructed Random Forest (regression) models to predict gene expression levels based upon the amount of histone modification and TF binding proximal to its TSS, respectively [119, 122]. In the TF model, we used the binding signal of the TSS-containing bin of TFs as predictors, since most TFs have highest correlation in their signal with gene expression levels [121, 124]. However, for different histone modifications (HMs), the gene expression levels are most correlated with the amount of histone modification in different bins relative to the TSS. Thus, we used signals from the most correlated bins of HMs as the predictors in our HM model [118, 120, 126].

To evaluate the performance of each model, we randomly selected 2000 genes as the training data and the remaining genes are used as test data. The models were trained on the training data and applied to predicting the expression levels of genes in the test data. The test data was used to determine the most correlated bins. The predictive accuracy of the models was measured using the Pearson correlation coefficient between the predicted values and the actual experimental expression levels. For each model, we generated 10 groups of training and test data, and their averaged correlation was used as the final predictive accuracy.

For each of the three organisms, we constructed a TF model and HM model. The models are applied to predict the expression levels of protein-coding genes as well as ncRNAs. Separate models were used for three different prediction scenarios - protein-coding (training) to protein-coding (test) genes, protein-coding genes (training) to ncRNAs (test), and ncRNAs (training) to ncRNAs (test), respectively. The R package “randomForest” [127] is used to implement these models. The relative importance of each predictor is measured as %IncMSE, the increase of mean squared error. To calculate it, the weights of each predictor of the test data are permuted and the prediction error (mean squared error of all genes) in the test data is recalculated using the original model. The increase of prediction error is used to measure the relative importance of predictors in a random Forest model [127]. A predictor with higher IncMSE value is more informative for predicting gene expression level.

In the TF model, we notice that very few TFs can be used to predict the gene expression levels of different genes. In particular, while there are ~1400 total TFs in human, ~900 in worm, and ~750 in fly [128, 129, 130, 131], most models with as few as 5 TFs make successful predictions (Fig. ED9 and Fig. S4). However, the TF models do not perform as well in predictions of ncRNA expression levels. The histone models performed more similarly on predicting the gene expression levels of protein-coding genes and ncRNAs than the TF-models – understandable, given the greater dependence of the TF model on the exact positioning of the TSS and the more precise TSS annotation for protein-coding genes compared to ncRNAs. Indeed, the primary transcript for many ncRNAs such as miRNAs is not annotated at all. In addition, in some cases, certain TFs specifically regulate non-coding RNAs and have a large effect on ncRNA expression (e.g. analysis of the targets of GEI-11 [94] show that it mostly binds near ncRNAs.)

E.3.3. An Organism-Independent Universal Model

While the organism-specific models gave excellent gene expression predictions for genes within each organism, these models did not perform as well in predicting gene expression levels for other organisms. Hence, we constructed a universal model based upon the level of seven histone modifications (H3K4me1, H3K4me2, H3K4me3, H4K20me1, H3K27me3, H3K36me3 and H3K27ac) shared by human, fly and worm. To make the expression levels of genes comparable among different organisms, we normalize the log-transformed expression levels of all genes by the median in the corresponding organism. We normalize the histone modification as follows:

$$S_{ij}^{k} = \frac{R_{ij}^{k} - \min(R_{i}^{k})}{\max(R_{i}^{k}) - \min(R_{i}^{k})}$$

where R_{ij}^{k} and S_{ij}^{k} are respectively the original and normalized values of the i-th histone modification for gene j in organism k; $\min(R_{i}^{k})$ and $\max(R_{i}^{k})$ are respectively the minimum and the maximum values of the i-th histone modification in organism k.

The universal model is trained by a set of data containing equal number of genes from the three organisms, and then applied to predict expression levels in different organisms separately. If a common histone code were used in all three organisms, we would expect that the accuracy of the universal model would be similar to the prediction accuracy of each organism specific model (Fig. ED9 and Fig. S4).

We test the universal model in three scenarios: (1) train the model on protein-coding genes and apply it to predict expression of protein-coding genes; (2) train the model on protein-coding genes and apply it to predict expression of ncRNAs; (3) train the model on ncRNA and apply it to predict expression of ncRNA. In all three scenarios, the model achieves fairly high prediction accuracy (Fig. 4, Fig. ED9, and Fig. S4). There are significant differences in the weights of different histone weights in the universal model when compared to the organism specific HM models. For example, the

universal model down-weights H3K27me3, a repressive mark, consistent with the observation that repressive marks are less consistent in their behavior across the organisms than activating marks [64].

F. Associated Data Files

Files located in the following URL: http://cmptxn.gersteinlab.org

F.1. Associated Data Files for "Comparative ENCODE RNA Resource"

F.1.1. RNA-Seq Data Sets

List of all the RNA-seq data sets of human, worm and fly, produced by the consortium.
`Comparative_Datasets.xlsx`

- Data sets used in cross species comparison, in Excel format.

F.1.2. Protein-Coding Gene Annotation

`gen10_CDS+exons_only_protein-coding_only.gtf.gz`

- Human protein-coding gene annotation, in gtf format, from GENCODE v10.

`AG1201.integrated_transcripts_strictly_coding.ws220.gtf.gz`

- Worm protein-coding gene annotation, in gtf format, from modENCODE June 2012 freeze.

`coding_Celniker_Drosophila_Annotation_20120616_1428.gtf.gz`

- Fly protein-coding gene annotation, in gtf format, from modENCODE June 2012 freeze.

F.1.3. Fly Strict Non-coding Gene

`strict_noncoding_Celniker_Drosophila_Annotation_20120616_1428.gtf.gz`

- Fly strict non-coding annotation, in gtf format, from modENCODE June 2012 freeze.

F.1.4. Comparable and Non-comparable Non-coding RNA Annotations

The compressed GFF files for non-coding RNA gene annotations. For each species, there is one compressed file that contains the comparable (miRNA, tRNA, snoRNA, snRNA, pri-miRNA) and one non-comparable ncRNA annotations. The comparable annotations are further separated into the biotypes.
`human_consensus_ncRNAs_03_23_2013.gtf.tar.bz2`

- Human comparable ncRNA, in gtf format.

`human_non_consensus_ncRNAs_03_23_2013.gtf.tar.bz2`

- Human non- comparable ncRNA, in gtf format.

worm_consensus_ncRNAs_03_23_2013.gtf.tar.bz2

- Worm comparable ncRNA, in gtf format.

worm_non_consensus_ncRNAs_03_23_2013.gtf.tar.bz2

- Worm non- comparable ncRNA, in gtf format.

fly_consensus_ncRNAs_03_23_2013.gtf.tar.bz2

- Fly comparable ncRNA, in gtf format.

fly_non_consensus_ncRNAs_03_23_2013.gtf.tar.bz2

- Fly non- comparable ncRNA, in gtf format.

F.1.5. Human-Worm-Fly Ortholog Lists

Modencode.merged.orth20120611_wfh_comm_all.csv

- eMIT Human-Worm-Fly Orthologs.

F.1.6. Table Summarizing All Annotations and Processed Values for Every Gene

These tables provide a summary of all annotations and processed values associated to coding genes in human, worm, and fly. This includes gene expression levels, TF prediction power and orthology etc. Details on the values and features are provided in excel sheet headers.

human_gene.xlsx

- Human coding gene details, in Excel format.

worm_gene.xlsx

- Worm coding gene details, in Excel format.

fly_gene.xlsx

- Fly coding gene details, in Excel format.

F.2. Associated Data Files for "ncRNAs & Non-Canonical Transcription"

F.2.1. TARs

Listing of all the TARs locations in the genome, using the chromosome, start and stop.

human_exon_disc_90_tars.bed

- TARS in human at 90% threshold, in bed format.

human_exon_disc_98_tars.bed

- TARS in human at 98% threshold, in bed format.

worm_exon_disc_90_tars.bed

- TARS in worm at 90% threshold, in bed format.

worm_exon_disc_98_tars.bed

- TARS in worm at 98% threshold, in bed format.

fly_exon_disc_90_tars.bed

- TARS in fly at 90% threshold, in bed format.

fly_exon_disc_98_tars.bed

- TARS in fly at 98% threshold, in bed format.

F.2.2 Clustering of ncRNAs and TARs with Modules

16_module_ncRNA.tar.gz

- ncRNAs and TARs associated with the 16 modules in three species, tarball of txt files.

F.2.3. Supervised ncRNA predictions (novel ncRNA fragments)

The novel ncRNA candidates from the supervised ncRNA predictions.

hg_incRNA_tar98_intersection_50_6Feb13.bed

- Human supervised ncRNA predictions Feb 6 , 2013, in bed format.

ce_incRNA_tar98_intersection_50_6Feb13.bed

- Worm supervised ncRNA predictions Feb 6 , 2013, in bed format.

dm_incRNA_tar98_intersection_50_6Feb13.bed

- Fly supervised ncRNA predictions Feb 6 , 2013, in bed format.

F.3. Associated Data Files for "Expression Clustering & Stage Alignment"

F.3.1. Gene Co-expression Modules

Genes and associated ncRNAs and TARs in related coexpression modules.

16_module.tar.gz

- 16 human, worm and fly co-expressed and co-evolved modules showing highly coordinated expression patterns only during phylotypic stage, tarball of csv files.

genelist_16modules.xlsx

- gene names in 16 conserved modules

GO_16modules_biological_process.xlsx

- Enriched Gene Ontology terms on biological process in 16 conserved modules

GO_16modules_cellular_component.xlsx

- Enriched Gene Ontology terms on cellular component in 16 conserved modules

GO_16modules_molecular_function.xlsx

- Enriched Gene Ontology terms on molecular function in 16 conserved modules

F.3.2. Stage Mapping between Worm and Fly

wf_dual_mapping.xls

- Embryonic specific worm genes aligned with fly genes in both embryo stage and pupae stage.

F.4. Associated Data Files for "Modeling Gene Expression with Chromatin & TFs"

The associated excel files are described in section F.1.6. All Features and Processed Values of Genes.

G. Online data

G.1. modENCODE.org & encodeproject.org

The modENCODE project website, www.modencode.org, and the www.encodeproject.org are the primary entry points for accessing and downloading the entire modENCODE and ENCODE data corpus. The human data is also available is from the RNA dashboard http://genome.crg.cat/encode_RNA_dashboard.

Following the modMine link from the modencode.org provides a searchable interface and easy to explore organization of the data sets. For access to a graphical depiction of the data sets across the chromosomes, follow the "Browse worm Genomes" link to open a GBrowser window The GBrowser enables side by side visual comparison of data sets and provides options to customize, share and export regions of interest. Following the "Experiment Matrix" link from the encodeproject.org describes all the experiments performed for each cell line. The "Search" and "Genome Browser" links allow examination of specific transcriptions at various scales.

The raw data specifically included in this paper is indexed from http://cmptxn.gersteinlab.org/rawdata.

G.2. WormBase, FlyBase, SRA and Beyond

Finally, modENCODE and ENCODE data and analyses are available through many international repositories in various forms. The primary site to access and download the RNA-Seq sequencing data are available from the GEO and SRA resources. The accession numbers for GEO and SRA data sets can be found linked in Comparative_Datasets.xlsx. Worm and Fly interpreted data, including corrected gene models, alternative transcripts, and ChIP peaks, are being incorporated into WormBase and FlyBase.

References

1. ENCODE Project Consortium, Dunham, I., Kundaje, A., Aldred, S. F., Collins, P. J., Davis, C. A., Doyle, F., Epstein, C. B., Frietze, S., Harrow, J., Kaul, R., Khatun, J., Lajoie, B. R., Landt, S. G., Lee, B.-K., Pauli, F., Rosenbloom, K. R., Sabo, P., Safi, A., Sanyal, A., Shoresh, N., Simon, J. M., Song, L., Trinklein, N. D., Altshuler, R. C., Birney, E., Brown, J. B., Cheng, C., Djebali, S., Dong, X., Dunham, I., Ernst, J., Furey, T. S., Gerstein, M., Giardine, B., Greven, M., Hardison, R. C., Harris, R. S., Herrero, J., Hoffman, M. M., Iyer, S., Kelllis, M., Khatun, J., Kheradpour, P., Kundaje, A., Lassman, T., Li, Q., Lin, X., Marinov, G. K., Merkel, A., Mortazavi, A., Parker, S. C. J., Reddy, T. E., Rozowsky, J., Schlesinger, F., Thurman, R. E., Wang, J., Ward, L. D., Whitfield, T. W., Wilder, S. P., Wu, W., Xi, H. S., Yip, K. Y., Zhuang, J., Bernstein, B. E., Birney, E., Dunham, I., Green, E. D., Gunter, C., Snyder, M., Pazin, M. J., Lowdon, R. F., Dillon, L. A. L., Adams, L. B., Kelly, C. J., Zhang, J., Wexler, J. R., Green, E. D., Good, P. J., Feingold, E. A., Bernstein, B. E., Birney, E., Crawford, G. E., Dekker, J., Elinitski, L., Farnham, P. J., Gerstein, M., Giddings, M. C., Gingeras, T. R., Green, E. D., Guigó, R., Hardison, R. C., Hubbard, T. J., Kellis, M., Kent, W. J., Lieb, J. D., Margulies, E. H., Myers, R. M., Snyder, M., Starnatoyannopoulos, J. A., Tennebaum, S. A., Weng, Z., White, K. P., Wold, B., Khatun, J., Yu, Y., Wrobel, J., Risk, B. A., Gunawardena, H. P., Kuiper, H. C., Maier, C. W., Xie, L., Chen, X., Giddings, M. C., Bernstein, B. E., Epstein, C. B., Shoresh, N., Ernst, J., Kheradpour, P., Mikkelsen, T. S., Gillespie, S., Goren, A., Ram, O., Zhang, X., Wang, L., Issner, R., Coyne, M. J., Durham, T., Ku, M., Truong, T., Ward, L. D., Altshuler, R. C., Eaton, M. L., Kellis, M., Djebali, S., Davis, C. A., Merkel, A., Dobin, A., Lassmann, T., Mortazavi, A., Tanzer, A., Lagarde, J., Lin, W., Schlesinger, F., Xue, C., Marinov, G. K., Khatun, J., Williams, B. A., Zaleski, C., Rozowsky, J., Röder, M., Kokocinski, F., Abdelhamid, R. F., Alioto, T., Antoshechkin, I., Baer, M. T., Batut, P., Bell, I., Bell, K., Chakrabortty, S., Chen, X., Chrast, J., Curado, J., Derrien, T., Drenkow, J., Dumais, E., Dumais, J., Duttagupta, R., Fastuca, M., Fejes-Toth, K., Ferreira, P., Foissac, S., Fullwood, M. J., Gao, H., Gonzalez, D., Gordon, A., Gunawardena, H. P., Howald, C., Jha, S., Johnson, R., Kapranov, P., King, B., Kingswood, C., Li, G., Luo, O. J., Park, E., Preall, J. B., Presaud, K., Ribeca, P., Risk, B. A., Robyr, D., Ruan, X., Sammeth, M., Sandu, K. S., Schaeffer, L., See, L.-H., Shahab, A., Skancke, J., Suzuki, A. M., Takahashi, H., Tilgner, H., Trout, D., Walters, N., Wang, H., Wrobel, J., Yu, Y., Hayashizaki, Y., Harrow, J., Gerstein, M., Hubbard, T. J., Reymond, A., Antonarakis, S. E., Hannon, G. J., Giddings, M. C., Ruan, Y., Wold, B., Carninci, P., Guigó, R., Gingeras, T. R., Rosenbloom, K. R., Sloan, C. A., Learned, K., Malladi, V. S., Wong, M. C., Barber, G. P., Cline, M. S., Dreszer, T. R., Heitner, S. G., Karolchik, D., Kent, W. J., Kirkup, V. M., Meyer, L. R., Long, J. C., Maddren, M., Raney, B. J., Furey, T. S., Song, L., Grasfeder, L. L., Giresi, P. G., Lee, B.-K., Battenhouse, A., Sheffield, N. C., Simon, J. M., Showers, K. A., Safi, A., London, D., Bhinge, A. A., Shestak, C., Schaner, M. R., Kim, S. K., Zhang, Z. Z., Mieczkowski, P. A.,

Mieczkowska, J. O., Liu, Z., McDaniell, R. M., Ni, Y., Rashid, N. U., Kim, M. J., Adar, S., Zhang, Z., Wang, T., Winter, D., Keefe, D., Birney, E., Iyer, V. R., Lieb, J. D., Crawford, G. E., Li, G., Sandhu, K. S., Zheng, M., Wang, P., Luo, O. J., Shahab, A., Fullwood, M. J., Ruan, X., Ruan, Y., Myers, R. M., Pauli, F., Williams, B. A., Gertz, J., Marinov, G. K., Reddy, T. E., Vielmetter, J., Partridge, E. C., Trout, D., Varley, K. E., Gasper, C., Bansal, A., Pepke, S., Jain, P., Amrhein, H., Bowling, K. M., Anaya, M., Cross, M. K., King, B., Muratet, M. A., Antoshechkin, I., Newberry, K. M., McCue, K., Nesmith, A. S., Fisher-Aylor, K. I., Pusey, B., DeSalvo, G., Parker, S. L., Balasubramanian, S., Davis, N. S., Meadows, S. K., Eggleston, T., Gunter, C., Newberry, J. S., Levy, S. E., Absher, D. M., Mortazavi, A., Wong, W. H., Wold, B., Blow, M. J., Visel, A., Pennachio, L. A., Elnitski, L., Margulies, E. H., Parker, S. C. J., Petrykowska, H. M., Abyzov, A., Aken, B., Barrell, D., Barson, G., Berry, A., Bignell, A., Boychenko, V., Bussotti, G., Chrast, J., Davidson, C., Derrien, T., Despacio-Reyes, G., Diekhans, M., Ezkurdia, I., Frankish, A., Gilbert, J., Gonzalez, J. M., Griffiths, E., Harte, R., Hendrix, D. A., Howald, C., Hunt, T., Jungreis, I., Kay, M., Khurana, E., Kokocinski, F., Leng, J., Lin, M. F., Loveland, J., Lu, Z., Manthravadi, D., Mariotti, M., Mudge, J., Mukherjee, G., Notredame, C., Pei, B., Rodriguez, J. M., Saunders, G., Sboner, A., Searle, S., Sisu, C., Snow, C., Steward, C., Tanzer, A., Tapanan, E., Tress, M. L., van Baren, M. J., Walters, N., Washieti, S., Wilming, L., Zadissa, A., Zhengdong, Z., Brent, M., Haussler, D., Kellis, M., Valencia, A., Gerstein, M., Raymond, A., Guigó, R., Harrow, J., Hubbard, T. J., Landt, S. G., Frietze, S., Abyzov, A., Addleman, N., Alexander, R. P., Auerbach, R. K., Balasubramanian, S., Bettinger, K., Bhardwaj, N., Boyle, A. P., Cao, A. R., Cayting, P., Charos, A., Cheng, Y., Cheng, C., Eastman, C., Euskirchen, G., Fleming, J. D., Grubert, F., Habegger, L., Hariharan, M., Harmanci, A., Iyenger, S., Jin, V. X., Karczewski, K. J., Kasowski, M., Lacroute, P., Lam, H., Larnarre-Vincent, N., Leng, J., Lian, J., Lindahl-Allen, M., Min, R., Miotto, B., Monahan, H., Moqtaderi, Z., Mu, X. J., O'Geen, H., Ouyang, Z., Patacsil, D., Pei, B., Raha, D., Ramirez, L., Reed, B., Rozowsky, J., Sboner, A., Shi, M., Sisu, C., Slifer, T., Witt, H., Wu, L., Xu, X., Yan, K.-K., Yang, X., Yip, K. Y., Zhang, Z., Struhl, K., Weissman, S. M., Gerstein, M., Farnham, P. J., Snyder, M., Tenebaum, S. A., Penalva, L. O., Doyle, F., Karmakar, S., Landt, S. G., Bhanvadia, R. R., Choudhury, A., Domanus, M., Ma, L., Moran, J., Patacsil, D., Slifer, T., Victorsen, A., Yang, X., Snyder, M., White, K. P., Auer, T., Centarin, L., Eichenlaub, M., Gruhl, F., Heerman, S., Hoeckendorf, B., Inoue, D., Kellner, T., Kirchmaier, S., Mueller, C., Reinhardt, R., Schertel, L., Schneider, S., Sinn, R., Wittbrodt, B., Wittbrodt, J., Weng, Z., Whitfield, T. W., Wang, J., Collins, P. J., Aldred, S. F., Trinklein, N. D., Partridge, E. C., Myers, R. M., Dekker, J., Jain, G., Lajoie, B. R., Sanyal, A., Balasundaram, G., Bates, D. L., Byron, R., Canfield, T. K., Diegel, M. J., Dunn, D., Ebersol, A. K., Ebersol, A. K., Frum, T., Garg, K., Gist, E., Hansen, R. S., Boatman, L., Haugen, E., Humbert, R., Jain, G., Johnson, A. K., Johnson, E. M., Kutyavin, T. M., Lajoie, B. R., Lee, K., Lotakis, D., Maurano, M. T., Neph, S. J., Neri, F. V., Nguyen, E. D., Qu, H., Reynolds, A. P., Roach, V., Rynes, E., Sabo, P., Sanchez, M. E., Sandstrom, R. S., Sanyal, A., Shafer, A. O., Stergachis, A. B., Thomas, S., Thurman, R. E., Vernot, B., Vierstra, J., Vong, S., Wang, H., Weaver, M. A., Yan, Y., Zhang, M., Akey, J. A., Bender, M., Dorschner,

M. O., Groudine, M., MacCoss, M. J., Navas, P., Stamatoyannopoulos, G., Kaul, R., Dekker, J., Stamatoyannopoulos, J. A., Dunham, I., Beal, K., Brazma, A., Flicek, P., Herrero, J., Johnson, N., Keefe, D., Lukk, M., Luscombe, N. M., Sobral, D., Vaquerizas, J. M., Wilder, S. P., Batzoglou, S., Sidow, A., Hussami, N., Kyriazopoulou-Panagiotopoulou, S., Libbrecht, M. W., Schaub, M. A., Kundaje, A., Hardison, R. C., Miller, W., Giardine, B., Harris, R. S., Wu, W., Bickel, P. J., Banfai, B., Boley, N. P., Brown, J. B., Huang, H., Li, Q., Li, J. J., Noble, W. S., Bilmes, J. A., Buske, O. J., Hoffman, M. M., Sahu, A. O., Kharchenko, P. V., Park, P. J., Baker, D., Taylor, J., Weng, Z., Iyer, S., Dong, X., Greven, M., Lin, X., Wang, J., Xi, H. S., Zhuang, J., Gerstein, M., Alexander, R. P., Balasubramanian, S., Cheng, C., Harmanci, A., Lochovsky, L., Min, R., Mu, X. J., Rozowsky, J., Yan, K.-K., Yip, K. Y., & Birney, E. An integrated encyclopedia of DNA elements in the human genome. *Nature* 489, 57-74, (2012)

2. Gerstein, M. B., Lu, Z. J., Van Nostrand, E. L., Cheng, C., Arshinoff, B. I., Liu, T., Yip, K. Y., Robilotto, R., Rechtsteiner, A., Ikegami, K., Alves, P., Chateigner, A., Perry, M., Morris, M., Auerbach, R. K., Feng, X., Leng, J., Vielle, A., Niu, W., Rhrissorrakrai, K., Agarwal, A., Alexander, R. P., Barber, G., Brdlik, C. M., Brennan, J., Brouillet, J. J., Carr, A., Cheung, M.-S., Clawson, H., Contrino, S., Dannenberg, L. O., Dernburg, A. F., Desai, A., Dick, L., Dose, A. C., Du, J., Egelhofer, T., Ercan, S., Euskirchen, G., Ewing, B., Feingold, E. A., Gassmann, R., Good, P. J., Green, P., Gullier, F., Gutwein, M., Guyer, M. S., Habegger, L., Han, T., Henikoff, J. G., Henz, S. R., Hinrichs, A., Holster, H., Hyman, T., Iniguez, A. L., Janette, J., Jensen, M., Kato, M., Kent, W. J., Kephart, E., Khivansara, V., Khurana, E., Kim, J. K., Kolasinska-Zwierz, P., Lai, E. C., Latorre, I., Leahey, A., Lewis, S., Lloyd, P., Lochovsky, L., Lowdon, R. F., Lubling, Y., Lyne, R., MacCoss, M., Mackowiak, S. D., Mangone, M., McKay, S., Mecenas, D., Merrihew, G., Miller, D. M. r., Muroyama, A., Murray, J. I., Ooi, S.-L., Pham, H., Phippen, T., Preston, E. A., Rajewsky, N., Ratsch, G., Rosenbaum, H., Rozowsky, J., Rutherford, K., Ruzanov, P., Sarov, M., Sasidharan, R., Sboner, A., Scheid, P., Segal, E., Shin, H., Shou, C., Slack, F. J., Slightam, C., Smith, R., Spencer, W. C., Stinson, E. O., Taing, S., Takasaki, T., Vafeados, D., Voronina, K., Wang, G., Washington, N. L., Whittle, C. M., Wu, B., Yan, K.-K., Zeller, G., Zha, Z., Zhong, M., Zhou, X., Ahringer, J., Strome, S., Gunsalus, K. C., Micklem, G., Liu, X. S., Reinke, V., Kim, S. K., Hillier, L. W., Henikoff, S., Piano, F., Snyder, M., Stein, L., Lieb, J. D., & Waterston, R. H. Integrative analysis of the Caenorhabditis elegans genome by the modENCODE project.. *Science* 330, 1775--1787, (2010)

3. Graveley, B. R., Brooks, A. N., Carlson, J. W., Duff, M. O., Landolin, J. M., Yang, L., Artieri, C. G., van Baren, M. J., Boley, N., Booth, B. W., Brown, J. B., Cherbas, L., Davis, C. A., Dobin, A., Li, R., Lin, W., Malone, J. H., Mattiuzzo, N. R., Miller, D., Sturgill, D., Tuch, B. B., Zaleski, C., Zhang, D., Blanchette, M., Dudoit, S., Eads, B., Green, R. E., Hammonds, A., Jiang, L., Kapranov, P., Langton, L., Perrimon, N., Sandler, J. E., Wan, K. H., Willingham, A., Zhang, Y., Zou, Y., Andrews, J., Bickel, P. J., Brenner, S. E., Brent, M. R., Cherbas, P., Gingeras, T. R., Hoskins, R. A., Kaufman, T. C., Oliver, B., & Celniker, S. E. The developmental transcriptome of Drosophila melanogaster. *Nature* 471, 473-9, (2011)

4. Brawand, D., Soumillon, M., Necsulea, A., Julien, P., Csárdi, G., Harrigan, P., Weier, M.,

Liechti, A., Aximu-Petri, A., Kircher, M., Albert, F. W., Zeller, U., Khaitovich, P., Grützner, F., Bergmann, S., Nielsen, R., Pääbo, S., & Kaessmann, H. The evolution of gene expression levels in mammalian organs. *Nature* 478, 343-8, (2011)
5. Meunier, J., Lemoine, F., Soumillon, M., Liechti, A., Weier, M., Guschanski, K., Hu, H., Khaitovich, P., & Kaessmann, H. Birth and expression evolution of mammalian microRNA genes. *Genome Res* 23, 34-45, (2013)
6. Barbosa-Morais, N. L., Irimia, M., Pan, Q., Xiong, H. Y., Gueroussov, S., Lee, L. J., Slobodeniuc, V., Kutter, C., Watt, S., Colak, R., Kim, T., Misquitta-Ali, C. M., Wilson, M. D., Kim, P. M., Odom, D. T., Frey, B. J., & Blencowe, B. J. The evolutionary landscape of alternative splicing in vertebrate species. *Science* 338, 1587-93, (2012)
7. Merkin, J., Russell, C., Chen, P., & Burge, C. B. Evolutionary dynamics of gene and isoform regulation in Mammalian tissues. *Science* 338, 1593-9, (2012)
8. Xu, A. G., He, L., Li, Z., Xu, Y., Li, M., Fu, X., Yan, Z., Yuan, Y., Menzel, C., Li, N., Somel, M., Hu, H., Chen, W., Pääbo, S., & Khaitovich, P. Intergenic and repeat transcription in human, chimpanzee and macaque brains measured by RNA-Seq. *PLoS Comput Biol* 6, e1000843, (2010)
9. Khaitovich, P., Kelso, J., Franz, H., Visagie, J., Giger, T., Joerchel, S., Petzold, E., Green, R. E., Lachmann, M., & Pääbo, S. Functionality of intergenic transcription: an evolutionary comparison. *PLoS Genet* 2, e171, (2006)
10. Levin, M., Hashimshony, T., Wagner, F., & Yanai, I. Developmental milestones punctuate gene expression in the Caenorhabditis embryo. *Dev Cell* 22, 1101-8, (2012)
11. Kalinka, A. T., Varga, K. M., Gerrard, D. T., Preibisch, S., Corcoran, D. L., Jarrells, J., Ohler, U., Bergman, C. M., & Tomancak, P. Gene expression divergence recapitulates the developmental hourglass model. *Nature* 468, 811-4, (2010)
12. Simola, D. F., Francis, C., Sniegowski, P. D., & Kim, J. Heterochronic evolution reveals modular timing changes in budding yeast transcriptomes. *Genome Biol* 11, R105, (2010)
13. Busby, M. A., Gray, J. M., Costa, A. M., Stewart, C., Stromberg, M. P., Barnett, D., Chuang, J. H., Springer, M., & Marth, G. T. Expression divergence measured by transcriptome sequencing of four yeast species. *BMC Genomics* 12, 635, (2011)
14. McCarroll, S. A., Murphy, C. T., Zou, S., Pletcher, S. D., Chin, C.-S., Jan, Y. N., Kenyon, C., Bargmann, C. I., & Li, H. Comparing genomic expression patterns across species identifies shared transcriptional profile in aging. *Nat Genet* 36, 197-204, (2004)
15. Kodiath, M. F. A new view of the chronic pain client. *Holist Nurs Pract* 6, 41-6, (1991)
16. Djebali, S., Davis, C. A., Merkel, A., Dobin, A., Lassmann, T., Mortazavi, A., Tanzer, A., Lagarde, J., Lin, W., Schlesinger, F., Xue, C., Marinov, G. K., Khatun, J., Williams, B. A., Zaleski, C., Rozowsky, J., Röder, M., Kokocinski, F., Abdelhamid, R. F., Alioto, T., Antoshechkin, I., Baer, M. T., Bar, N. S., Batut, P., Bell, K., Bell, I., Chakrabortty, S., Chen, X., Chrast, J., Curado, J., Derrien, T., Drenkow, J., Dumais, E., Dumais, J., Duttagupta, R., Falconnet, E., Fastuca, M., Fejes-Toth, K., Ferreira, P., Foissac, S., Fullwood, M. J., Gao, H., Gonzalez, D., Gordon, A., Gunawardena, H., Howald, C., Jha, S., Johnson, R., Kapranov, P.,

King, B., Kingswood, C., Luo, O. J., Park, E., Persaud, K., Preall, J. B., Ribeca, P., Risk, B., Robyr, D., Sammeth, M., Schaffer, L., See, L.-H., Shahab, A., Skancke, J., Suzuki, A. M., Takahashi, H., Tilgner, H., Trout, D., Walters, N., Wang, H., Wrobel, J., Yu, Y., Ruan, X., Hayashizaki, Y., Harrow, J., Gerstein, M., Hubbard, T., Reymond, A., Antonarakis, S. E., Hannon, G., Giddings, M. C., Ruan, Y., Wold, B., Carninci, P., Guigó, R., & Gingeras, T. R. Landscape of transcription in human cells. *Nature* 489, 101-8, (2012)

17. Parkhomchuk, D., Borodina, T., Amstislavskiy, V., Banaru, M., Hallen, L., Krobitsch, S., Lehrach, H., & Soldatov, A. Transcriptome analysis by strand-specific sequencing of complementary DNA. *Nucleic Acids Res* 37, e123, (2009)

18. Cherbas, L., Willingham, A., Zhang, D., Yang, L., Zou, Y., Eads, B. D., Carlson, J. W., Landolin, J. M., Kapranov, P., Dumais, J., Samsonova, A., Choi, J.-H., Roberts, J., Davis, C. A., Tang, H., van Baren, M. J., Ghosh, S., Dobin, A., Bell, K., Lin, W., Langton, L., Duff, M. O., Tenney, A. E., Zaleski, C., Brent, M. R., Hoskins, R. A., Kaufman, T. C., Andrews, J., Graveley, B. R., Perrimon, N., Celniker, S. E., Gingeras, T. R., & Cherbas, P. The transcriptional diversity of 25 Drosophila cell lines. *Genome Res* 21, 301-14, (2011)

19. Brown, B., Boley, N., Stoiber, M., Duff, M. O., May, G., Booth, B. W., Sturgill, D., Cherbas, L., Davis, C. A., Hammonds, A., Andrews, J., Bickel, P. J., Cherbas, P., Gingeras, T. R., Hoskins, R. A., Kaufman, T. C., Oliver, B., Graveley, B. R., & Celniker, S. E. Stranded, tissue-specific RNA sequencing in Drosophila melanogaster reveals the complexity of an animal transcriptome. *Nature* , , (2013)

20. Wu, Bansal, A., Rasmussen, M. D., & Kellis, M. Orthology identification and validation across human, mouse, fly, worm, yeast. *TBD* , , (2013)

21. Zheng, D., Frankish, A., Baertsch, R., Kapranov, P., Reymond, A., Choo, S. W., Lu, Y., Denoeud, F., Antonarakis, S. E., Snyder, M., Ruan, Y., Wei, C.-L., Gingeras, T. R., Guigo, R., Harrow, J., & Gerstein, M. B. Pseudogenes in the ENCODE regions: consensus annotation, analysis of transcription, and evolution.. *Genome Res* 17, 839--851, (2007)

22. Zhang, Z., Carriero, N., Zheng, D., Karro, J., Harrison, P. M., & Gerstein, M. PseudoPipe: an automated pseudogene identification pipeline. *Bioinformatics* 22, 1437-9, (2006)

23. Harrow, J., Denoeud, F., Frankish, A., Reymond, A., Chen, C.-K., Chrast, J., Lagarde, J., Gilbert, J. G. R., Storey, R., Swarbreck, D., Rossier, C., Ucla, C., Hubbard, T., Antonarakis, S. E., & Guigo, R. GENCODE: producing a reference annotation for ENCODE. *Genome Biol* 7 Suppl 1, S4.1-9, (2006)

24. Kozomara, A. & Griffiths-Jones, S. miRBase: integrating microRNA annotation and deep-sequencing data. *Nucleic Acids Res* 39, D152-7, (2011)

25. Nam, J.-W. & Bartel, D. P. Long noncoding RNAs in C. elegans. *Genome Res* 22, 2529-40, (2012)

26. Lu, Z. J., Yip, K. Y., Wang, G., Shou, C., Hillier, L. W., Khurana, E., Agarwal, A., Auerbach, R., Rozowsky, J., Cheng, C., Kato, M., Miller, D. M., Slack, F., Snyder, M., Waterston, R. H., Reinke, V., & Gerstein, M. B. Prediction and characterization of noncoding RNAs in C. elegans by integrating conservation, secondary structure, and high-throughput

sequencing and array data. *Genome Res* 21, 276-85, (2011)
27. Czech, B. & Hannon, G. J. Small RNA sorting: matchmaking for Argonautes. *Nat Rev Genet* 12, 19-31, (2011)
28. Ladewig, E., Okamura, K., Flynt, A. S., Westholm, J. O., & Lai, E. C. Discovery of hundreds of mirtrons in mouse and human small RNA data. *Genome Res* 22, 1634-45, (2012)
29. Aboobaker, A. A., Tomancak, P., Patel, N., Rubin, G. M., & Lai, E. C. Drosophila microRNAs exhibit diverse spatial expression patterns during embryonic development. *Proc Natl Acad Sci U S A* 102, 18017-22, (2005)
30. Martinez, N. J., Ow, M. C., Reece-Hoyes, J. S., Barrasa, M. I., Ambros, V. R., & Walhout, A. J. M. Genome-scale spatiotemporal analysis of Caenorhabditis elegans microRNA promoter activity. *Genome Res* 18, 2005-15, (2008)
31. Marson, A., Levine, S. S., Cole, M. F., Frampton, G. M., Brambrink, T., Johnstone, S., Guenther, M. G., Johnston, W. K., Wernig, M., Newman, J., Calabrese, J. M., Dennis, L. M., Volkert, T. L., Gupta, S., Love, J., Hannett, N., Sharp, P. A., Bartel, D. P., Jaenisch, R., & Young, R. A. Connecting microRNA genes to the core transcriptional regulatory circuitry of embryonic stem cells. *Cell* 134, 521-33, (2008)
32. Bender, W. MicroRNAs in the Drosophila bithorax complex. *Genes Dev* 22, 14-9, (2008)
33. Sokol, N. S., Xu, P., Jan, Y.-N., & Ambros, V. Drosophila let-7 microRNA is required for remodeling of the neuromusculature during metamorphosis. *Genes Dev* 22, 1591-6, (2008)
34. Kadener, S., Rodriguez, J., Abruzzi, K. C., Khodor, Y. L., Sugino, K., Marr, 2nd, M. T., Nelson, S., & Rosbash, M. Genome-wide identification of targets of the drosha-pasha/DGCR8 complex. *RNA* 15, 537-45, (2009)
35. Ruby, J. G., Stark, A., Johnston, W. K., Kellis, M., Bartel, D. P., & Lai, E. C. Evolution, biogenesis, expression, and target predictions of a substantially expanded set of Drosophila microRNAs. *Genome Res* 17, 1850-64, (2007)
36. Bracht, J., Hunter, S., Eachus, R., Weeks, P., & Pasquinelli, A. E. Trans-splicing and polyadenylation of let-7 microRNA primary transcripts. *RNA* 10, 1586-94, (2004)
37. Gu, W., Lee, H.-C., Chaves, D., Youngman, E. M., Pazour, G. J., Conte, Jr, D., & Mello, C. C. CapSeq and CIP-TAP Identify Pol II Start Sites and Reveal Capped Small RNAs as C. elegans piRNA Precursors. *Cell* 151, 1488-500, (2012)
38. Lowe, T. M. & Eddy, S. R. tRNAscan-SE: a program for improved detection of transfer RNA genes in genomic sequence. *Nucleic Acids Res* 25, 955-64, (1997)
39. Lestrade, L. & Weber, M. J. snoRNA-LBME-db, a comprehensive database of human H/ACA and C/D box snoRNAs. *Nucleic Acids Res* 34, D158-62, (2006)
40. Higa, S., Maeda, N., Kenmochi, N., & Tanaka, T. Location of 2(')-O-methyl nucleotides in 26S rRNA and methylation guide snoRNAs in Caenorhabditis elegans. *Biochem Biophys Res Commun* 297, 1344-9, (2002)
41. Huang, Z.-P., Zhou, H., He, H.-L., Chen, C.-L., Liang, D., & Qu, L.-H. Genome-wide analyses of two families of snoRNA genes from Drosophila melanogaster, demonstrating the

extensive utilization of introns for coding of snoRNAs. *RNA* 11, 1303-16, (2005)
42. Lindgren, V., Bernstein, L. B., Weiner, A. M., & Francke, U. Human U1 small nuclear RNA pseudogenes do not map to the site of the U1 genes in 1p36 but are clustered in 1q12-q22. *Mol Cell Biol* 5, 2172-80, (1985)
43. Lindgren, V., Ares, Jr, M., Weiner, A. M., & Francke, U. Human genes for U2 small nuclear RNA map to a major adenovirus 12 modification site on chromosome 17. *Nature* 314, 115-6, (1985)
44. Thomas, J., Lea, K., Zucker-Aprison, E., & Blumenthal, T. The spliceosomal snRNAs of Caenorhabditis elegans. *Nucleic Acids Res* 18, 2633-42, (1990)
45. Mount, S. M., Gotea, V., Lin, C.-F., Hernandez, K., & Makalowski, W. Spliceosomal small nuclear RNA genes in 11 insect genomes. *RNA* 13, 5-14, (2007)
46. Financsek, I., Mizumoto, K., Mishima, Y., & Muramatsu, M. Human ribosomal RNA gene: nucleotide sequence of the transcription initiation region and comparison of three mammalian genes. *Proc Natl Acad Sci U S A* 79, 3092-6, (1982)
47. Ellis, R. E., Sulston, J. E., & Coulson, A. R. The rDNA of C. elegans: sequence and structure. *Nucleic Acids Res* 14, 2345-64, (1986)
48. Tautz, D., Hancock, J. M., Webb, D. A., Tautz, C., & Dover, G. A. Complete sequences of the rRNA genes of Drosophila melanogaster. *Mol Biol Evol* 5, 366-76, (1988)
49. Aravin, A., Gaidatzis, D., Pfeffer, S., Lagos-Quintana, M., Landgraf, P., Iovino, N., Morris, P., Brownstein, M. J., Kuramochi-Miyagawa, S., Nakano, T., Chien, M., Russo, J. J., Ju, J., Sheridan, R., Sander, C., Zavolan, M., & Tuschl, T. A novel class of small RNAs bind to MILI protein in mouse testes. *Nature* 442, 203-7, (2006)
50. Girard, A., Sachidanandam, R., Hannon, G. J., & Carmell, M. A. A germline-specific class of small RNAs binds mammalian Piwi proteins. *Nature* 442, 199-202, (2006)
51. Cloonan, N., Wani, S., Xu, Q., Gu, J., Lea, K., Heater, S., Barbacioru, C., Steptoe, A. L., Martin, H. C., Nourbakhsh, E., Krishnan, K., Gardiner, B., Wang, X., Nones, K., Steen, J. A., Matigian, N. A., Wood, D. L., Kassahn, K. S., Waddell, N., Shepherd, J., Lee, C., Ichikawa, J., McKernan, K., Bramlett, K., Kuersten, S., & Grimmond, S. M. MicroRNAs and their isomiRs function cooperatively to target common biological pathways. *Genome Biol* 12, R126, (2011)
52. Robine, N., Lau, N. C., Balla, S., Jin, Z., Okamura, K., Kuramochi-Miyagawa, S., Blower, M. D., & Lai, E. C. A broadly conserved pathway generates 3'UTR-directed primary piRNAs. *Curr Biol* 19, 2066-76, (2009)
53. Malone, C. D., Brennecke, J., Dus, M., Stark, A., McCombie, W. R., Sachidanandam, R., & Hannon, G. J. Specialized piRNA pathways act in germline and somatic tissues of the Drosophila ovary. *Cell* 137, 522-35, (2009)
54. Ruby, J. G., Jan, C., Player, C., Axtell, M. J., Lee, W., Nusbaum, C., Ge, H., & Bartel, D. P. Large-scale sequencing reveals 21U-RNAs and additional microRNAs and endogenous siRNAs in C. elegans. *Cell* 127, 1193-207, (2006)
55. Batista, P. J., Ruby, J. G., Claycomb, J. M., Chiang, R., Fahlgren, N., Kasschau, K. D., Chaves, D. A., Gu, W., Vasale, J. J., Duan, S., Conte, Jr, D., Luo, S., Schroth, G. P., Carrington,

J. C., Bartel, D. P., & Mello, C. C. PRG-1 and 21U-RNAs interact to form the piRNA complex required for fertility in C. elegans. *Mol Cell* 31, 67-78, (2008)
56. Ishizu, H., Siomi, H., & Siomi, M. C. Biology of PIWI-interacting RNAs: new insights into biogenesis and function inside and outside of germlines. *Genes Dev* 26, 2361-73, (2012)
57. Perreault, J., Perreault, J.-P., & Boire, G. Ro-associated Y RNAs in metazoans: evolution and diversification. *Mol Biol Evol* 24, 1678-89, (2007)
58. Lander, E. S., Linton, L. M., Birren, B., Nusbaum, C., Zody, M. C., Baldwin, J., Devon, K., Dewar, K., Doyle, M., FitzHugh, W., Funke, R., Gage, D., Harris, K., Heaford, A., Howland, J., Kann, L., Lehoczky, J., LeVine, R., McEwan, P., McKernan, K., Meldrim, J., Mesirov, J. P., Miranda, C., Morris, W., Naylor, J., Raymond, C., Rosetti, M., Santos, R., Sheridan, A., Sougnez, C., Stange-Thomann, N., Stojanovic, N., Subramanian, A., Wyman, D., Rogers, J., Sulston, J., Ainscough, R., Beck, S., Bentley, D., Burton, J., Clee, C., Carter, N., Coulson, A., Deadman, R., Deloukas, P., Dunham, A., Dunham, I., Durbin, R., French, L., Grafham, D., Gregory, S., Hubbard, T., Humphray, S., Hunt, A., Jones, M., Lloyd, C., McMurray, A., Matthews, L., Mercer, S., Milne, S., Mullikin, J. C., Mungall, A., Plumb, R., Ross, M., Shownkeen, R., Sims, S., Waterston, R. H., Wilson, R. K., Hillier, L. W., McPherson, J. D., Marra, M. A., Mardis, E. R., Fulton, L. A., Chinwalla, A. T., Pepin, K. H., Gish, W. R., Chissoe, S. L., Wendl, M. C., Delehaunty, K. D., Miner, T. L., Delehaunty, A., Kramer, J. B., Cook, L. L., Fulton, R. S., Johnson, D. L., Minx, P. J., Clifton, S. W., Hawkins, T., Branscomb, E., Predki, P., Richardson, P., Wenning, S., Slezak, T., Doggett, N., Cheng, J. F., Olsen, A., Lucas, S., Elkin, C., Uberbacher, E., Frazier, M., Gibbs, R. A., Muzny, D. M., Scherer, S. E., Bouck, J. B., Sodergren, E. J., Worley, K. C., Rives, C. M., Gorrell, J. H., Metzker, M. L., Naylor, S. L., Kucherlapati, R. S., Nelson, D. L., Weinstock, G. M., Sakaki, Y., Fujiyama, A., Hattori, M., Yada, T., Toyoda, A., Itoh, T., Kawagoe, C., Watanabe, H., Totoki, Y., Taylor, T., Weissenbach, J., Heilig, R., Saurin, W., Artiguenave, F., Brottier, P., Bruls, T., Pelletier, E., Robert, C., Wincker, P., Smith, D. R., Doucette-Stamm, L., Rubenfield, M., Weinstock, K., Lee, H. M., Dubois, J., Rosenthal, A., Platzer, M., Nyakatura, G., Taudien, S., Rump, A., Yang, H., Yu, J., Wang, J., Huang, G., Gu, J., Hood, L., Rowen, L., Madan, A., Qin, S., Davis, R. W., Federspiel, N. A., Abola, A. P., Proctor, M. J., Myers, R. M., Schmutz, J., Dickson, M., Grimwood, J., Cox, D. R., Olson, M. V., Kaul, R., Raymond, C., Shimizu, N., Kawasaki, K., Minoshima, S., Evans, G. A., Athanasiou, M., Schultz, R., Roe, B. A., Chen, F., Pan, H., Ramser, J., Lehrach, H., Reinhardt, R., McCombie, W. R., de la Bastide, M., Dedhia, N., Blöcker, H., Hornischer, K., Nordsiek, G., Agarwala, R., Aravind, L., Bailey, J. A., Bateman, A., Batzoglou, S., Birney, E., Bork, P., Brown, D. G., Burge, C. B., Cerutti, L., Chen, H. C., Church, D., Clamp, M., Copley, R. R., Doerks, T., Eddy, S. R., Eichler, E. E., Furey, T. S., Galagan, J., Gilbert, J. G., Harmon, C., Hayashizaki, Y., Haussler, D., Hermjakob, H., Hokamp, K., Jang, W., Johnson, L. S., Jones, T. A., Kasif, S., Kaspryzk, A., Kennedy, S., Kent, W. J., Kitts, P., Koonin, E. V., Korf, I., Kulp, D., Lancet, D., Lowe, T. M., McLysaght, A., Mikkelsen, T., Moran, J. V., Mulder, N., Pollara, V. J., Ponting, C. P., Schuler, G., Schultz, J., Slater, G., Smit, A. F., Stupka, E., Szustakowski, J., Thierry-Mieg, D., Thierry-Mieg, J., Wagner, L., Wallis, J., Wheeler, R., Williams, A., Wolf, Y.

I., Wolfe, K. H., Yang, S. P., Yeh, R. F., Collins, F., Guyer, M. S., Peterson, J., Felsenfeld, A., Wetterstrand, K. A., Patrinos, A., Morgan, M. J., de Jong, P., Catanese, J. J., Osoegawa, K., Shizuya, H., Choi, S., Chen, Y. J., Szustakowki, J., & International Human Genome Sequencing Consortium Initial sequencing and analysis of the human genome. *Nature* 409, 860-921, (2001)

59. Venter, J. C., Adams, M. D., Myers, E. W., Li, P. W., Mural, R. J., Sutton, G. G., Smith, H. O., Yandell, M., Evans, C. A., Holt, R. A., Gocayne, J. D., Amanatides, P., Ballew, R. M., Huson, D. H., Wortman, J. R., Zhang, Q., Kodira, C. D., Zheng, X. H., Chen, L., Skupski, M., Subramanian, G., Thomas, P. D., Zhang, J., Gabor Miklos, G. L., Nelson, C., Broder, S., Clark, A. G., Nadeau, J., McKusick, V. A., Zinder, N., Levine, A. J., Roberts, R. J., Simon, M., Slayman, C., Hunkapiller, M., Bolanos, R., Delcher, A., Dew, I., Fasulo, D., Flanigan, M., Florea, L., Halpern, A., Hannenhalli, S., Kravitz, S., Levy, S., Mobarry, C., Reinert, K., Remington, K., Abu-Threideh, J., Beasley, E., Biddick, K., Bonazzi, V., Brandon, R., Cargill, M., Chandramouliswaran, I., Charlab, R., Chaturvedi, K., Deng, Z., Di Francesco, V., Dunn, P., Eilbeck, K., Evangelista, C., Gabrielian, A. E., Gan, W., Ge, W., Gong, F., Gu, Z., Guan, P., Heiman, T. J., Higgins, M. E., Ji, R. R., Ke, Z., Ketchum, K. A., Lai, Z., Lei, Y., Li, Z., Li, J., Liang, Y., Lin, X., Lu, F., Merkulov, G. V., Milshina, N., Moore, H. M., Naik, A. K., Narayan, V. A., Neelam, B., Nusskern, D., Rusch, D. B., Salzberg, S., Shao, W., Shue, B., Sun, J., Wang, Z., Wang, A., Wang, X., Wang, J., Wei, M., Wides, R., Xiao, C., Yan, C., Yao, A., Ye, J., Zhan, M., Zhang, W., Zhang, H., Zhao, Q., Zheng, L., Zhong, F., Zhong, W., Zhu, S., Zhao, S., Gilbert, D., Baumhueter, S., Spier, G., Carter, C., Cravchik, A., Woodage, T., Ali, F., An, H., Awe, A., Baldwin, D., Baden, H., Barnstead, M., Barrow, I., Beeson, K., Busam, D., Carver, A., Center, A., Cheng, M. L., Curry, L., Danaher, S., Davenport, L., Desilets, R., Dietz, S., Dodson, K., Doup, L., Ferriera, S., Garg, N., Gluecksmann, A., Hart, B., Haynes, J., Haynes, C., Heiner, C., Hladun, S., Hostin, D., Houck, J., Howland, T., Ibegwam, C., Johnson, J., Kalush, F., Kline, L., Koduru, S., Love, A., Mann, F., May, D., McCawley, S., McIntosh, T., McMullen, I., Moy, M., Moy, L., Murphy, B., Nelson, K., Pfannkoch, C., Pratts, E., Puri, V., Qureshi, H., Reardon, M., Rodriguez, R., Rogers, Y. H., Romblad, D., Ruhfel, B., Scott, R., Sitter, C., Smallwood, M., Stewart, E., Strong, R., Suh, E., Thomas, R., Tint, N. N., Tse, S., Vech, C., Wang, G., Wetter, J., Williams, S., Williams, M., Windsor, S., Winn-Deen, E., Wolfe, K., Zaveri, J., Zaveri, K., Abril, J. F., Guigó, R., Campbell, M. J., Sjolander, K. V., Karlak, B., Kejariwal, A., Mi, H., Lazareva, B., Hatton, T., Narechania, A., Diemer, K., Muruganujan, A., Guo, N., Sato, S., Bafna, V., Istrail, S., Lippert, R., Schwartz, R., Walenz, B., Yooseph, S., Allen, D., Basu, A., Baxendale, J., Blick, L., Caminha, M., Carnes-Stine, J., Caulk, P., Chiang, Y. H., Coyne, M., Dahlke, C., Mays, A., Dombroski, M., Donnelly, M., Ely, D., Esparham, S., Fosler, C., Gire, H., Glanowski, S., Glasser, K., Glodek, A., Gorokhov, M., Graham, K., Gropman, B., Harris, M., Heil, J., Henderson, S., Hoover, J., Jennings, D., Jordan, C., Jordan, J., Kasha, J., Kagan, L., Kraft, C., Levitsky, A., Lewis, M., Liu, X., Lopez, J., Ma, D., Majoros, W., McDaniel, J., Murphy, S., Newman, M., Nguyen, T., Nguyen, N., Nodell, M., Pan, S., Peck, J., Peterson, M., Rowe, W., Sanders, R., Scott, J., Simpson, M., Smith, T., Sprague, A., Stockwell, T., Turner, R., Venter, E., Wang, M., Wen, M., Wu, D., Wu, M., Xia, A., Zandieh, A., & Zhu, X. The sequence of the

human genome. *Science* 291, 1304-51, (2001)
60. Punta, M., Coggill, P. C., Eberhardt, R. Y., Mistry, J., Tate, J., Boursnell, C., Pang, N., Forslund, K., Ceric, G., Clements, J., Heger, A., Holm, L., Sonnhammer, E. L. L., Eddy, S. R., Bateman, A., & Finn, R. D. The Pfam protein families database. *Nucleic Acids Res* 40, D290-301, (2012)
61. Robinson, M. D. & Oshlack, A. A scaling normalization method for differential expression analysis of RNA-seq data. *Genome Biol* 11, R25, (2010)
62. Lovén, J., Orlando, D. A., Sigova, A. A., Lin, C. Y., Rahl, P. B., Burge, C. B., Levens, D. L., Lee, T. I., & Young, R. A. Revisiting global gene expression analysis. *Cell* 151, 476-82, (2012)
63. Robinson, M. D., McCarthy, D. J., & Smyth, G. K. edgeR: a Bioconductor package for differential expression analysis of digital gene expression data. *Bioinformatics* 26, 139-40, (2010)
64. Ho, J. W. K., Liu, T., Jung, Y. L., Alver, B. H., Lee, S., Ikegami, K., Sohn, K.-A., Minoda, A., Tolstorukov, M. Y., Appert, A., Parker, S. C. J., Gu, T., Kundaje, A., Riddle, N. C., Bishop, E., Egelhofer, T. A., Hu, S. S., Alekseyenko, A. A., Rechtsteiner, A., Asker, D., Belsky, J. A., Bowman, S. K., Chen, Q. B., Chen, R. A.-J., Day, D. S., Dong, Y., Dosé, A. C., Duan, X., Epstein, C. B., Ercan, S., Feingold, E. A., Garrigues, J. M., Gehlenborg, N., Good, P. J., Haseley, P., He, D., Herrmann, M., Hoffman, M. M., Jeffers, T. E., Kharchenko, P. V., Kolasinska-Zwierz, P., Kotwaliwale, C. V., Kumar, N., Langley, S. A., Larschan, E. N., Latorre, I., Libbrecht, M. W., Lin, X., Park, R., Pazin, M. J., Pham, H. N., Plachetka, A., Qin, B., Schwartz, Y. B., Shoresh, N., Stempor, P., Vielle, A., Wang, C., Whittle, C. M., Xue, H., Kingston, R. E., Kim, J. H., Bernstein, B. E., Dernburg, A. F., Pirrotta, V., Kuroda, M. I., Noble, W. S., Tullius, T. D., Kellis, M., MacAlpine, D. M., Strome, S., Elgin, S. C. R., Ahringer, J., Liu, X. S., and Gary H. Karpen, and Jason D. Lieb, & Park, P. J. modENCODE and ENCODE resources for analysis of metazoan chromatin organization. *Nature* submitted, , (2013)
65. Zhu, L. J., Gazin, C., Lawson, N. D., Pagès, H., Lin, S. M., Lapointe, D. S., & Green, M. R. ChIPpeakAnno: a Bioconductor package to annotate ChIP-seq and ChIP-chip data. *BMC Bioinformatics* 11, 237, (2010)
66. Wang, E. T., Sandberg, R., Luo, S., Khrebtukova, I., Zhang, L., Mayr, C., Kingsmore, S. F., Schroth, G. P., & Burge, C. B. Alternative isoform regulation in human tissue transcriptomes. *Nature* 456, 470-6, (2008)
67. Pan, Q., Shai, O., Lee, L. J., Frey, B. J., & Blencowe, B. J. Deep surveying of alternative splicing complexity in the human transcriptome by high-throughput sequencing. *Nat Genet* 40, 1413-5, (2008)
68. Schmucker, D., Clemens, J. C., Shu, H., Worby, C. A., Xiao, J., Muda, M., Dixon, J. E., & Zipursky, S. L. Drosophila Dscam is an axon guidance receptor exhibiting extraordinary molecular diversity. *Cell* 101, 671-84, (2000)
69. Berget, S. M. Exon recognition in vertebrate splicing. *J Biol Chem* 270, 2411-4, (1995)
70. Talerico, M. & Berget, S. M. Intron definition in splicing of small Drosophila introns.

Mol Cell Biol 14, 3434-45, (1994)
71. Park, J. W. & Graveley, B. R. Complex alternative splicing. *Adv Exp Med Biol* 623, 50-63, (2007)
72. Sievers, F., Wilm, A., Dineen, D., Gibson, T. J., Karplus, K., Li, W., Lopez, R., McWilliam, H., Remmert, M., Söding, J., Thompson, J. D., & Higgins, D. G. Fast, scalable generation of high-quality protein multiple sequence alignments using Clustal Omega. *Mol Syst Biol* 7, 539, (2011)
73. Trapnell, C., Pachter, L., & Salzberg, S. L. TopHat: discovering splice junctions with RNA-Seq. *Bioinformatics* 25, 1105-11, (2009)
74. Li, H., Handsaker, B., Wysoker, A., Fennell, T., Ruan, J., Homer, N., Marth, G., Abecasis, G., Durbin, R., & 1000 Genome Project Data Processing Subgroup The Sequence Alignment/Map format and SAMtools. *Bioinformatics* 25, 2078-9, (2009)
75. Quinlan, A. R. & Hall, I. M. BEDTools: a flexible suite of utilities for comparing genomic features. *Bioinformatics* 26, 841-2, (2010)
76. Katz, Y., Wang, E. T., Airoldi, E. M., & Burge, C. B. Analysis and design of RNA sequencing experiments for identifying isoform regulation. *Nat Methods* 7, 1009-15, (2010)
77. Bertone, P., Stolc, V., Royce, T. E., Rozowsky, J. S., Urban, A. E., Zhu, X., Rinn, J. L., Tongprasit, W., Samanta, M., Weissman, S., Gerstein, M., & Snyder, M. Global identification of human transcribed sequences with genome tiling arrays. *Science* 306, 2242-6, (2004)
78. Cheng, J., Kapranov, P., Drenkow, J., Dike, S., Brubaker, S., Patel, S., Long, J., Stern, D., Tammana, H., Helt, G., Sementchenko, V., Piccolboni, A., Bekiranov, S., Bailey, D. K., Ganesh, M., Ghosh, S., Bell, I., Gerhard, D. S., & Gingeras, T. R. Transcriptional maps of 10 human chromosomes at 5-nucleotide resolution. *Science* 308, 1149-54, (2005)
79. Birney, E., Stamatoyannopoulos, J. A., Dutta, A., Guigo, R., Gingeras, T. R., Margulies, E. H., Weng, Z., Snyder, M., Dermitzakis, E. T., Thurman, R. E., Kuehn, M. S., Taylor, C. M., Neph, S., Koch, C. M., Asthana, S., Malhotra, A., Adzhubei, I., Greenbaum, J. A., Andrews, R. M., Flicek, P., Boyle, P. J., Cao, H., Carter, N. P., Clelland, G. K., Davis, S., Day, N., Dhami, P., Dillon, S. C., Dorschner, M. O., Fiegler, H., Giresi, P. G., Goldy, J., Hawrylycz, M., Haydock, A., Humbert, R., James, K. D., Johnson, B. E., Johnson, E. M., Frum, T. T., Rosenzweig, E. R., Karnani, N., Lee, K., Lefebvre, G. C., Navas, P. A., Neri, F., Parker, S. C. J., Sabo, P. J., Sandstrom, R., Shafer, A., Vetrie, D., Weaver, M., Wilcox, S., Yu, M., Collins, F. S., Dekker, J., Lieb, J. D., Tullius, T. D., Crawford, G. E., Sunyaev, S., Noble, W. S., Dunham, I., Denoeud, F., Reymond, A., Kapranov, P., Rozowsky, J., Zheng, D., Castelo, R., Frankish, A., Harrow, J., Ghosh, S., Sandelin, A., Hofacker, I. L., Baertsch, R., Keefe, D., Dike, S., Cheng, J., Hirsch, H. A., Sekinger, E. A., Lagarde, J., Abril, J. F., Shahab, A., Flamm, C., Fried, C., Hackermuller, J., Hertel, J., Lindemeyer, M., Missal, K., Tanzer, A., Washietl, S., Korbel, J., Emanuelsson, O., Pedersen, J. S., Holroyd, N., Taylor, R., Swarbreck, D., Matthews, N., Dickson, M. C., Thomas, D. J., Weirauch, M. T., Gilbert, J., Drenkow, J., Bell, I., Zhao, X., Srinivasan, K. G., Sung, W.-K., Ooi, H. S., Chiu, K. P., Foissac, S., Alioto, T., Brent, M., Pachter, L., Tress, M. L., Valencia, A., Choo, S. W., Choo, C. Y., Ucla, C., Manzano, C., Wyss, C., Cheung, E., Clark, T. G.,

Brown, J. B., Ganesh, M., Patel, S., Tammana, H., Chrast, J., Henrichsen, C. N., Kai, C., Kawai, J., Nagalakshmi, U., Wu, J., Lian, Z., Lian, J., Newburger, P., Zhang, X., Bickel, P., Mattick, J. S., Carninci, P., Hayashizaki, Y., Weissman, S., Hubbard, T., Myers, R. M., Rogers, J., Stadler, P. F., Lowe, T. M., Wei, C.-L., Ruan, Y., Struhl, K., Gerstein, M., Antonarakis, S. E., Fu, Y., Green, E. D., Karaoz, U., Siepel, A., Taylor, J., Liefer, L. A., Wetterstrand, K. A., Good, P. J., Feingold, E. A., Guyer, M. S., Cooper, G. M., Asimenos, G., Dewey, C. N., Hou, M., Nikolaev, S., Montoya-Burgos, J. I., Loytynoja, A., Whelan, S., Pardi, F., Massingham, T., Huang, H., Zhang, N. R., Holmes, I., Mullikin, J. C., Ureta-Vidal, A., Paten, B., Seringhaus, M., Church, D., Rosenbloom, K., Kent, W. J., Stone, E. A., Batzoglou, S., Goldman, N., Hardison, R. C., Haussler, D., Miller, W., Sidow, A., Trinklein, N. D., Zhang, Z. D., Barrera, L., Stuart, R., King, D. C., Ameur, A., Enroth, S., Bieda, M. C., Kim, J., Bhinge, A. A., Jiang, N., Liu, J., Yao, F., Vega, V. B., Lee, C. W. H., Ng, P., Shahab, A., Yang, A., Moqtaderi, Z., Zhu, Z., Xu, X., Squazzo, S., Oberley, M. J., Inman, D., Singer, M. A., Richmond, T. A., Munn, K. J., Rada-Iglesias, A., Wallerman, O., Komorowski, J., Fowler, J. C., Couttet, P., Bruce, A. W., Dovey, O. M., Ellis, P. D., Langford, C. F., Nix, D. A., Euskirchen, G., Hartman, S., Urban, A. E., Kraus, P., Van Calcar, S., Heintzman, N., Kim, T. H., Wang, K., Qu, C., Hon, G., Luna, R., Glass, C. K., Rosenfeld, M. G., Aldred, S. F., Cooper, S. J., Halees, A., Lin, J. M., Shulha, H. P., Zhang, X., Xu, M., Haidar, J. N. S., Yu, Y., Ruan, Y., Iyer, V. R., Green, R. D., Wadelius, C., Farnham, P. J., Ren, B., Harte, R. A., Hinrichs, A. S., Trumbower, H., Clawson, H., Hillman-Jackson, J., Zweig, A. S., Smith, K., Thakkapallayil, A., Barber, G., Kuhn, R. M., Karolchik, D., Armengol, L., Bird, C. P., de Bakker, P. I. W., Kern, A. D., Lopez-Bigas, N., Martin, J. D., Stranger, B. E., Woodroffe, A., Davydov, E., Dimas, A., Eyras, E., Hallgrimsdottir, I. B., Huppert, J., Zody, M. C., Abecasis, G. R., Estivill, X., Bouffard, G. G., Guan, X., Hansen, N. F., Idol, J. R., Maduro, V. V. B., Maskeri, B., McDowell, J. C., Park, M., Thomas, P. J., Young, A. C., Blakesley, R. W., Muzny, D. M., Sodergren, E., Wheeler, D. A., Worley, K. C., Jiang, H., Weinstock, G. M., Gibbs, R. A., Graves, T., Fulton, R., Mardis, E. R., Wilson, R. K., Clamp, M., Cuff, J., Gnerre, S., Jaffe, D. B., Chang, J. L., Lindblad-Toh, K., Lander, E. S., Koriabine, M., Nefedov, M., Osoegawa, K., Yoshinaga, Y., Zhu, B., & de Jong, P. J. Identification and analysis of functional elements in 1{\%} of the human genome by the ENCODE pilot project.. *Nature* 447, 799--816, (2007)

80. Venters, B. J. & Pugh, B. F. Genomic organization of human transcription initiation complexes. *Nature* 502, 53-58, (2013)

81. Dinger, M. E., Amaral, P. P., Mercer, T. R., & Mattick, J. S. Pervasive transcription of the eukaryotic genome: functional indices and conceptual implications. *Brief Funct Genomic Proteomic* 8, 407-23, (2009)

82. van Bakel, H., Nislow, C., Blencowe, B. J., & Hughes, T. R. Most "dark matter" transcripts are associated with known genes. *PLoS Biol* 8, e1000371, (2010)

83. Clark, M. B., Amaral, P. P., Schlesinger, F. J., Dinger, M. E., Taft, R. J., Rinn, J. L., Ponting, C. P., Stadler, P. F., Morris, K. V., Morillon, A., Rozowsky, J. S., Gerstein, M. B., Wahlestedt, C., Hayashizaki, Y., Carninci, P., Gingeras, T. R., & Mattick, J. S. The reality of

pervasive transcription. *PLoS Biol* 9, e1000625; discussion e1001102, (2011)
84. Wang, Y., Chen, J., Wei, G., He, H., Zhu, X., Xiao, T., Yuan, J., Dong, B., He, S., Skogerbø, G., & Chen, R. The Caenorhabditis elegans intermediate-size transcriptome shows high degree of stage-specific expression. *Nucleic Acids Res* 39, 5203-14, (2011)
85. Chen, R. A.-J., Down, T. A., Stempor, P., Chen, Q. B., Egelhofer, T. A., Hillier, L. W., Jeffers, T. E., & Ahringer, J. The landscape of RNA polymerase II transcription initiation in C. elegans reveals promoter and enhancer architectures. *Genome Res* 23, 1339-47, (2013)
86. Willingham, A. T., Dike, S., Cheng, J., Manak, J. R., Bell, I., Cheung, E., Drenkow, J., Dumais, E., Duttagupta, R., Ganesh, M., Ghosh, S., Helt, G., Nix, D., Piccolboni, A., Sementchenko, V., Tammana, H., Kapranov, P., ENCODE Genes And Transcripts Group, & Gingeras, T. R. Transcriptional landscape of the human and fly genomes: nonlinear and multifunctional modular model of transcriptomes. *Cold Spring Harb Symp Quant Biol* 71, 101-10, (2006)
87. Langmead, B., Trapnell, C., Pop, M., & Salzberg, S. L. Ultrafast and memory-efficient alignment of short DNA sequences to the human genome. *Genome Biol* 10, R25, (2009)
88. Dobin, A., Davis, C. A., Schlesinger, F., Drenkow, J., Zaleski, C., Jha, S., Batut, P., Chaisson, M., & Gingeras, T. R. STAR: ultrafast universal RNA-seq aligner. *Bioinformatics* 29, 15-21, (2013)
89. Manak, J. R., Dike, S., Sementchenko, V., Kapranov, P., Biemar, F., Long, J., Cheng, J., Bell, I., Ghosh, S., Piccolboni, A., & Gingeras, T. R. Biological function of unannotated transcription during the early development of Drosophila melanogaster. *Nat Genet* 38, 1151-8, (2006)
90. Kim, T.-K., Hemberg, M., Gray, J. M., Costa, A. M., Bear, D. M., Wu, J., Harmin, D. A., Laptewicz, M., Barbara-Haley, K., Kuersten, S., Markenscoff-Papadimitriou, E., Kuhl, D., Bito, H., Worley, P. F., Kreiman, G., & Greenberg, M. E. Widespread transcription at neuronal activity-regulated enhancers. *Nature* 465, 182-7, (2010)
91. Yip, K. Y., Cheng, C., Bhardwaj, N., Brown, J. B., Leng, J., Kundaje, A., Rozowsky, J., Birney, E., Bickel, P., Snyder, M., & Gerstein, M. Classification of human genomic regions based on experimentally determined binding sites of more than 100 transcription-related factors. *Genome Biol* 13, R48, (2012)
92. modENCODE Consortium, Roy, S., Ernst, J., Kharchenko, P. V., Kheradpour, P., Negre, N., Eaton, M. L., Landolin, J. M., Bristow, C. A., Ma, L., Lin, M. F., Washietl, S., Arshinoff, B. I., Ay, F., Meyer, P. E., Robine, N., Washington, N. L., Di Stefano, L., Berezikov, E., Brown, C. D., Candeias, R., Carlson, J. W., Carr, A., Jungreis, I., Marbach, D., Sealfon, R., Tolstorukov, M. Y., Will, S., Alekseyenko, A. A., Artieri, C., Booth, B. W., Brooks, A. N., Dai, Q., Davis, C. A., Duff, M. O., Feng, X., Gorchakov, A. A., Gu, T., Henikoff, J. G., Kapranov, P., Li, R., MacAlpine, H. K., Malone, J., Minoda, A., Nordman, J., Okamura, K., Perry, M., Powell, S. K., Riddle, N. C., Sakai, A., Samsonova, A., Sandler, J. E., Schwartz, Y. B., Sher, N., Spokony, R., Sturgill, D., van Baren, M., Wan, K. H., Yang, L., Yu, C., Feingold, E., Good, P., Guyer, M., Lowdon, R., Ahmad, K., Andrews, J., Berger, B., Brenner, S. E., Brent, M. R., Cherbas, L.,

Elgin, S. C. R., Gingeras, T. R., Grossman, R., Hoskins, R. A., Kaufman, T. C., Kent, W., Kuroda, M. I., Orr-Weaver, T., Perrimon, N., Pirrotta, V., Posakony, J. W., Ren, B., Russell, S., Cherbas, P., Graveley, B. R., Lewis, S., Micklem, G., Oliver, B., Park, P. J., Celniker, S. E., Henikoff, S., Karpen, G. H., Lai, E. C., MacAlpine, D. M., Stein, L. D., White, K. P., & Kellis, M. Identification of functional elements and regulatory circuits by Drosophila modENCODE. *Science* 330, 1787-97, (2010)
93. Ren, B. Transcription: Enhancers make non-coding RNA. *Nature* 465, 173-4, (2010)
94. Boyle, A. P., Araya, C. L., Brdlik, C., Cayting, P., Cheng, C., Cheng, Y., Gardner, K., Hillier, L., Janette, J., Jiang, L., Kasper, D., Kawli, T., Kheradpour, P., Kundaje, A., Li, J. J., Ma, L., Niu, W., Rehm, E. J., Rozowsky, J., Slattery, M., Spokony, R., Terrell, R., Vafeados, D., Wang, D., Weisdepp, P., Wu, Y.-C., Xie, D., Yan, K.-K., Feingold, E. A., Good, P. J., Pazin, M. J., Huang, H., Bickel, P. J., Brenner, S. E., Reinke, V., Waterston, R. H., Gerstein, M., White, K. P., Kellis, M., Snyder, M., the modENCODE, & ENCODE Consortia Comparative analysis of regulatory information and circuits across distant species. *Nature* , , (2013)
95. Eisen, M. B., Spellman, P. T., Brown, P. O., & Botstein, D. Cluster analysis and display of genome-wide expression patterns. *Proc Natl Acad Sci U S A* 95, 14863-8, (1998)
96. Agrawal, H. & Domany, E. Potts ferromagnets on coexpressed gene networks: identifying maximally stable partitions. *Phys Rev Lett* 90, 158102, (2003)
97. Kluger, Y., Basri, R., Chang, J. T., & Gerstein, M. Spectral biclustering of microarray data: coclustering genes and conditions. *Genome Res* 13, 703-16, (2003)
98. Langfelder, P. & Horvath, S. WGCNA: an R package for weighted correlation network analysis. *BMC Bioinformatics* 9, 559, (2008)
99. Tamayo, P., Slonim, D., Mesirov, J., Zhu, Q., Kitareewan, S., Dmitrovsky, E., Lander, E. S., & Golub, T. R. Interpreting patterns of gene expression with self-organizing maps: methods and application to hematopoietic differentiation. *Proc Natl Acad Sci U S A* 96, 2907-12, (1999)
100. D'haeseleer, P. How does gene expression clustering work?. *Nat Biotechnol* 23, 1499-501, (2005)
101. Stuart, J. M., Segal, E., Koller, D., & Kim, S. K. A gene-coexpression network for global discovery of conserved genetic modules. *Science* 302, 249-55, (2003)
102. Hill, A. A., Hunter, C. P., Tsung, B. T., Tucker-Kellogg, G., & Brown, E. L. Genomic analysis of gene expression in C. elegans. *Science* 290, 809-12, (2000)
103. Murphy, C. T., McCarroll, S. A., Bargmann, C. I., Fraser, A., Kamath, R. S., Ahringer, J., Li, H., & Kenyon, C. Genes that act downstream of DAF-16 to influence the lifespan of Caenorhabditis elegans. *Nature* 424, 277-83, (2003)
104. Tomancak, P., Beaton, A., Weiszmann, R., Kwan, E., Shu, S., Lewis, S. E., Richards, S., Ashburner, M., Hartenstein, V., Celniker, S. E., & Rubin, G. M. Systematic determination of patterns of gene expression during Drosophila embryogenesis. *Genome Biol* 3, RESEARCH0088, (2002)
105. Tomancak, P., Berman, B. P., Beaton, A., Weiszmann, R., Kwan, E., Hartenstein, V., Celniker, S. E., & Rubin, G. M. Global analysis of patterns of gene expression during

Drosophila embryogenesis. *Genome Biol* 8, R145, (2007)
106. Jiang, M., Ryu, J., Kiraly, M., Duke, K., Reinke, V., & Kim, S. K. Genome-wide analysis of developmental and sex-regulated gene expression profiles in Caenorhabditis elegans. *Proc Natl Acad Sci U S A* 98, 218-23, (2001)
107. Kim, S. K., Lund, J., Kiraly, M., Duke, K., Jiang, M., Stuart, J. M., Eizinger, A., Wylie, B. N., & Davidson, G. S. A gene expression map for Caenorhabditis elegans. *Science* 293, 2087-92, (2001)
108. Arbeitman, M. N., Furlong, E. E. M., Imam, F., Johnson, E., Null, B. H., Baker, B. S., Krasnow, M. A., Scott, M. P., Davis, R. W., & White, K. P. Gene expression during the life cycle of Drosophila melanogaster. *Science* 297, 2270-5, (2002)
109. Stolc, V., Gauhar, Z., Mason, C., Halasz, G., van Batenburg, M. F., Rifkin, S. A., Hua, S., Herreman, T., Tongprasit, W., Barbano, P. E., Bussemaker, H. J., & White, K. P. A gene expression map for the euchromatic genome of Drosophila melanogaster. *Science* 306, 655-60, (2004)
110. Spencer, W. C., Zeller, G., Watson, J. D., Henz, S. R., Watkins, K. L., McWhirter, R. D., Petersen, S., Sreedharan, V. T., Widmer, C., Jo, J., Reinke, V., Petrella, L., Strome, S., Von Stetina, S. E., Katz, M., Shaham, S., Rätsch, G., & Miller, 3rd, D. M. A spatial and temporal map of C. elegans gene expression. *Genome Res* 21, 325-41, (2011)
111. Domazet-Lošo, T. & Tautz, D. A phylogenetically based transcriptome age index mirrors ontogenetic divergence patterns. *Nature* 468, 815-8, (2010)
112. Ruan, J., Dean, A. K., & Zhang, W. A general co-expression network-based approach to gene expression analysis: comparison and applications. *BMC Syst Biol* 4, 8, (2010)
113. Newman, M. E. J. Modularity and community structure in networks. *Proc Natl Acad Sci U S A* 103, 8577-82, (2006)
114. Traag, V. A. & Bruggeman, J. Community detection in networks with positive and negative links. *Phys Rev E Stat Nonlin Soft Matter Phys* 80, 036115, (2009)
115. Reichardt, J. & Bornholdt, S. Detecting fuzzy community structures in complex networks with a Potts model. *Phys Rev Lett* 93, 218701, (2004)
116. Davidson, E. H. & Erwin, D. H. Gene regulatory networks and the evolution of animal body plans. *Science* 311, 796-800, (2006)
117. Trapnell, C., Williams, B. A., Pertea, G., Mortazavi, A., Kwan, G., van Baren, M. J., Salzberg, S. L., Wold, B. J., & Pachter, L. Transcript assembly and quantification by RNA-Seq reveals unannotated transcripts and isoform switching during cell differentiation. *Nat Biotechnol* 28, 511-5, (2010)
118. Karlić, R., Chung, H.-R., Lasserre, J., Vlahovicek, K., & Vingron, M. Histone modification levels are predictive for gene expression. *Proc Natl Acad Sci U S A* 107, 2926-31, (2010)
119. Cheng, C., Yan, K.-K., Yip, K. Y., Rozowsky, J., Alexander, R., Shou, C., & Gerstein, M. A statistical framework for modeling gene expression using chromatin features and application to modENCODE datasets. *Genome Biol* 12, R15, (2011)

120. Dong, X., Greven, M. C., Kundaje, A., Djebali, S., Brown, J. B., Cheng, C., Gingeras, T. R., Gerstein, M., Guigó, R., Birney, E., & Weng, Z. Modeling gene expression using chromatin features in various cellular contexts. *Genome Biol* 13, R53, (2012)
121. Ouyang, Z., Zhou, Q., & Wong, W. H. ChIP-Seq of transcription factors predicts absolute and differential gene expression in embryonic stem cells. *Proc Natl Acad Sci U S A* 106, 21521-6, (2009)
122. Cheng, C. & Gerstein, M. Modeling the relative relationship of transcription factor binding and histone modifications to gene expression levels in mouse embryonic stem cells.. *Nucleic Acids Res* 40, 553--568, (2012)
123. Cheng, C., Shou, C., Yip, K. Y., & Gerstein, M. B. Genome-wide analysis of chromatin features identifies histone modification sensitive and insensitive yeast transcription factors.. *Genome Biol* 12, R111, (2011)
124. Costa, I. G., Roider, H. G., do Rego, T. G., & de Carvalho, F. d. A. T. Predicting gene expression in T cell differentiation from histone modifications and transcription factor binding affinities by linear mixture models. *BMC Bioinformatics* 12 Suppl 1, S29, (2011)
125. Giannopoulou, E. G. & Elemento, O. Inferring chromatin-bound protein complexes from genome-wide binding assays. *Genome Res* 23, 1295-306, (2013)
126. Kurdistani, S. K., Tavazoie, S., & Grunstein, M. Mapping global histone acetylation patterns to gene expression. *Cell* 117, 721-33, (2004)
127. Breiman, L. Radom Forests. *Machine Learning* 45, 5-32, (2001)
128. Vaquerizas, J. M., Kummerfeld, S. K., Teichmann, S. A., & Luscombe, N. M. A census of human transcription factors: function, expression and evolution. *Nat Rev Genet* 10, 252-63, (2009)
129. Reece-Hoyes, J. S., Deplancke, B., Shingles, J., Grove, C. A., Hope, I. A., & Walhout, A. J. M. A compendium of Caenorhabditis elegans regulatory transcription factors: a resource for mapping transcription regulatory networks. *Genome Biol* 6, R110, (2005)
130. Adryan, B. & Teichmann, S. A. FlyTF: a systematic review of site-specific transcription factors in the fruit fly Drosophila melanogaster. *Bioinformatics* 22, 1532-3, (2006)
131. Cheng, C., Alexander, R., Min, R., Leng, J., Yip, K. Y., Rozowsky, J., Yan, K.-K., Dong, X., Djebali, S., Ruan, Y., Davis, C. A., Carninci, P., Lassman, T., Gingeras, T. R., Guigó, R., Birney, E., Weng, Z., Snyder, M., & Gerstein, M. Understanding transcriptional regulation by integrative analysis of transcription factor binding data. *Genome Res* 22, 1658-67, (2012)

Supplementary Tables

Table S1 - Tables Giving More Detail on “Comparative ENCODE RNA Resource”.

Table S1a - Tables of human RNA-Seq data.

Table S1a1 - Human RNA-Seq data: RNA types and compartment comparison.

	In Djebali *et al.* (2012)		
	**No, it's new**	**Yes**	**Total**
**Poly-A(-)**			
Cell	5	29	34
Cytoplasm	0	11	11
Nucleus	0	13	13
Subtotal	5	53	58
**Poly-A(+)**			
Cell	9	56	65
Cytoplasm	4	16	20
Nucleus	4	19	23
Subtotal	17	91	108
**Total RNA**			
Cell	38	1	39
Cytoplasm	0	0	0
Nucleus	0	0	0
Subtotal	38	1	39
Total	***60***	***145***	***205***

Table S1a2 - Human RNA-Seq data: immortalized cell type comparison.

**Immortalized**	**In Djebali *et al.* (2012)**		
	**No, it's new**	**Yes**	**Total**
**Adenocarcinoma**			
MCF-7	0	10	10
**Carcinoma**			
A549	0	8	8
Hela-S3	0	13	13
HepG2	0	14	14
**Erythroleukemia**			
K562	0	14	14
**Neuroblastoma**			
SK-N-SH	6	0	6
SK-N-SH_RA	0	4	4
**Lymphoblastoid + EBV**			
GM12878	0	15	15
GM12891	0	2	2
GM12892	0	3	3
# Libraries	***6***	***83***	***89***
# Cell Line	***1***	***9***	***10***

Table S1a3 - Human RNA-Seq data: primary cell type comparison.

Primary	In Djebali *et al.* (2012)		
	No, it's new	Yes	Total
**B Cells**			
CD20+	4	0	4
**Chondrocyte**			
HCH	2	0	2
**Endothelial**			
HAoEC	2	0	2
HSaVEC	2	0	2
HUVEC	0	13	13
**Epithelial**			
HMEC	2	0	2
HMEpC	0	1	1
HPIEpC	2	0	2
**Fibroblast**			
AG04450	0	4	4
BJ	0	4	4
HAoAF	2	0	2
HVMF	2	0	2
IMR90	8	0	8
NHDF	2	0	2
NHLF	0	6	6
**Follicle Dermal Papilla**			
HFDPC	2	0	2
**Keratinocyte**			
NHEK	0	15	15

**Melanocyte**			
NHEM_M2	2	0	2
NHEM.f_M2	2	0	2
**Mesenchylam Stem**			
hMSC-AT	2	0	2
hMSC-BM	2	0	2
hMSC-UC	2	0	2
**Monocyte**			
CD14+	4	0	4
**Myoblast**			
HSMM	0	6	6
**Mononuclear Cells**			
hMNC-PB	1	0	1
**Osteoblast**			
HOB	2	0	2
**Pericytes**			
HPC-PL	2	0	2
**Preadipocyte**			
HWP	2	0	2
**Progenitor**			
CD34+	1	0	1
**Skeletal Muscle**			
SkMC	2	0	2
**Embryonic Stem Cell**			
H1-hESC	0	13	13
# Libraries	***54***	***62***	***116***
# Cell Lines	***23***	***8***	***31***

Table S1a4 - Human RNA-Seq data: tissue type comparison.

**Tissue**	**In Djebali *et al.* (2012)**		
	**No, it's new**	**Yes**	**Total**
Adipose	4	0	4
Blood	10	34	44
Bone	2	0	2
Bone Marrow	2	0	2
Brain	6	4	10
Breast	2	11	13
Cartilage	2	0	2
Cervix	0	13	13
Heart	4	0	4
Liver	0	14	14
Lung	8	18	26
Placenta	4	0	4
Saphenous Vein	2	0	2
Skeletal Muscle	2	6	8
Skin	8	19	27
Umbilical Cord	2	0	2
Umbilical Vein	0	13	13
Villious Placenta	2	0	2
Stem Cell	0	13	13
New Tissues	***15***	***4***	***19***
Total	***60***	***145***	***205***

Table S1b - Tables of broadly expressed protein-coding genes.

Table S1b1 - Most enriched common GO terms in broadly expressed coding genes for human, worm and fly.

		Human			Fly			Worm		
GO id	GO term	Count in data set	Count in genome	Adjusted p-value	Count in data set	Count in genome	Adjusted p-value	Count in data set	Count in genome	Adjusted p-value
GO:0006396	RNA processing	477	578	7.10E-117	295	391	6.37E-27	119	142	7.04E-14
GO:0030529	ribonucleoprotein complex	430	504	1.28E-88	370	462	3.99E-33	167	186	1.99E-59
GO:0044428	nuclear part	1,259	1,938	1.57E-91	523	754	6.66E-20	147	198	8.95E-33
GO:0044446	intracellular organelle part	2,676	5,019	3.42E-59	1,245	2,035	3.62E-15	451	709	1.36E-65
GO:0070013	intracellular organelle lumen	1,233	1,845	2.29E-101	367	523	1.65E-15	92	121	6.19E-22
GO:0031974	membrane-enclosed lumen	1,257	1,917	4.04E-95	376	535	5.95E-16	98	128	1.01E-23
GO:0043233	organelle lumen	1,234	1,881	9.64E-94	367	523	1.65E-15	93	123	6.97E-22
GO:0044422	organelle part	2,692	5,089	7.73E-56	1,251	2,049	6.98E-15	460	749	3.17E-60
GO:0032991	macromolecular complex	1,724	3,237	8.68E-38	1,219	2,016	8.02E-13	508	780	5.16E-79
GO:0005840	ribosome	178	198	8.08E-44	152	177	2.87E-19	126	142	1.38E-43
GO:0031981	nuclear lumen	1,005	1,518	1.07E-79	245	362	3.72E-08	80	107	2.23E-18
GO:0003735	structural constituent of ribosome	144	158	8.67E-44	149	172	3.27E-34	125	141	4.61E-34

Table S1b2 - Number and percentages of broadly expressed genes in different classes.

	Coding	Non-coding	Pseudogene	TAR
**Human**	6,912 (35%)	1,448 (4.4%)	569 (5.10%)	2,419 (0.34%)
**Worm**	5,180 (25%)	1,513 (3.6%)	6 (0.69%)	527 (0.22%)
**Fly**	5,288 (38%)	170 (7.8%)	5 (4.60%)	990 (1.30%)

Table S1b3 - Spearman correlation of different expression scores.

This table shows the correlation between developmental stages and other modENCODE RNA-Seq samples. The most stable score when using different sample sets is identified to be coefficient of variation of log2(nRPKM + 1).

	**Worm**	**Fly**
Min(RPKM)	0.648	0.845
CV(RPKM)	0.763	0.815
Entropy(RPKM)	0.653	0.594
CV(log_2(RPKM+1))	0.907	0.907
Min(nRPKM)	0.654	0.850
CV(nRPKM)	0.755	0.835
Entropy(nRPKM)	0.669	0.646
CV(log_2(nRPKM+1))	0.922	0.911
Min(TMM.RPKM)	0.659	0.853
CV(TMM.RPKM)	0.739	0.787
Entropy(TMM.RPKM)	0.628	0.459
CV(log_2(TMM.RPKM+1))	0.915	0.904

Table S1c - Tables of splicing annotation.

Table S1c1 - Counts of splicing events for human, worm, and fly.

Event Type	Organism		
	Human	Worm	Fly
Mutually Exclusive (MXE)	16	4	59
Coordinate Skipped Exons (CSE)	92	74	12
Skipped Exons (SE)	30,464	4,136	4,231
Retained Introns (RI)	6,215	7,412	6,271
Alternative 3' Splice Sites (A3SS)	7,804	4,404	3,248
Alternative 5' Splice Sites (A5SS)	4,703	3,348	3,929
Alternative First Exons (AFE)	11,404	1,793	2,077
Alternative Last Exons (ALE)	5,547	586	381
Tandem UTRs	22,247	8,868	5,548
Total	***88,492***	***30,625***	***25,756***

Table S1c2 - Number of genes with all splice sites conserved, for pairwise comparisons in orthologs.

1803 1-1-1 orthologous genes were analyzed considering only the protein-coding regions. There are no genes with all splice sites conserved across all 3 species.

#Genes w/ all splices conserved	Organisms		
	Human	Worm	Fly
**Human**	1,803	3	14
**Worm**		1,803	1
**Fly**			1,803

Table S1c3 - Number of genes with at least one conserved splice sites, for pairwise comparisons in orthologs.

1803 1-1-1 orthologous genes were analyzed considering only the protein-coding regions. We found 418 genes with at least one conserved splice sites across all 3 species.

#Genes w/ 1+ splices conserved	Organisms		
	Human	Worm	Fly
**Human**	1,803	990	1,053
**Worm**		1,803	504
**Fly**			1,803

Table S1c4 - Number of conserved splice sites, for pairwise comparisons in orthologs.

1803 1-1-1 orthologous genes were analyzed considering only the protein-coding regions. There are 573 conserved splice sites across all 3 species.

#Conserved splices	Organisms		
	**Human**	**Worm**	**Fly**
**Human**	23,270	2,310	2,169
**Worm**		12,975	699
**Fly**			5,877

Table S2 - Tables Giving More Details on “ncRNAs & Non-Canonical Transcription”.

Table S2a (Shadow for ED Fig. 3) - Table of annotated ncRNAs in human, worm, and fly.

We present estimates for the amount of different categories of ncRNAs in each of the three organisms. This transcription is also subdivided into the fraction of RNA-Seq reads that occurs within introns of protein-coding genes as well as the fraction that overlaps transposable elements.

			Human					Worm				Fly			
			Elements	Genome Coverage		RNA Read Coverage (%)		Elements	Genome Coverage		RNA Read Coverage	Elements	Genome Coverage		RNA Read Coverage
				Kb	%	Poly(A)	Total		Kb	%	Poly(A) [%]		Kb	%	Poly(A) [%]
Annotated ncRNAs	Comparable ncRNAs	pri-miRNA	58	1,158	0.04	0.036	0.025	44	16	0.02	0.00066	43	300	0.23	0.017
		pre-miRNAs	1,756	162	0.006	0.27	4.35	221	20	0.02	0.021	236	22	0.02	0.0071
		tRNAs	624	47	0.002	0.031	0.38	609	45	0.04	0.0012	314	22	0.02	0.00013
		snoRNAs	1,521	168	0.006	0.033	0.10	141	16	0.02	0.029	287	34	0.03	0.029
		snRNAs	1,944	210	0.007	0.0046	0.018	114	14	0.01	0.0049	47	7	0.006	0.0085
		lncRNAs	10,840	10,581	0.37	3.17	1.75	233	184	0.18	0.072	852	868	0.68	1.22
	Other ncRNAs		5,411	3,268	0.11	0.97	34.32	40,104	2,329	2.3	10.51	376	2,103	1.6	2.48
		piRNA loci	88	1,272	0.04	0.032	0.0073	35,329	449	0.45	0.67	27	1,473	1.1	0.16
Total ncRNAs			22,154	17,770	0.62	4.45	40.52	41,466	2,611	2.6	10.61	2,155	3,279	2.6	3.74
Regions Excluding mRNAs, Pseudogenes or Annotated ncRNAs			283,816	2,731,811	95.5	20.29	41.62	143,372	63,520	63.3	4.14	60,108	89,445	69.6	5.52
	Transcription Detected (TARs)		708,253	916,401	32.0	19.96	41.34	232,150	37,029	36.9	4.12	83,618	44,256	34.5	5.50
		Supervised Predictions	104,016	13,835	0.48	0.78	6.37	2,525	392	0.39	0.015	599	164	0.13	0.0016
		Overlapping TEs	1,616,444	451,416	15.8	7.48	29.79	64,694	5,510	5.5	0.22	36,839	6,935	5.4	2.89
		In Introns	515,803	667,440	23.3	14.91	19.52	153,517	21,585	21.5	2.38	61,601	30,389	23.7	3.24

Table S2b - TAR classifications.

Table S2b1 - Classification of TARs with respect to genomic location.

We classified TARs with respect to genomic location. The TAR regions in each species (rows) are divided into 7 different classes. In each cell, number of elements are reported above and coverage of the elements are reported (in kb) in parenthesis. We also included the TARs that are clustered into one of the expression modules. For all three organisms the intronic TARs cover the largest fraction. In worm and fly, promoter associated TARs has a larger fraction than intergenic TARs. In human, intergenic TARs take larger fraction of all TARs than promoter associated TARs. Also in worm and fly, enhancer associated TARs cover a larger fraction than intergenic TARs, whereas in human there is significantly less enhancer associated TARs. Enhancer associated TARs in each organism can be used as the eRNA annotations in each organism.

	HOT	Intronic	Intergenic	incRNA	Promoter	Enhancer	Clustered
Human	7991<br>(2,300)	444,507<br>(678,905)	226,853<br>(139,924)	73,726<br>(35,537)	56,092<br>(121,204)	26,627<br>(15,682)	8189<br>(20,825)
Worm	1770<br>(671)	152,880<br>(21,770)	13,871<br>(1,783)	2234<br>(1,891)	192,859<br>(31,009)	34,245<br>(12,116)	10912<br>(6,893)
Fly	187<br>(313)	61,278<br>(30,522)	7802<br>(5,297)	402<br>(1,133)	43,956<br>(18,204)	12,066<br>(16,370)	1496<br>(1,801)

Table S2b2 - Classification of clustered TARs with respect to genomic location.

We classified all the TARs that are clustered into one of the expression clusters into one of the incRNA, Enhancer or HOT regions. The enhancer associated TARs can be assigned a putative function in relation to the genes in the cluster.

	Intronic Clustered TARs				Intergenic Clustered TARs				
	Total	incRNA	Enhancer	HOT	Total	incRNA	Promoter	Enhancer	HOT
Human	6906 (17148)	1352 (8550)	393 (3663)	184 (658)	1283 (3677)	239 (1716)	263 (695)	67 (545)	58 (229)
Worm	7692 (4594)	246 (360)	2011 (2216)	60 (86)	3220 (2299)	166 (375)	2707 (2017)	678 (1135)	132 (134)
Fly	1142 (1459)	53 (185)	338 (838)	11 (35)	354 (342)	1 (7)	219 (212)	75 (147)	2 (14)

Table S2c - Gold standard ncRNA numbers for supervised ncRNA predictions.

		Fly			Worm			Human		
**ncRNA types**	**ncRNA subtype**	**Raw annotation**	**Bins**	**Nucleotides covered**	**Raw annotation**	**Bins**	**Nucleotides covered**	**Raw annotation**	**Bins**	**Nucleotides covered**
**ncRNA1**	RNA	96	262	12,958	22	228	11,414	531	1,202	60,053
	RNA	292	456	22,169	609	909	45,577	625	938	46,643
	nRNA	47	135	7,230	114	286	14,311	1,944	4,209	210,373
	noRNA	286	691	33,994	139	309	15,306	1,521	3,325	168,125
	niRNA	237	422	22,291	222	406	19,701	1,756	2,668	129,201
	nlRNA	-	-	-	4	7	348	-	-	-
	cRNA	-	-	-	1	3	105	-	-	-
	SK_RNA	-	-	-	-	-	-	298	1,767	88,751
	_RNA	-	-	-	-	-	-	809	1,675	83,999
**RNA2**	sense[a]	94	1,989	129,950	48	737	49,679	72	695	83,845
	ronic[b]	17	166	12,981	4	53	3,663	-	-	-
	genic[c]	147	2,256	199,547	136	2,379	176,002	460	3,935	565,365
	verlapped[d]	33	492	42,091	-	-	-	7,343	121,010	133,333,108
	iguous[e]	26	1,400	37,794	23	474	37,106	30	577	47,243

a, More than 50% of ncRNA fragments overlapped with known coding transcripts on the opposite strand.
b, ncRNA fragments were fully embeded in coding gene's intron on the same strand.
c, ncRNA fragments were fully embeded in intergenic region.
d, More than one nucleotide ncRNA fragments overlapped with transposable elements.
e, ncRNAs located in none of the above region.

Table S2d - Numbers of predicted ncRNA candidates in three organisms, sensitivity cut off 0.95.

Species	Data type	ncRNA type[a]	Number of bins (After filter)[b]	Number of merged fragments (length >=200nt)[c]	Number of nucleotides[d]
**Fly**	All	ncRNA1	271	96(29)	19,200
		ncRNA2	5,466	1,517(695)	374,300
	Embryo	ncRNA1	137	42(11)	9,950
		ncRNA2	2,491	436(299)	164,700
**Worm**	All	ncRNA1	4,367	3,250(262)	313,700
		ncRNA2	8,747	2,554(1,182)	446,300
	Embryo	ncRNA1	3,907	2,929(250)	285,650
		ncRNA2	8,567	2,432(1,170)	437,100
**Human**	All	ncRNA1	463,746	273,209(12,103)	36,585,750
		ncRNA2	36,770	12,107(4,311)	2,071,050
	H1_ESC	ncRNA1	306,877	173,736(8,762)	24,208,200
		ncRNA2	11,476	2,915(1,134)	736,100

a, Two types of ncRNA predicted from canonical ncRNA learning model and lncRNA learning model separately.
b, Predicted ncRNA bins overlapped with UTR and CDS on the same strand were filtered out, bins overlapped with known ncRNAs on both strand were filtered out.
c, Merge adjacent ncRNA bins allowing 50 nt gaps, longer than 200nt as long ncRNA fragment.
d, ncRNA fragments covered nucleotide number.
Note these numbers were not including ambiguous ncRNAs (defined above).

Table S2e - ncRNA fragment genomic location.

Species	Data type	ncRNA type	Antisense[a]	Intronic[b]	Intergenic[c]	Overlap with TE[d]	Overlap with pseudogene[e]	Ambiguous[f]
**Fly**	All	ncRNA1	36	31	24	62	5	0
		ncRNA2	746	515	256	20	0	27(13)
	Embryo	ncRNA1	11	23	7	2	1	-
		ncRNA2	209	187	40	6	-	10(3)
**Worm**	All	ncRNA1	735(162)[g]	522(64)	1,930	55	63(12)	1,352(671)
		ncRNA2	623(3)	387(9)	1,525	16	19	136(10)
	Embryo	ncRNA1	683(148)	482(60)	1,717	44	47(8)	1,254(623)
		ncRNA2	608(3)	376(9)	1,429	16	19	135(10)
**Human**	All	ncRNA1	82,555	60,914	122,048	720,181	7,692	3,419
		ncRNA2	1,372(2)	943	8,118	48,551(72)	1,674	619(24)
	H1_ESC	ncRNA1	47,344	52,264	69,140	364,098	4,988	2,130
		ncRNA2	328(2)	325	1,958	26,527(59)	304	270(13)

a, More than 50% of ncRNA fragments overlapped with known coding transcripts on the opposite strand.
b, ncRNA fragments were fully embeded in coding gene's intron on the same strand.
c, ncRNA fragments were fully embeded in intergenic region.
d, More than one nucleotide ncRNA fragments overlapped with transposable elements.
e, More than one nucleotide ncRNA fragments overlapped with pseudogenes.
f, None of the above.
g. Numbers in the parentheses are those predicted ncRNA fragments already annotated in the database (i.e. wormbase, flybase and gencode) but not confirmed or validated. They were not included in the gold-standard training set.

Table S2f - Supervised ncRNA predictions training on whole genome compared with previous predictions training on conserved regions of C.elegans.

		Overlap with 95% sensitivity predictions			Overlap with 99% sensitivity predictions			Overlap with max ncRNA score bins(no cutoff)			Pearson Correlation coefficient	
	Previous Predictions	In ncRNA1	In ncRNA2	In ncRNA1 or ncRNA2	In ncRNA1	In ncRNA2	In ncRNA1 or ncRNA2	In ncRNA1	In ncRNA2	In ncRNA1 or ncRNA2	ncRNA1 _score	ncRNA2 _score
**All high**	7,237	22%	1%	23%	43%	10%	49%	80%	78%	94%	0.21	-0.06
**confident**	704	51%	0%	51%	65%	7%	68%	86%	74%	95%	0.32	-0.10
**All medium confident**	6,533	19%	1%	20%	40%	11%	47%	79%	78%	94%	0.31	-0.05
**Intergenic**	2,262	22%	1%	23%	46%	12%	54%	85%	82%	97%	0.07	-0.07
**Intergenic high confident**	143	23%	1%	24%	49%	12%	56%	82%	80%	97%	0.02	-0.04
**Intergenic medium confident**	2,119	21%	1%	23%	46%	12%	53%	85%	82%	97%	0.09	-0.08

Table S2g - Annotation source.

	Human	Fly	Worm
Gold-standard (Coding sequence, UTR, etc)	Gencode V10 (level 1 and 2)	FlyBase R5.45 (confirmed)	WormBase WS220 (confirmed)
Gold-standard (ncRNA)	Gencode V10 (level 1 and 2)	FlyBase R5.45 (with support of EST, cDNA expression, etc)	WormBase WS220 (lncRNAs from [25])
Extended annotation	Gencode V10 (all levels)	modENCODE freeze	modENCODE freeze

Table S2h - Intersection of enhancers and distal HOT regions with TARs and incRNAs.

Intersection	Human			Worm			Fly		
	Overlap[a]	Ratio[b]	Z-score[c]	Overlap	Ratio	Z-score	Overlap	Ratio	Z-score
Enhancer vs. TARs	128,400	1.7	250	15,863	1.0	11	10,380	1.4	46
Enhancer vs. incRNA	14,357	2.8	125	590	1.7	13	229	3.5	20
Distal HOT vs. TARs	23,073	1.7	113	520	1.1	4	435	1.4	11
Distal HOT vs. incRNA	4,604	4.6	116	49	4.1	11	7	2.6	3

a. The observed number of overlap between enhancers or distal HOT regions and TARs or incRNAs.
b. The ratio of observed overlap to the overlap from randomization.
c. The z-scores of the observed overlaps.

Table S3 - Tables Giving More Details on "Expression Clustering & Stage Alignment".

Table S3a - Statistics related to each of the conserved modules.

Module	Protein-coding genes			Rank (# of genes)	1-1-1 orthologs	ncRNAs (cor+anti-cor)[1]			TARs (cor+anti-cor)[2]		
	Human	Worm	Fly			Human	Worm	Fly	Human	Worm	Fly
1	709	336	346	1	37	401+85	27+24	455+203	329+457	1726+188	253+300
2*	197	158	159	4	58	804+148	5+14	237+189	954+216	43+969	131+992
3*	66	65	65	8	45	549+24	14+14	190+173	217+325	10+1578	36+965
4*	176	133	132	5	54	474+261	12+26	197+238	429+430	53+743	75+780
5*	378	262	222	2	29	675+39	30+18	260+64	281+95	1333+216	652+72
6*	65	55	52	10	25	634+46	14+22	109+139	634+216	30+4422	64+1184
7*	268	269	253	3	135	1002+66	16+28	228+243	2229+582	137+4724	178+1940
8	30	35	37	16	17	86+28	18+13	40+43	55+112	3+3707	3+166
9*	100	94	96	6	51	657+16	16+1	131+64	123+184	3+229	1+55
10*	34	39	33	15	25	471+21	12+14	170+154	235+682	33+2116	30+1000
11*	39	37	41	13	29	302+17	11+10	154+201	143+201	1+480	26+1251
12*	48	42	38	12	19	185+5	7+20	79+185	522+25	23+3450	67+1513
13*	73	75	80	7	17	607+15	13+17	160+203	422+362	2+1086	2+571
14	72	63	47	9	10	138+6	21+15	150+52	23+392	1147+65	4+249
15	32	41	38	14	18	159+6	7+21	104+115	189+53	34+2987	56+898
16*	53	56	58	11	42	702+12	7+6	167+182	219+74	8+447	40+1027

1. Numbers of co-expressed ncRNAs with one of 1-1-1 orthologous genes in each module with correlation > 0.65 or anti-correlation < -0.65
2. Numbers of co-expressed TARs with one of 1-1-1 orthologous genes in each module with correlation > 0.9 or anti-correlation < -0.80 (worm, fly) or < -0.90 (human)
* Hour-glass modules

Table S3b - Early embryo specific worm genes that are aligned to both fly genes in specific in embryos (18-20hr) and pupae.

The worm genes are shown in the first column, and the corresponding GO terms in second. Their corresponding fly orthologs enriched in fly embryo18_20h are shown in the 3rd columns (GO terns in 4th). Orthologs enriched in fly pupae are shown in 5rd columns (GO terns in 6th).

Worm gene common in 2 stages: ID	Worm gene common in 2 stages: GO terms	Fly gene Embroyo18-20hr ID	Fly gene Embroyo18-20hr GO terms	Fly gene Prepupae+2d ID	Fly gene Prepupae+2d GO terms
**WBGene00000042**	synaptic transmission, ion transport	FBgn0032151	ion transport, response to insecticide, muscle cell homeostasis	FBgn0086778	jump response, synaptic transmission, ion transport, regulation of excitatory postsynaptic membrane potential, visual behavior
**WBGene00000043**	ion transport	FBgn0032151	ion transport, response to insecticide, muscle cell homeostasis	FBgn0086778	jump response, synaptic transmission, ion transport, regulation of excitatory postsynaptic membrane potential,visual behavior
**WBGene00000047**	ion transport	FBgn0032151	ion transport, response to insecticide, muscle cell homeostasis	FBgn0086778	jump response, synaptic transmission, ion transport, regulation of excitatory postsynaptic membrane potential, visual behavior
**WBGene00000051**	ion transport	FBgn0032151	ion transport, response to insecticide, muscle cell homeostasis	FBgn0086778	jump response, synaptic transmission, ion transport, regulation of excitatory postsynaptic membrane potential,visual behavior
**WBGene00000279**		FBgn0029962		FBgn0029962	
**WBGene00000280**		FBgn0029962		FBgn0029962	

**WBGene00000488**	lipid storage	FBgn0085387	jump response, response to light stimulus, gap junction assembly, phototransduction, intercellular transport, cell communication by electrical coupling, regulation of membrane depolarization	FBgn0085387	jump response, response to light stimulus, gap junction assembly, phototransduction, intercellular transport, cell communication by electrical coupling, regulation of membrane depolarization
**WBGene00001136**	growth	FBgn0085387	jump response, response to light stimulus, gap junction assembly, phototransduction, intercellular transport, cell communication by electrical coupling, regulation of membrane depolarization	FBgn0085387	jump response, response to light stimulus, gap junction assembly, phototransduction, intercellular transport, cell communication by electrical coupling, regulation of membrane depolarization
**WBGene00001202**	potassium ion transport, protein homooligomerization, ion transport, transmembrane transport, protein catabolic process, ion transmembrane transport	FBgn0003386	potassium ion transport, transmembrane transport, protein homooligomerization, sleep	FBgn0085395	protein homooligomerization, transmembrane transport, potassium ion transport
**WBGene00001629**	protein O-linked glycosylation via threonine	FBgn0031530	oligosaccharide biosynthetic process	FBgn0031530	oligosaccharide biosynthetic process
**WBGene00001630**	protein O-linked glycosylation via threonine	FBgn0050463	protein O-linked glycosylation, multicellular organism reproduction	FBgn0050463	protein O-linked glycosylation, multicellular organism reproduction

**WBGene00002123**		FBgn0085387	jump response, response to light stimulus, gap junction assembly, phototransduction, intercellular transport, cell communication by electrical coupling, regulation of membrane depolarization	FBgn0085387	jump response, response to light stimulus, gap junction assembly, phototransduction, intercellular transport, cell communication by electrical coupling, regulation of membrane depolarization
**WBGene00002128**		FBgn0085387	jump response, response to light stimulus, gap junction assembly, phototransduction, intercellular transport, cell communication by electrical coupling, regulation of membrane depolarization	FBgn0085387	jump response, response to light stimulus, gap junction assembly, phototransduction, intercellular transport, cell communication by electrical coupling, regulation of membrane depolarization
**WBGene00002132**		FBgn0085387	jump response, response to light stimulus, gap junction assembly, phototransduction, intercellular transport, cell communication by electrical coupling, regulation of membrane depolarization	FBgn0085387	jump response, response to light stimulus, gap junction assembly, phototransduction, intercellular transport, cell communication by electrical coupling, regulation of membrane depolarization
**WBGene00002140**	embryo development ending in birth or egg hatching, embryo development	FBgn0085387	jump response, response to light stimulus, gap junction assembly, phototransduction, intercellular transport, cell communication by electrical coupling, regulation of membrane depolarization	FBgn0085387	jump response, response to light stimulus, gap junction assembly, phototransduction, intercellular transport, cell communication by electrical coupling, regulation of membrane depolarization

**WBGene00002141**	positive regulation of multicellular organism growth, determination of left/right asymmetry in nervous system, intercellular transport, negative regulation of protein kinase activity, inactivation of MAPKKK activity, inactivation of MAPKKK activity	FBgn0085387	jump response, response to light stimulus, gap junction assembly, phototransduction, intercellular transport, cell communication by electrical coupling, regulation of membrane depolarization	FBgn0085387	jump response, response to light stimulus, gap junction assembly, phototransduction, intercellular transport, cell communication by electrical coupling, regulation of membrane depolarization
**WBGene00002974**	ion transport, transport, regulation of locomotion, ion transmembrane transport, regulation of oviposition	FBgn0032151	ion transport, response to insecticide, muscle cell homeostasis	FBgn0086778	jump response, synaptic transmission, ion transport, regulation of excitatory postsynaptic membrane potential, jump response, synaptic transmission, visual behavior
**WBGene00002983**	carbohydrate metabolic process	FBgn0261341	carbohydrate metabolic process, chitin metabolic process, open tracheal system development, regulation of tube length, open tracheal system, open tracheal system development, regulation of tube length, open tracheal system, dorsal trunk growth, open tracheal system, trachea morphogenesis, visual behavior	FBgn0032598	carbohydrate metabolic process, chitin metabolic process
**WBGene00003166**	mechanosensory behavior, response to mechanical stimulus	FBgn0030992	phagocytosis, engulfment	FBgn0260657	biological_process
**WBGene00004370**		FBgn0031627		FBgn0053543	cell adhesion

**WBGene00004793**	potassium ion transport, protein homooligomerization, ion transport, transmembrane transport, cellular potassium ion transport	FBgn0003386	transmembrane transport, protein homooligomerization, potassium ion transport, sleep	FBgn0085395	protein homooligomerization, transmembrane transport, potassium ion transport
**WBGene00006066**		FBgn0030992	phagocytosis, engulfment	FBgn0260657	biological_process
**WBGene00006068**		FBgn0030992	phagocytosis, engulfment	FBgn0260657	biological_process
**WBGene00006690**	potassium ion transmembrane transport	FBgn0033257	potassium ion transmembrane transport	FBgn0027589	potassium ion transmembrane transport
**WBGene00006747**	locomotion, protein catabolic process, response to chemical stimulus, oviposition, gap junction assembly, regulation of pharyngeal pumping, regulation of pharyngeal pumping, regulation of pharyngeal pumping, regulation of pharyngeal pumping, regulation of pharyngeal pumping, ion transmembrane transport	FBgn0085387	jump response, response to light stimulus, gap junction assembly, phototransduction, intercellular transport, cell communication by electrical coupling, regulation of membrane depolarization	FBgn0085387	jump response, response to light stimulus, gap junction assembly, phototransduction, intercellular transport, cell communication by electrical coupling, regulation of membrane depolarization
**WBGene00006749**	protein catabolic process, ion transmembrane transport	FBgn0085387	jump response, response to light stimulus, gap junction assembly, phototransduction, intercellular transport, cell communication by electrical coupling, regulation of membrane depolarization	FBgn0085387	jump response, response to light stimulus, gap junction assembly, phototransduction, intercellular transport, cell communication by electrical coupling, regulation of membrane depolarization

**WBGene00006765**	ion transport,transport, locomotion, regulation of oviposition, neuromuscular synaptic transmission, regulation of pharyngeal pumping, regulation of pharyngeal pumping, regulation of pharyngeal pumping	FBgn0032151	ion transport, response to insecticide, muscle cell homeostasis	FBgn0086778	jump response, synaptic transmission, ion transport, regulation of excitatory postsynaptic membrane potential, jump response, synaptic transmission, visual behavior
**WBGene00006774**	ion transport, transport, locomotion, regulation of oviposition, neuromuscular synaptic transmission	FBgn0015519	ion transport	FBgn0004118	ion transport
**WBGene00006791**	positive regulation of necrotic cell death, clathrin-mediated endocytosis	FBgn0038659	neurotransmitter secretion, synaptic vesicle endocytosis, regulation of synapse structure and activity, synaptic vesicle endocytosis, synaptic vesicle endocytosis, synaptic vesicle endocytosis, regulation of synapse structure and activity	FBgn0038659	neurotransmitter secretion, synaptic vesicle endocytosis, regulation of synapse structure and activity

**WBGene00006797**	ion transport, transport, locomotion, acetylcholine catabolic process, post-embryonic development, regulation of locomotion, ion transmembrane transport, regulation of oviposition, synaptic transmission, cholinergic, synaptic transmission, cholinergic, regulation of locomotion	FBgn0015519	ion transport	FBgn0004118	ion transport
**WBGene00007935**	regulation of transcription, DNA-dependent	FBgn0033667	regulation of transcription, DNA-dependent	FBgn0033668	regulation of transcription, DNA-dependent
**WBGene00007967**	oxidation-reduction process	FBgn0033978	oxidation-reduction process	FBgn0033121	oxidation-reduction process
**WBGene00008439**	dauer entry	FBgn0036196		FBgn0036196	
**WBGene00008810**	oxidation-reduction process	FBgn0033978	oxidation-reduction process	FBgn0033121	oxidation-reduction process
**WBGene00008819**	potassium ion transport, protein homooligomerization, ion transport, transmembrane transport	FBgn0003386	transmembrane transport, protein homooligomerization, potassium ion transport, sleep	FBgn0085395	protein homooligomerization, transmembrane transport, potassium ion transport
**WBGene00009218**	metabolic process, determination of adult lifespan	FBgn0050194		FBgn0021953	long-chain fatty acid transport, metabolic process, triglyceride homeostasis
**WBGene00009852**		FBgn0037519		FBgn0037521	phagocytosis, engulfment

**WBGene00010873**		FBgn0060296	calcium ion transport, sensory perception of pain, sensory perception of pain, response to mechanical stimulus, response to heat, detection of mechanical stimulus involved in sensory perception, response to heat, thermotaxis, feeding behavior, copulation, regulation of female receptivity, negative gravitaxis, behavioral response to pain, response to heat, regulation of heart contraction, sensory perception of pain, olfactory behavior, male courtship behavior, mechanosensory behavior	FBgn0260005	calcium ion transport, ion transport, transmembrane transport, response to humidity, sensory perception of sound
**WBGene00011269**	immune response	FBgn0259241	immune response	FBgn0259241	immune response
**WBGene00011675**	oxidation-reduction process	FBgn0033978	oxidation-reduction process	FBgn0033121	oxidation-reduction process
**WBGene00012464**	signal transduction	FBgn0037519		FBgn0037521	phagocytosis, engulfment
**WBGene00012467**	signal transduction	FBgn0037519		FBgn0037521	phagocytosis, engulfment
**WBGene00013043**	signal transduction	FBgn0037519		FBgn0037521	phagocytosis, engulfment
**WBGene00013542**	striated muscle myosin thick filament assembly	FBgn0031100		FBgn0031100	z
**WBGene00016918**	signal transduction	FBgn0039054	signal transduction	FBgn0039054	signal transduction
**WBGene00018060**		FBgn0037519		FBgn0037521	phagocytosis, engulfment

Supplementary Figures

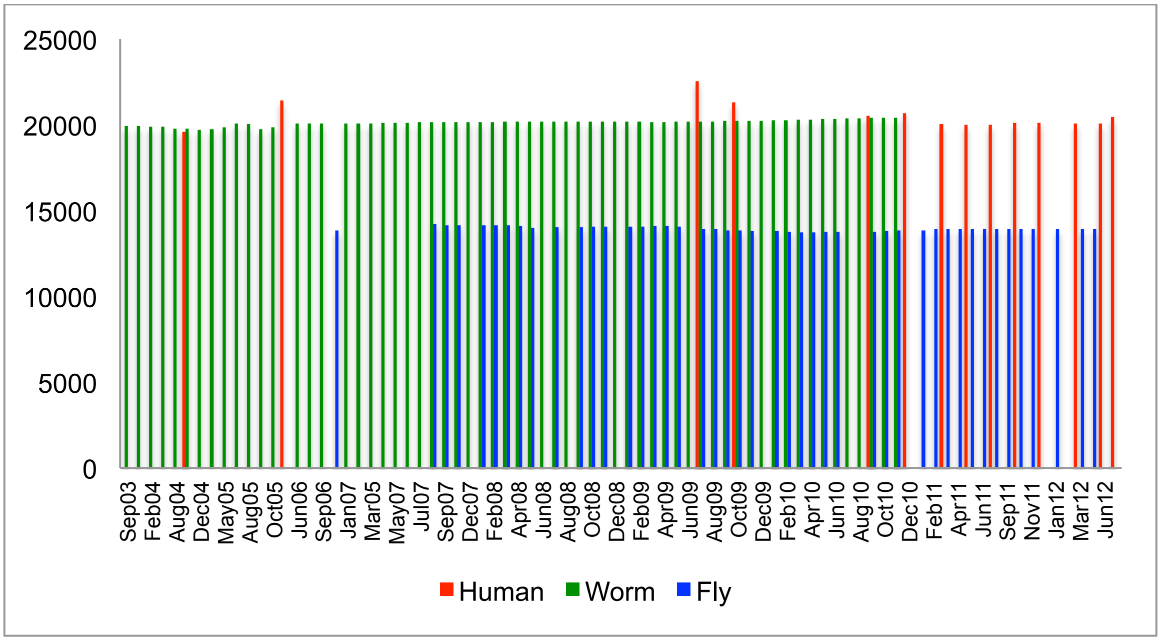

Fig. S1 - Figures Giving More Details on the "Comparative ENCODE RNA Resource".

Fig. S1a - Evolution of protein-coding gene annotation in human (red), worm (green), and fly (blue).

The number of protein-coding genes in each of the release is shown as barplots.

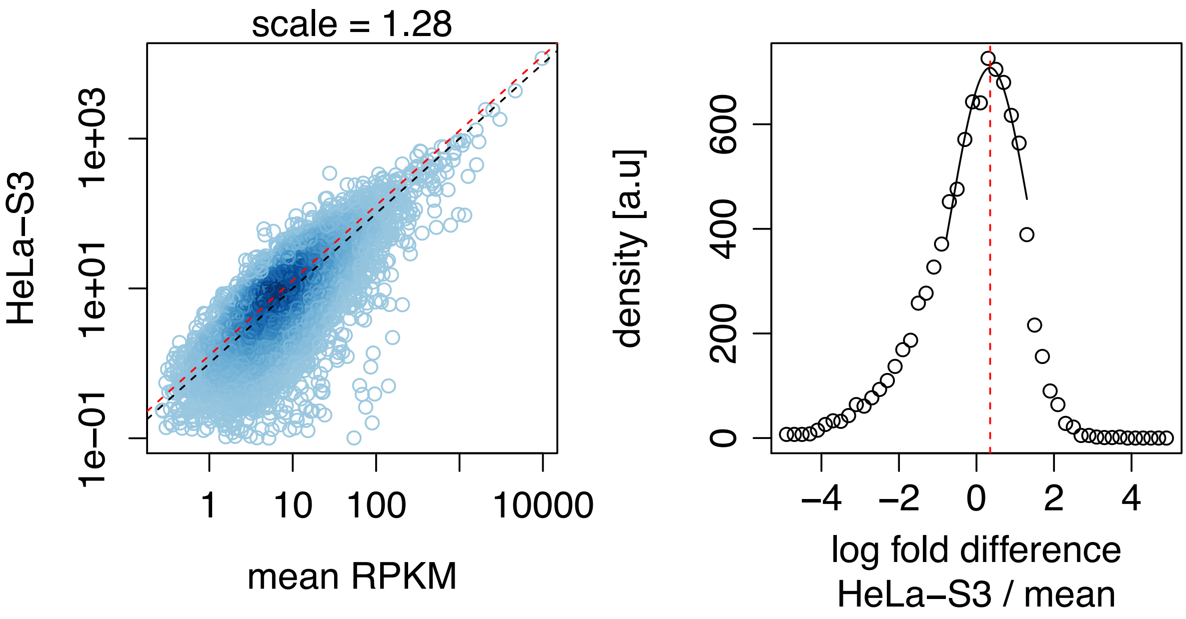

Fig. S1b - Gene expression in HeLa-S3 cells.

RPKM is plotted against average expression across all 19 cell types on a log-log scale for coding genes with RPKM>0.1 across all samples. Black line denotes the diagonal. Red line shows the best fit based on the log-fold change distribution. Right: Distribution of log-fold RPKM difference between HeLa-S3 cells and average across samples. The continuous line show a Gaussian fit.

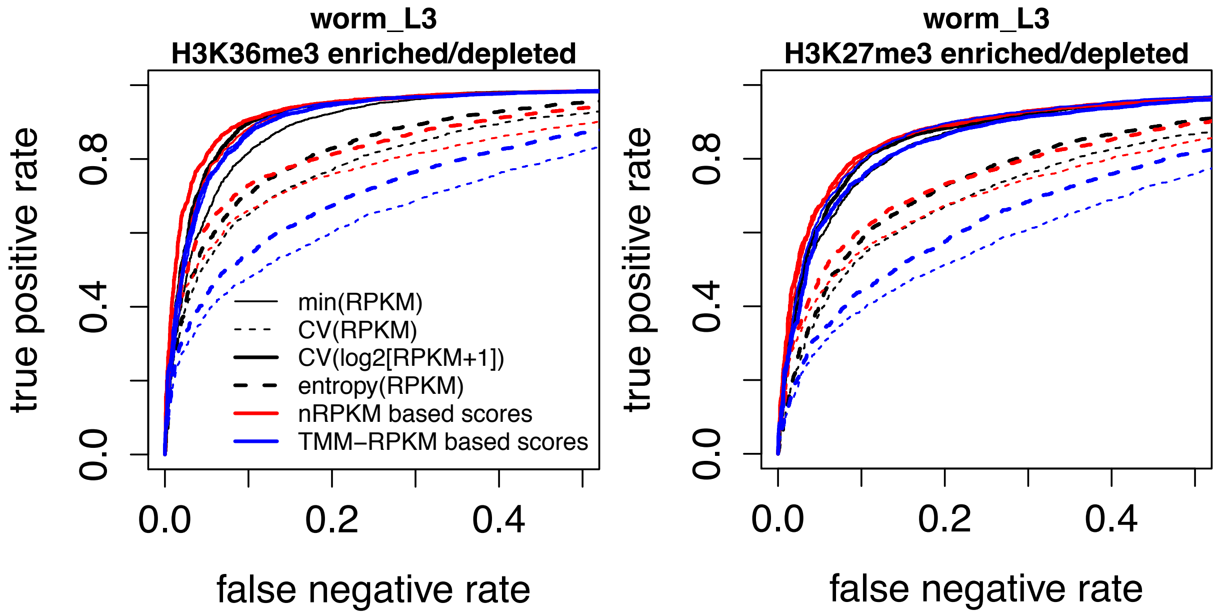

Fig. S1c - Broadly expression scores.

ROC curves for eight different expression scores based on broadly expressed gene assignments by H3K36me3 enrichment (left) or H3K27me3 depletion (right) in worm L3.

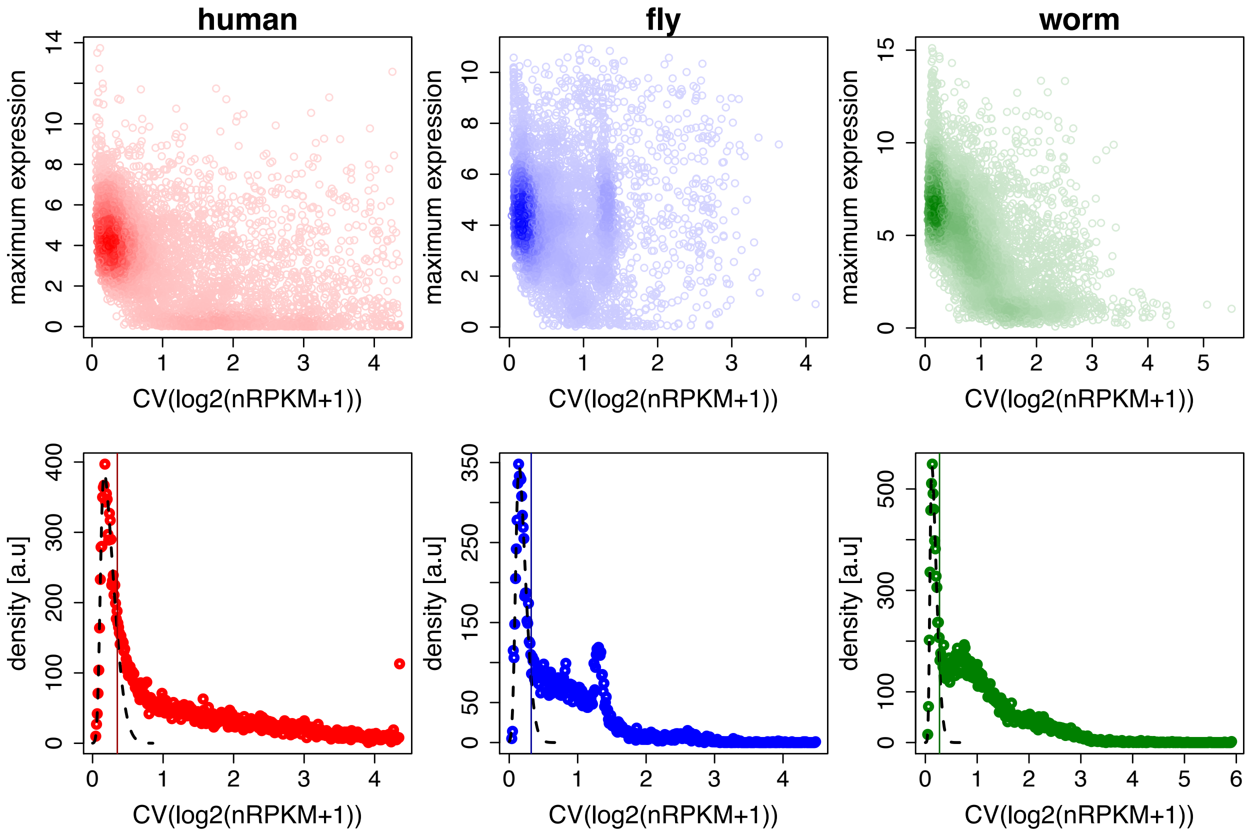

Fig. S1d - Maximum expression vs. variability score and distribution of variability score.

(Top) The maximum expression, given as $\log_2$(nRPKM+1), of each coding gene across all samples is plotted against the coefficient of variation of $\log_2$(nRPKM+1). (Bottom) Distribution of the coefficient of variation. A skew-normal fit around the peak is shown in black dashed line. The vertical colored lines denote the thresholds used to define broadly expressed genes in the three species.

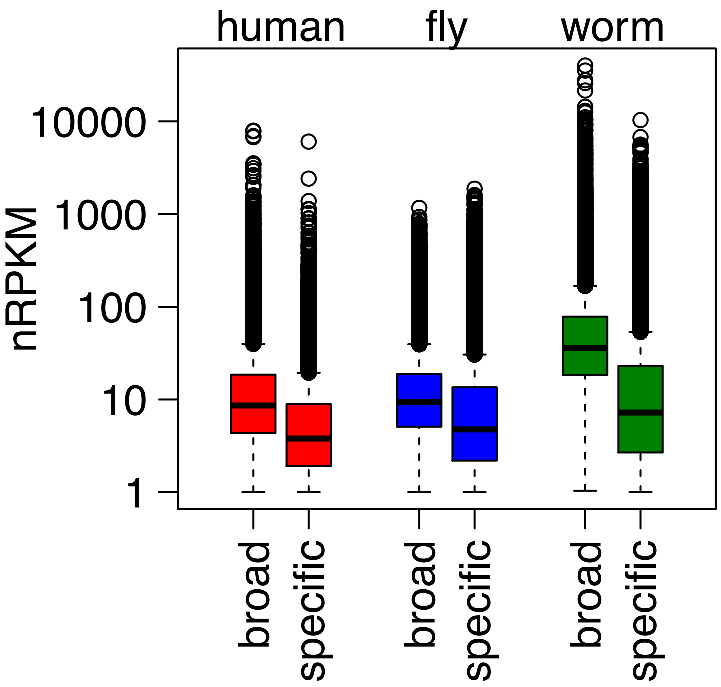

Fig. S1e - Normalized expression levels of broadly and specifically expressed protein-coding genes.

Boxplots for nRPKM values across all samples for broadly and specifically expressed coding genes with expression, nRPKM>1. The nRPKM values are not directly comparable across different species. For all three species, broadly expressed genes are more highly expressed than specifically expressed genes. This observation is independent of the precise threshold on nRPKM.

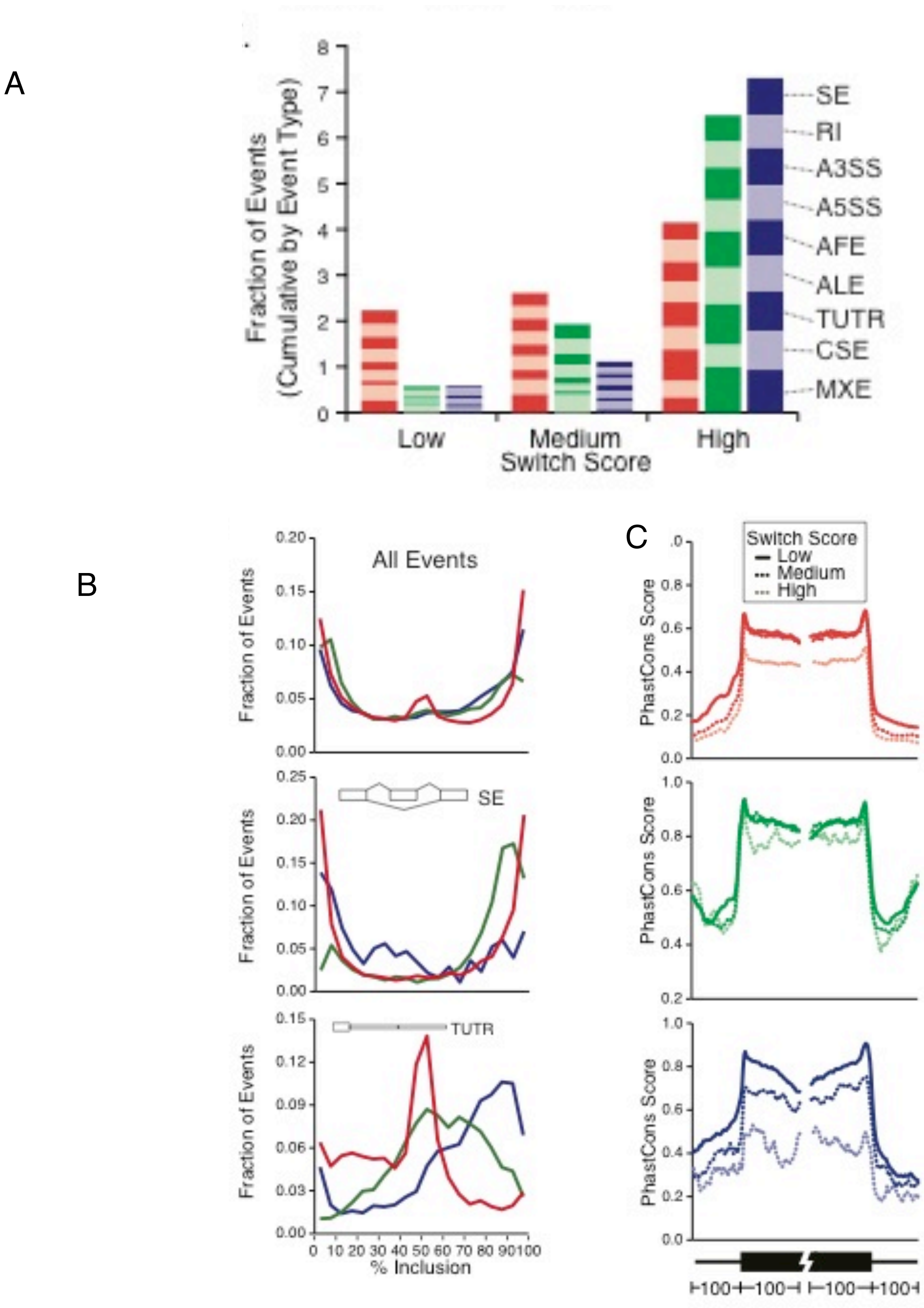

Fig. S1f - Comparison of alternative splicing.

(A) Percent inclusion for different events per species. (B) Switch scores per species. (See text for definition.) (C) Sequence conservation of intron-exon junctions in various switch-score groups in human, worms, and flies.

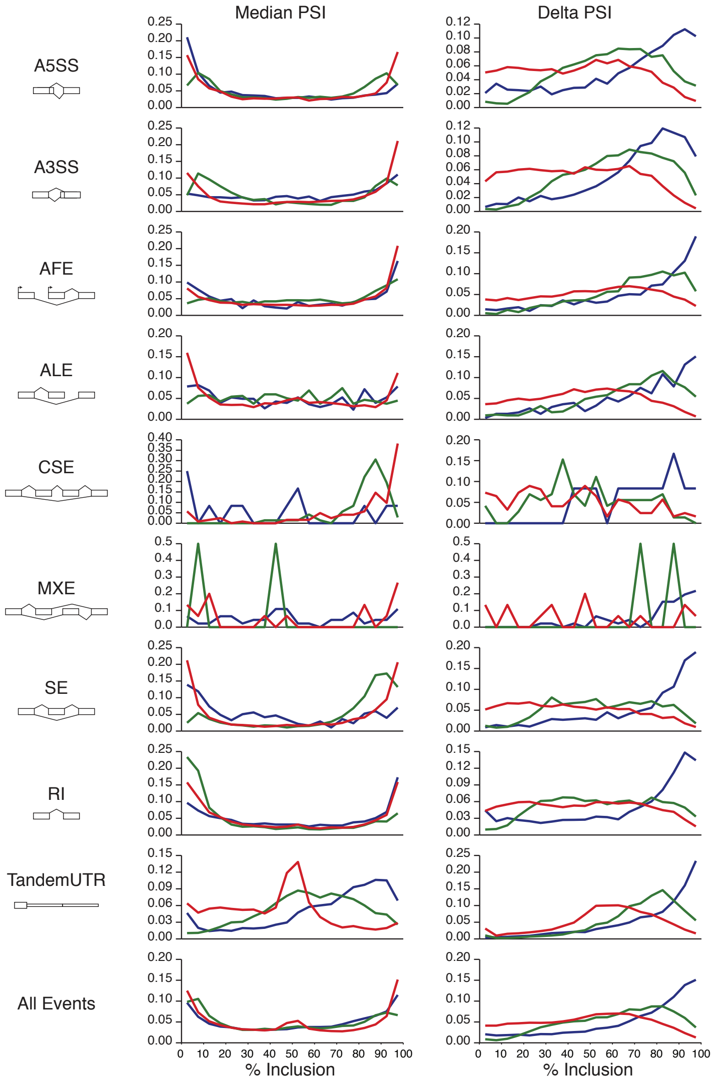

Fig. S1g - Distributions of splicing changes.

The median (left) and delta (right) percent inclusion (PSI) values for each class of splicing event in each species is plotted. The median PSI values are the median values among all samples in which the splicing event is expressed. The delta PSI values are calculated as the difference in the minimum and maximum PSI values in any sample. In each case, the values are plotted as the fraction of all events after quantitating the number of events in 5% bins. Human, worm and fly results are depicted in red, green, and blue, respectively. This figure only includes the protein-coding genes.

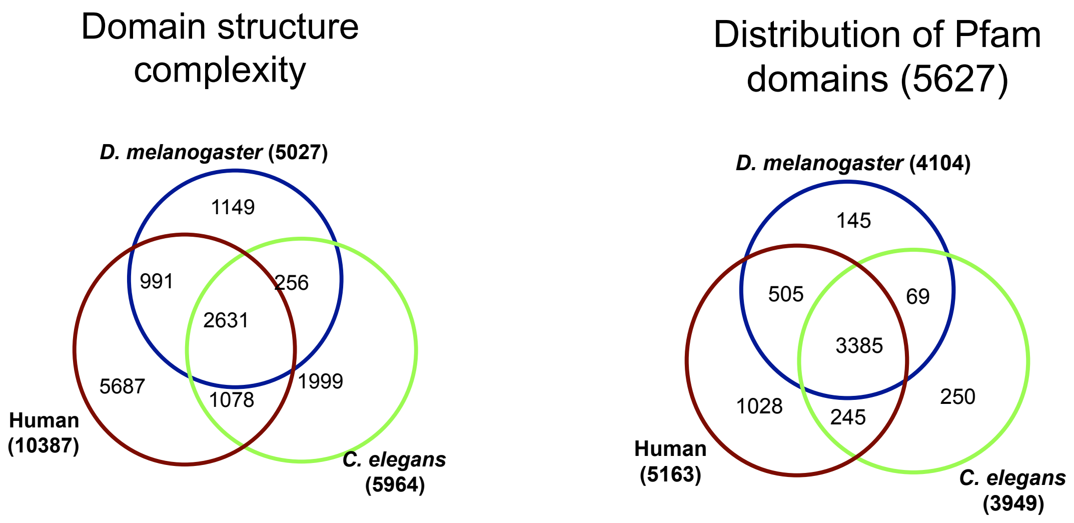

Fig. S1h - Venn diagrams of protein domains.

(Left) Distribution of protein domains using worm protein predictions with i-Evalue ≤ 0.001 - unique domains only. (Right) Distribution of Pfam domains using worm protein predictions with i-Evalue ≤ 0.001.

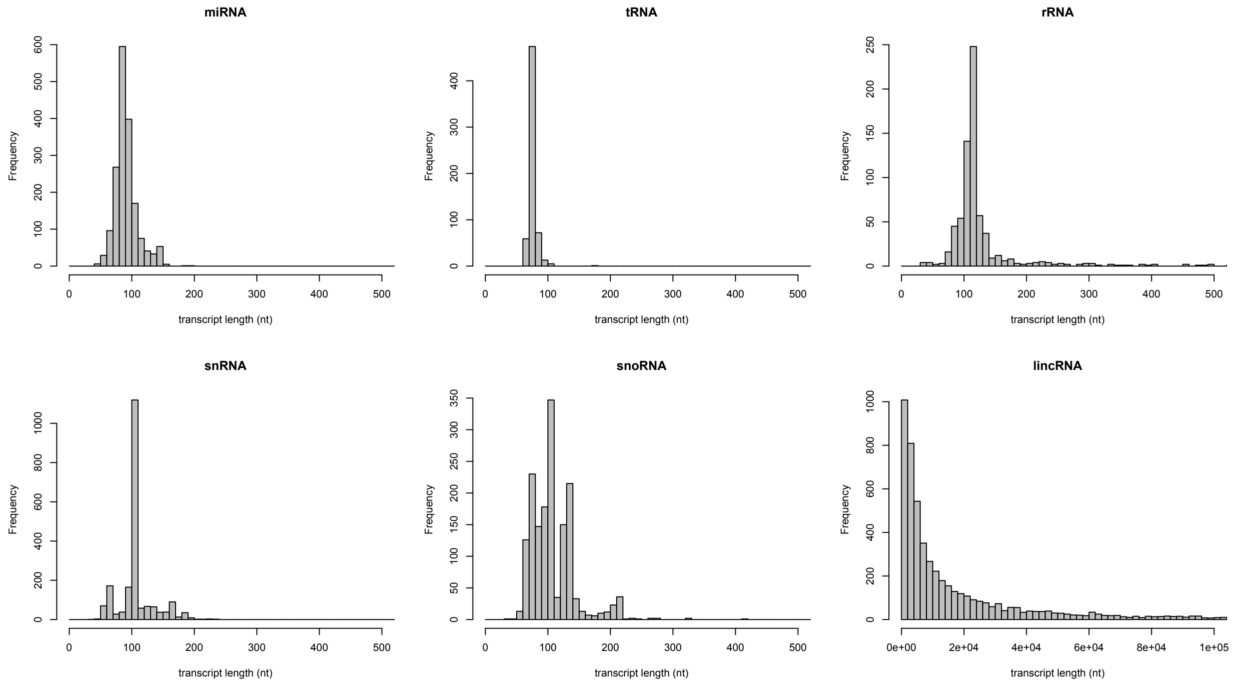

Fig. S2 – Figures Giving More Details on “ncRNAs & Non-canonical Transcription”.

Fig. S2a - Comparison of poly(A)+, total RNA and short RNA for detecting ncRNAs.

Fig. S2a1 - Distributions of lengths of various annotated human coding and non-coding RNAs.

Clearly the majority of annotated [pre-]miRNAs, tRNAs, rRNAs, snRNAs, and snoRNAs are <200nt in length and are therefore within the size range of RNA fragments that will be sequenced in a short-totalRNA-seq experiment but would likely be removed by the size selection performed for a long total- or poly(A)-RNA-seq experiment. However lincRNAs are sufficiently large to be retained by the long total- or poly(A)-RNA-seq fragment size selection.

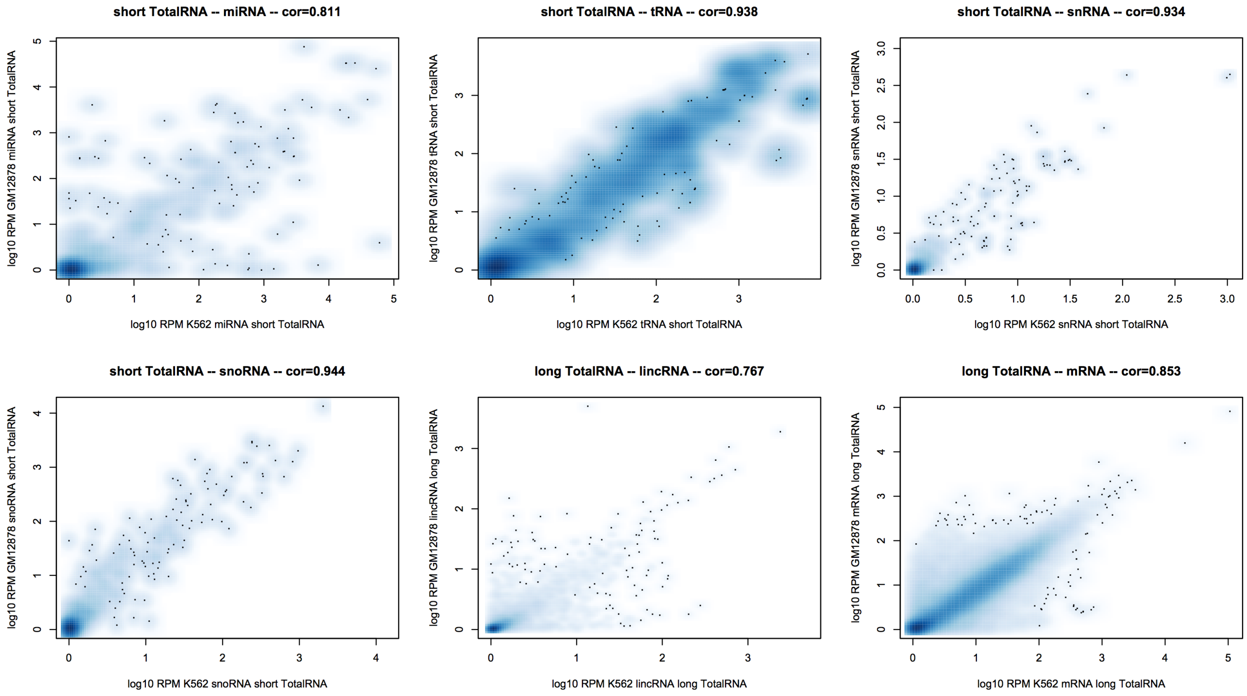

Fig. S2a2 - Biological variability.

Expression correlation of coding and non-coding RNAs between the GM12878 and K562 ENCODE (human) cell-lines highlights the large positive correlation between biologically distinct cell-types. Expression data, in log10 reads-per-million, and pearson correlations are shown, clockwise from top-left, for annotated human miRNA, tRNA, snRNA, snoRNA, lincRNA, and mRNA. Short-total RNA-seq data were used to compute miRNA, tRNA, snRNA, and snoRNA expressions, while long-total RNA-seq was used for lincRNA and mRNA expression quantification.

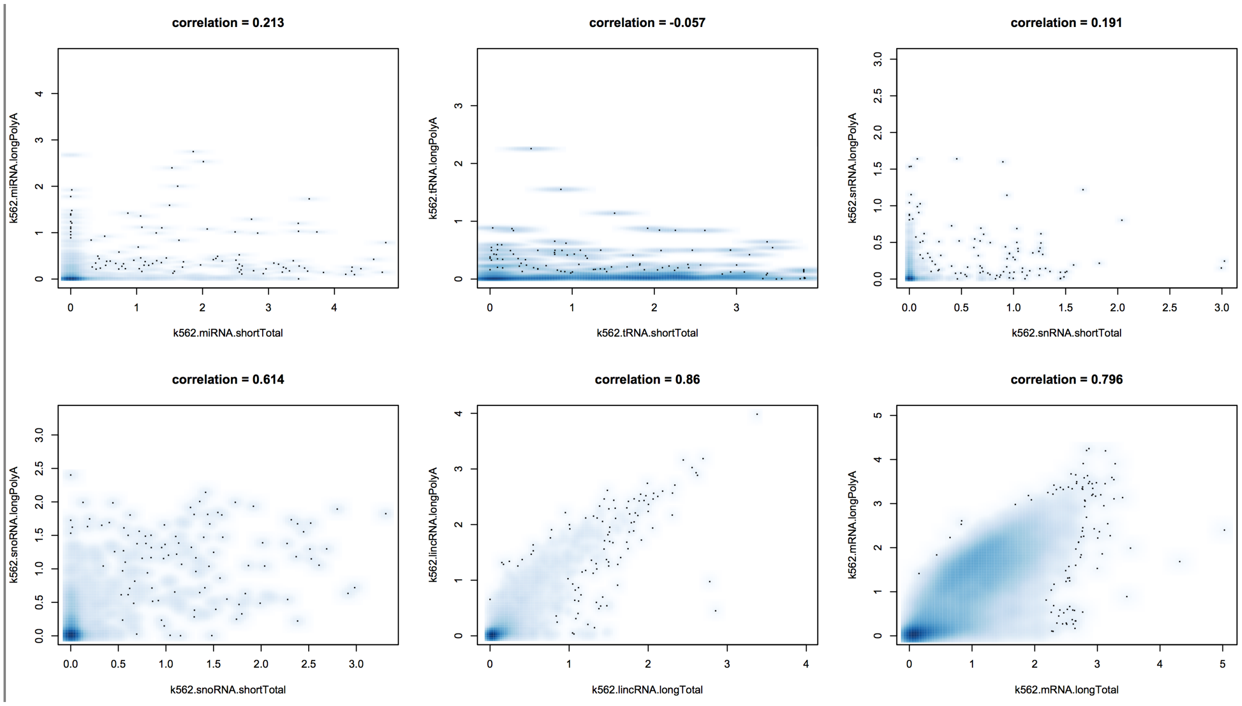

Fig. S2a3 - K562 RNA sample preparation comparison.

Effect of poly(A) RNA purification, during sample-prep, on ability to detect coding and non-coding RNAs in the K562 ENCODE (human) cell-line. Poly(A) RNA-seq data, obtained for the majority of the modENCODE samples, perform poorly compared to short-total RNA-seq at detecting miRNAs, tRNAs, snRNAs and snoRNAs, but are much better able to detect lincRNAs and mRNAs.

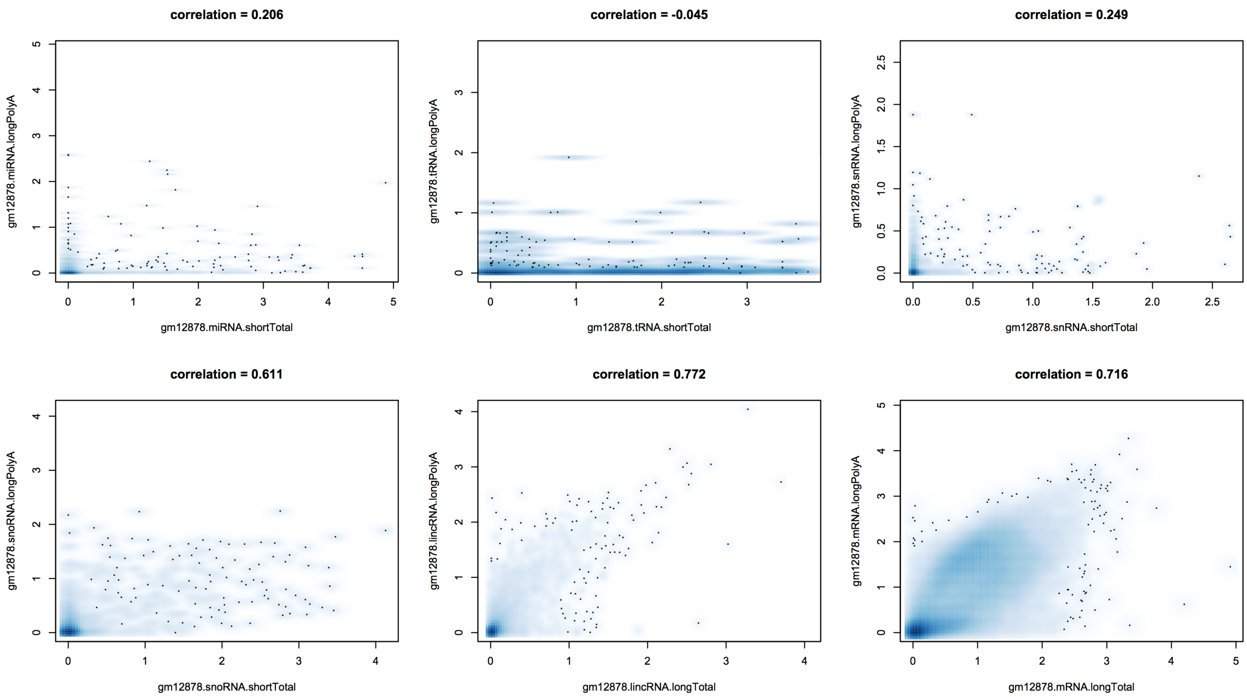

Fig. S2a4 - GM12878 RNA sample preparation comparison.

Effect of poly(A) RNA purification, during sample-prep, on ability to detect coding and non-coding RNAs in the GM12878 ENCODE (human) cell-line. Poly(A) RNA-seq data, obtained for the majority of the modENCODE samples, perform poorly compared to short-total RNA-seq at detecting miRNAs, tRNAs, snRNAs and snoRNAs, but are much better able to detect lincRNAs, and mRNAs.

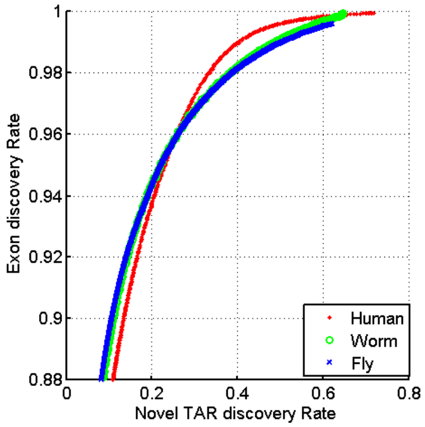

Fig. S2b - ncRNAs and non-canonical transcription.

Summary of the number of annotated ncRNAs, supervised ncRNA predictions and the estimates of the amount of non-canonical transcription in each of the three genomes. (B) ROC-like plots for predicting the amount of non-canonical transcription using the sets of expressed annotations for each organism as a gold standard. The red (fly), green (worm), and blue (human) distributions show the exon discovery rate and novel TAR discovery rate for the full set of parameters using a minimum-run/maximum-gap/threshold algorithm.

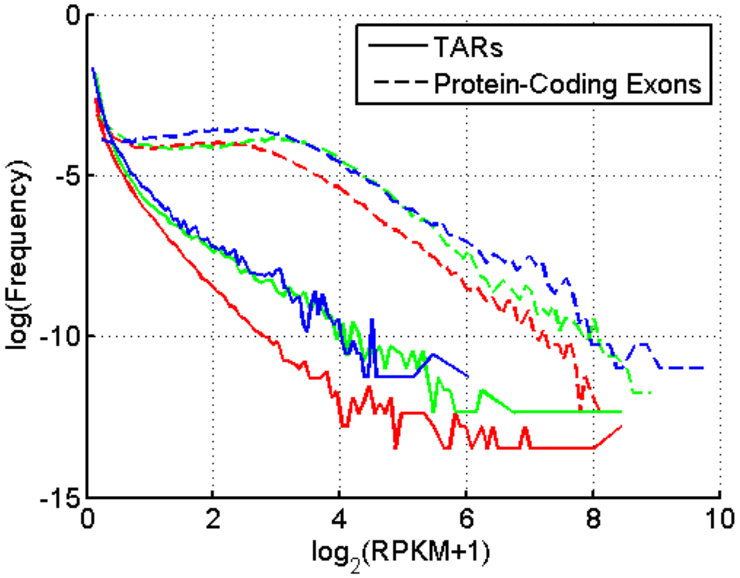

Fig. S2c - Expression distributions of the TARs and exons in all three species.

RPKM distribution for annotated protein coding exons (dashed) and relaxed TARs (solid) for human (red), worm (green), and fly (blue).

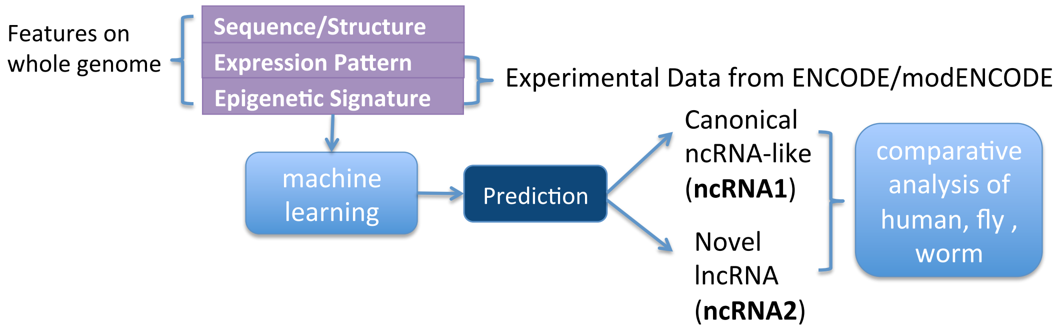

Fig. S2d - Supervised ncRNA predictions.

Fig. S2d1 - Supervised ncRNA prediction pipeline.

A machine learning model integrated sequence/structure features, expression features and epigenetic features from ENCODE/modENCODE, predicted two types of ncRNAs, canonical ncRNA like (ncRNA1) and novel lncRNA (ncRNA2), comparative analysis of these ncRNA was conducted in these three species.

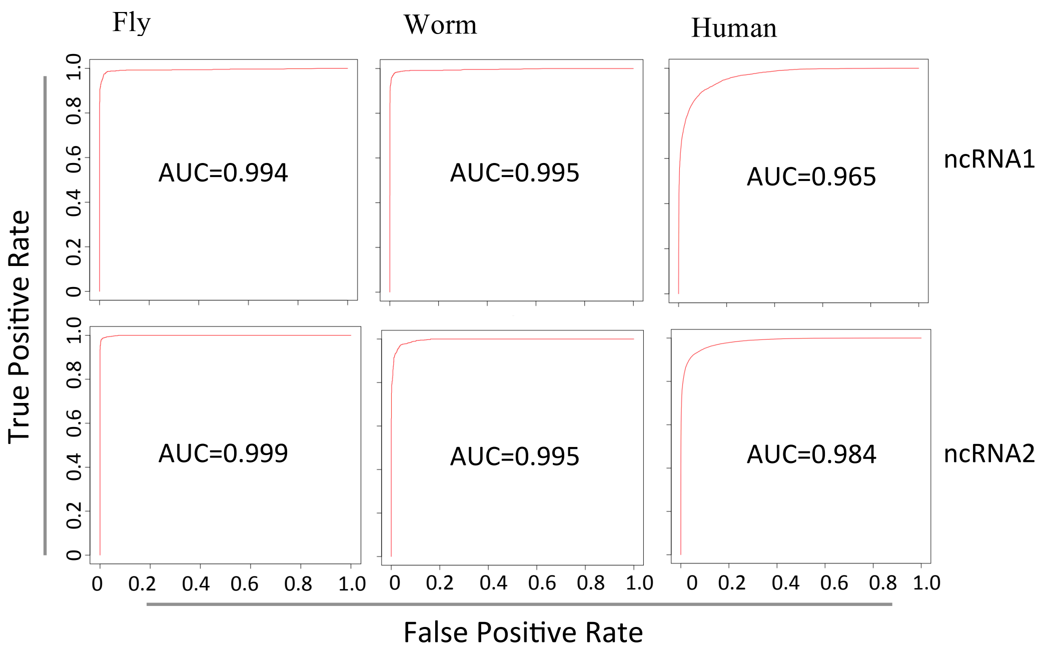

Fig. S2d2 - Model performance in fly, worm and human.

ROC curve plot showing supervised ncRNA prediction performance on three species.

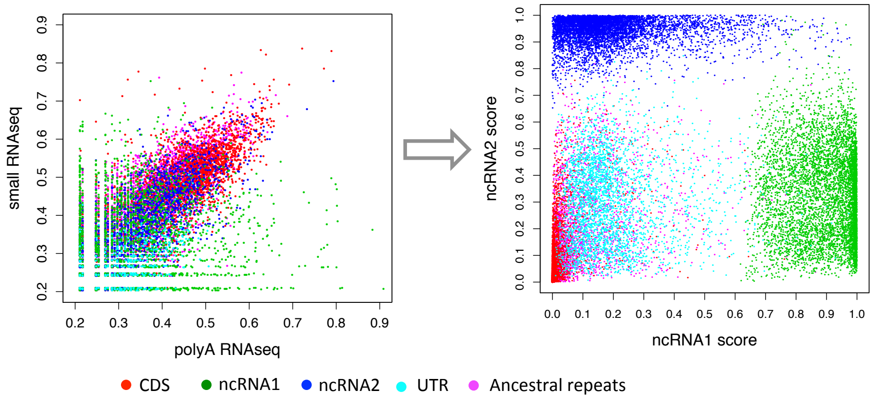

Fig. S2d3 - Performance of supervised ncRNA predictions in human.

Scatter plot show that two features cannot separate biotypes well, but when integrated all features, all biotypes separate clearly from each other.

	brain	heart	kidney	liver	lung	muscle	spleen	testis
1	17	N	N	N	N	N	N	256
2	93	N	N	N	24	N	19	N
3	7,025	435	33,720	426	710	4,908	10,461	772
4	202,998	35,494	76,493	4,746	46,161	30,018	43,151	23,159
5	283	26	3,071	93	153	1,664	7,630	99
6	204,446	37,336	50,259	2,800	26,560	24,554	49,587	38,736
7	N	N	N	N	N	N	N	25
8	68	80	N	N	21	N	N	N
9	214	N	213	455	250	N	265	326
10	17	N	N	N	N	N	N	266
11	29	N	N	N	N	N	N	511
12	178,374	21,277	49,130	2,390	21,255	22,731	15,103	12,487
13	97	203	357	177	212	N	122	1,480
14	141	96	16	N	169	N	86	N
15	44	N	N	N	16	N	52	N
16	129	N	N	N	N	N	N	N
17	14,143	1,053	50,092	1,257	2,378	6,252	12,963	701
18	4,501	820	17,004	90	885	269	133	456
19	710	635	744	485	1,620	71	493	248
20	366,428	45,363	92,318	4,911	44,200	44,507	7,649	25,616
21	11,814	4,074	4,511	292	7,224	1,148	4,457	2,449
22	18,095	6,480	3,944	2,070	5,956	2,301	5,946	2,184
23	226	14	391	12	96	191	7,494	75
24	11	20,791	39,212	19,810	24,003	8,409	26,021	13,183
25	28,547	11,739	16,898	3,708	11,034	5,661	10,870	5,605

Fig. S2d4 – RT-PCRs and amplicons sequencing of ncRNA candidates in different human tissues.

25 ncRNA candidates were selected from previous supervised ncRNA predictions in conserved regions of chromosome 1. All of them are expressed in at least one tissue (covered by at least 10 reads). The numbers of sequencing reads overlapping the amplicons in each tissue are shown in the table (white: low coverage, red: high coverage, "N": no reads).

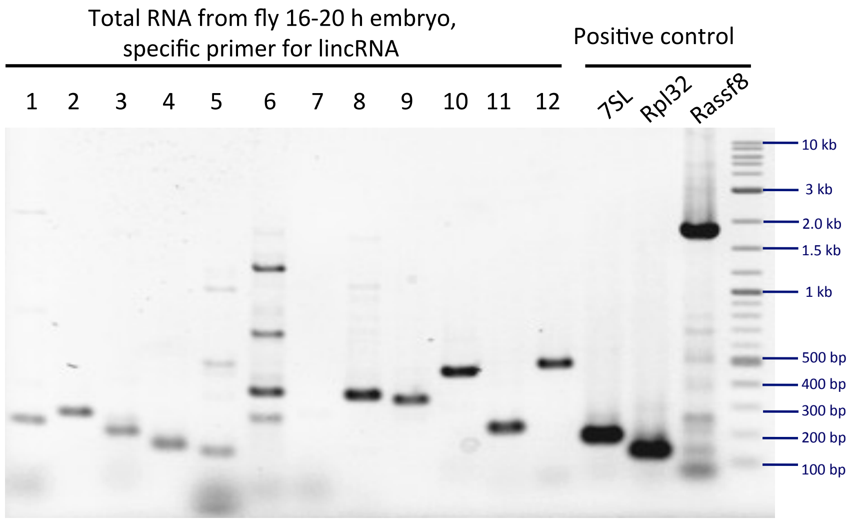

Fig. S2d5 - RT-PCR validation in Fly embryo.

An electrophoresis map showed that 10 ncRNAs were validated in 12 selected candidates. Total RNA was extracted from 16-20h embryo stage, random hexamer primer was used for cDNA synthesis. 7SL, Rpl32 and Rassf8 are positive controls, a clear ~1.8kb band for Rassf8 denotes the amplified cDNA, no 2.2kb (genomic DNA length for the same primer of Rassf8) band amplified clarify no genomic DNA contamination in cDNA template.

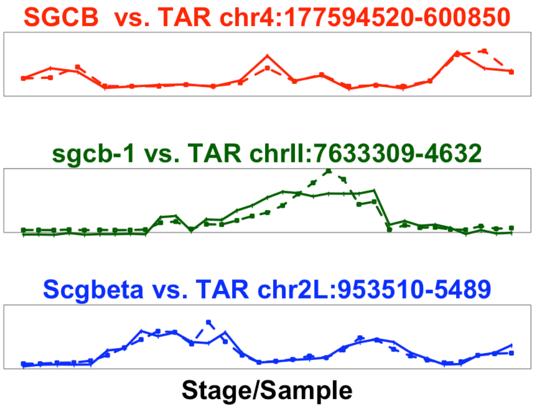

Fig. S2e - Further example of highly correlated TAR triplet with 1-1-1 orthologous genes.

As contrast to the anti-correlated TARs in Figure ED5, we identified three TARs that are highly correlated with the expression of the orthologous genes, SGCB in human (TAR chr4:177594520-600850, r=0.93, p<8e-9), sgcb-1 in worm (TAR chrII:7633309-4632, r=0.96, p<3e-16), and Scgbeta in fly (TAR chr2L:953510-5489, r=0.95, p<5e-16). Also, those TARs do not overlap with introns of the three orthologous genes.

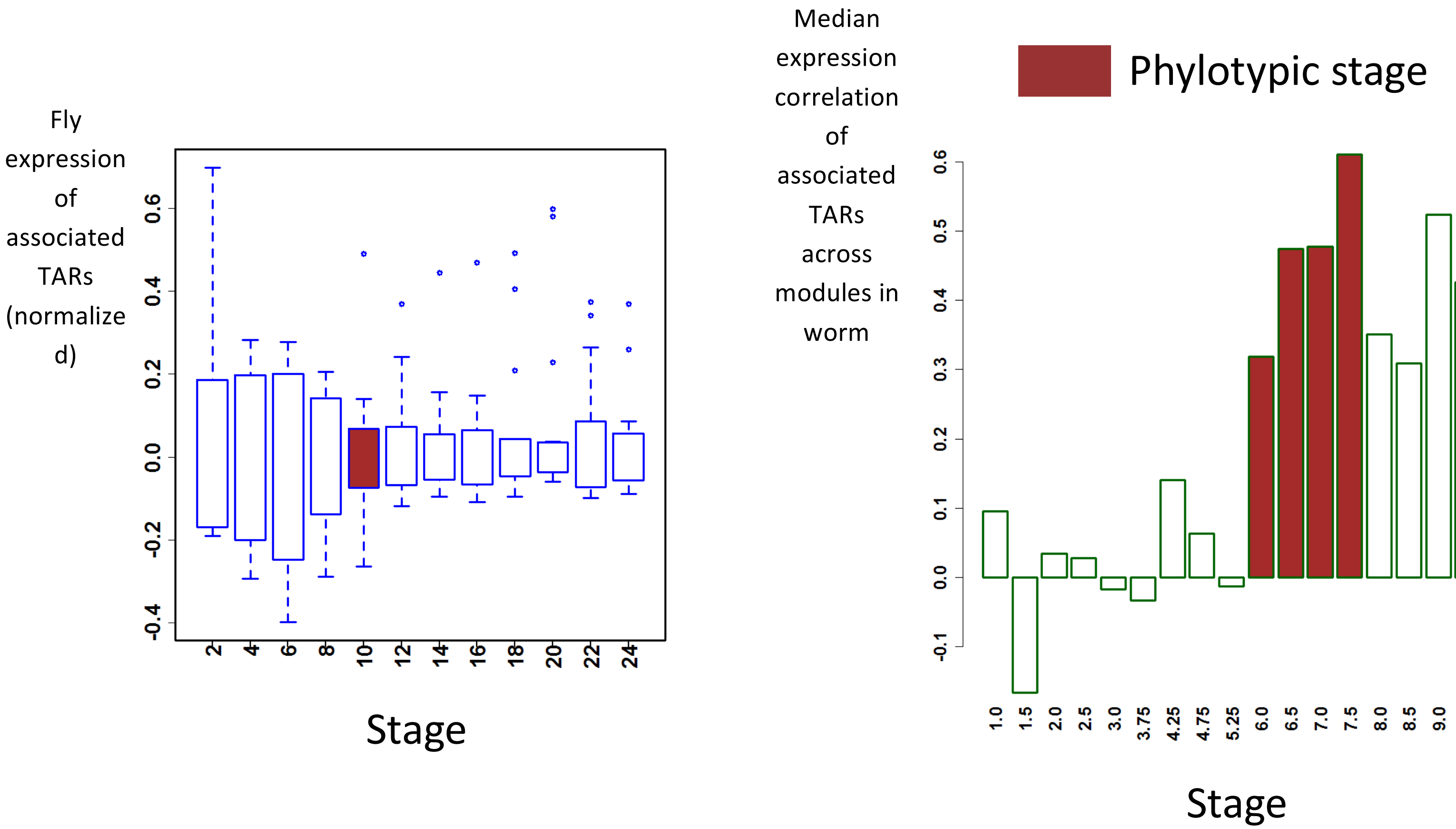

Fig. S2f - Hourglass behavior of TARs assigned to modules.
Parallel to Fig. 2 and ED6C we plot the corresponding hourglass behavior for TARs associated with modules (Table S3). We plot expression differences for fly and correlations for worm for these TARs.

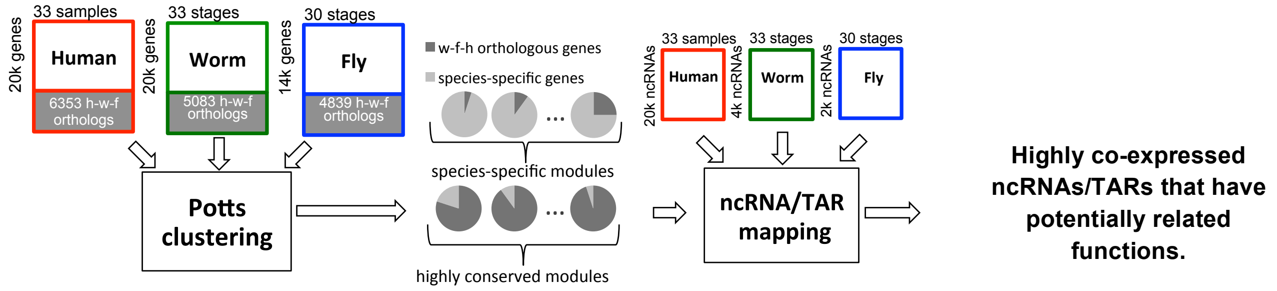

Fig. S3 - Figures Giving More Details on "Expression Clustering & Stage Alignment".

Fig. S3a - Clustering procedure.

The flowchart illustrates the method used to cluster genes across three species into co-expression modules via Potts model, and to map highly co-expressed ncRNAs/TARs to the modules.

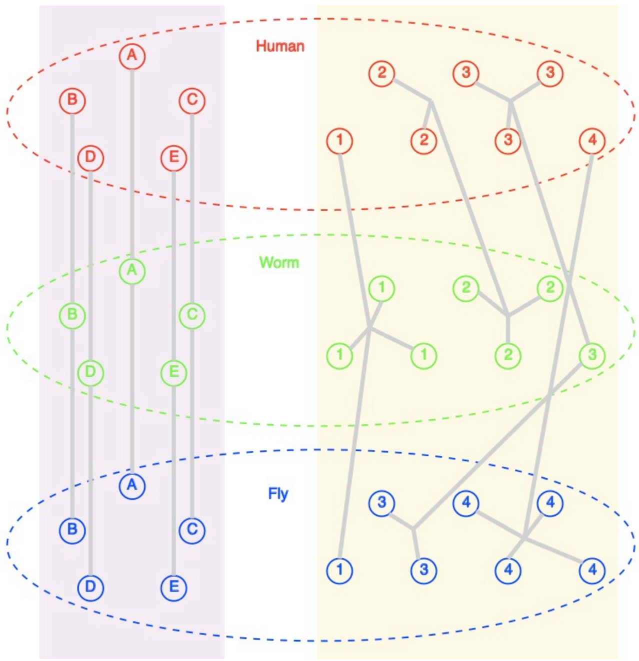

Fig. S3b – Schematic illustration of Potts modules conserved across three species but with different gene duplication rate.

Small circles with labels are genes from the three species: human, worm and fly, and their colors denote species origins. The labels inside small circle symbolize ortholog group IDs. Genes from the same ortholog group are connected via grey lines. On the left, one module is composed of the same five genes (A-E, which are 1-1-1 human-worm-fly orthologs) in three different species. In the other module on the right, member genes (from 4 ortholog groups: 1-4) in different species are not exactly the same. The difference mainly results from various gene duplications in different species.

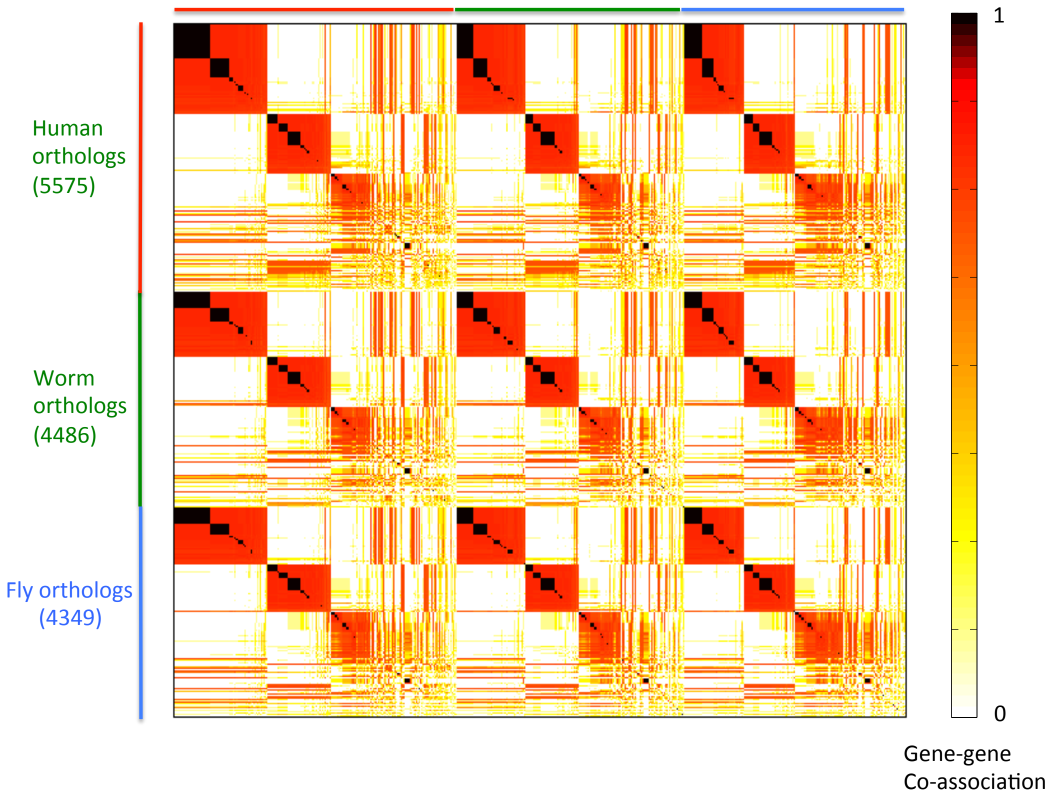

Fig. S3c - Clustering of orthologs in 3 species.

A set of conserved genes (5,575 human genes, 4,486 worm genes, and 4,349 fly genes) that form orthologs in the three species are clustered by our Potts model. The map shows a set of conserved modules. Genes from human, worm and fly are clustered into same modules as shown by the off-diagonal elements.

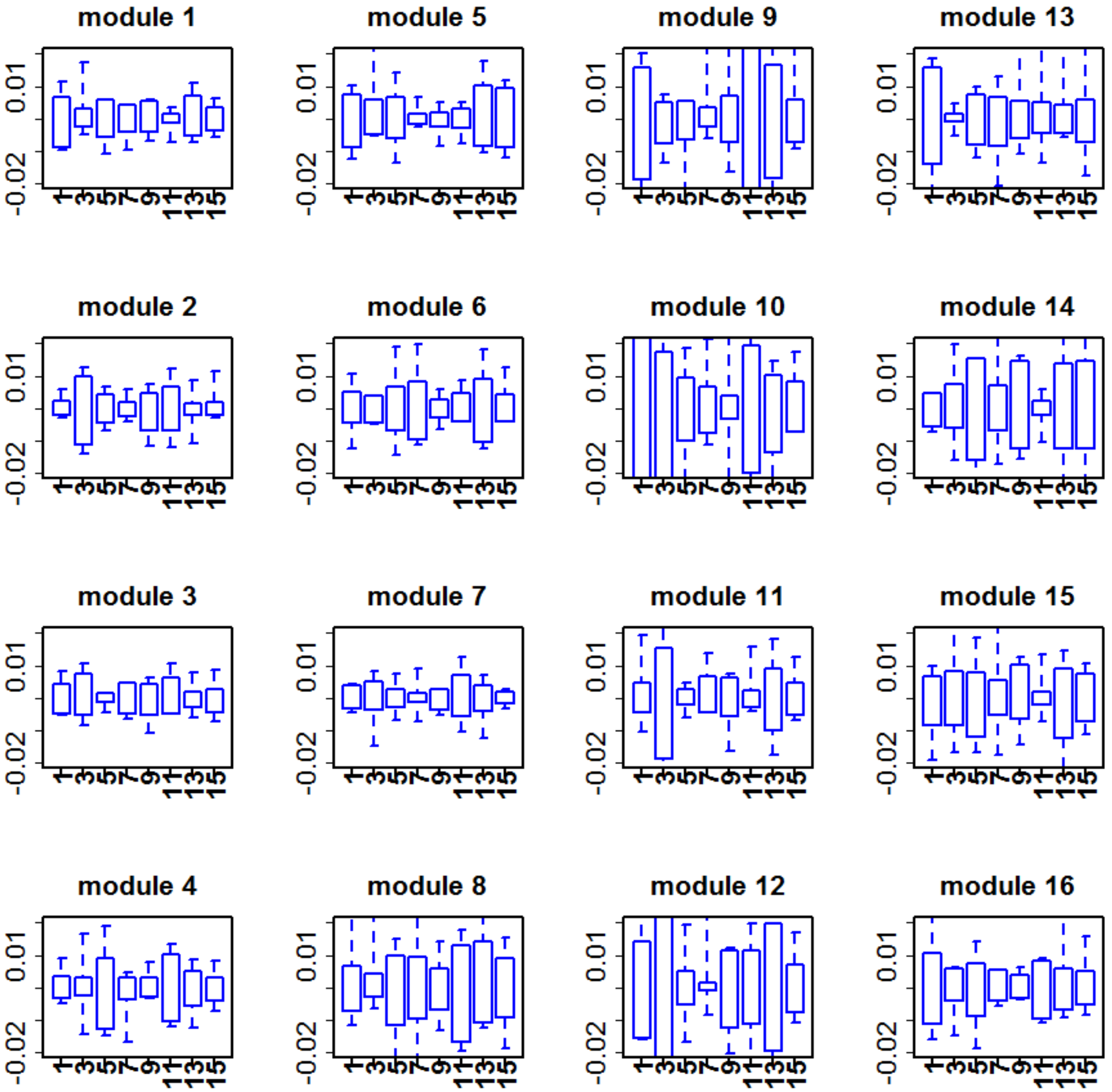

Fig. S3d - Hourglass patterns of 16 conserved modules.

There are 12 out of 16 modules that display hourglass patterns, i.e., the modular expression divergences decrease during the middle embryo stages, and increase afterwards. The x-axis is the middle time point of two-hour period. The y-axis represents the Median-centered modular expression levels.

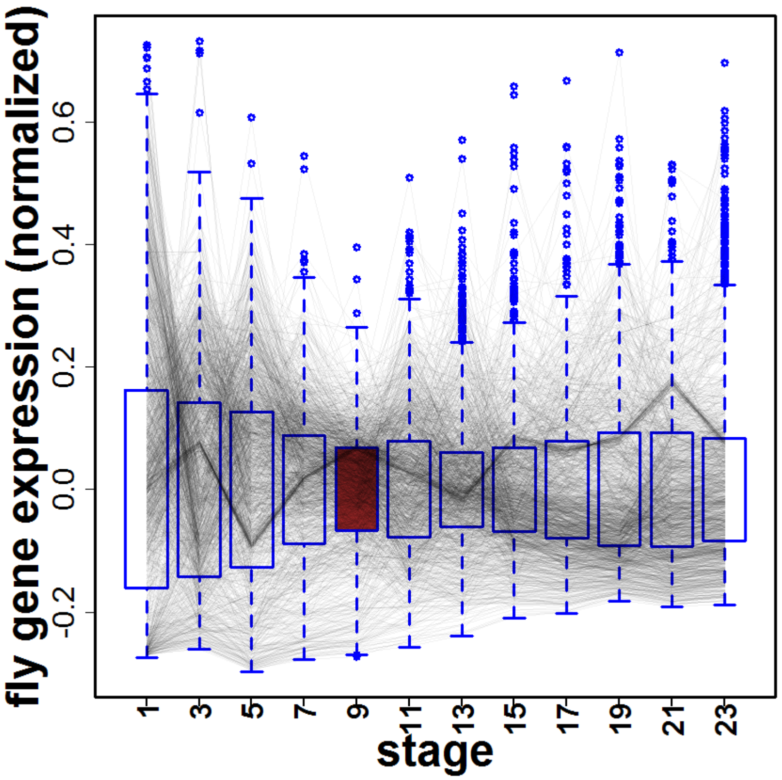

Fig. S3e – Hourglass pattern of fly gene expressions, and schematic visualization of worm modular expressions.

Fig. S3e1 - Divergence of fly gene expression across developmental stages.

The expression differences for genes in 16 conserved modules show an hourglass pattern. Each gene's expression level across stages is normalized so that its vector norm is one. The y-axis is Median-center normalized gene expression levels (blue: quartiles, grey: normalized gene expression levels). The x-axis is the middle time point of two-hour period. The fly phylotypic stage (8-10 hours) is highlighted by brown.

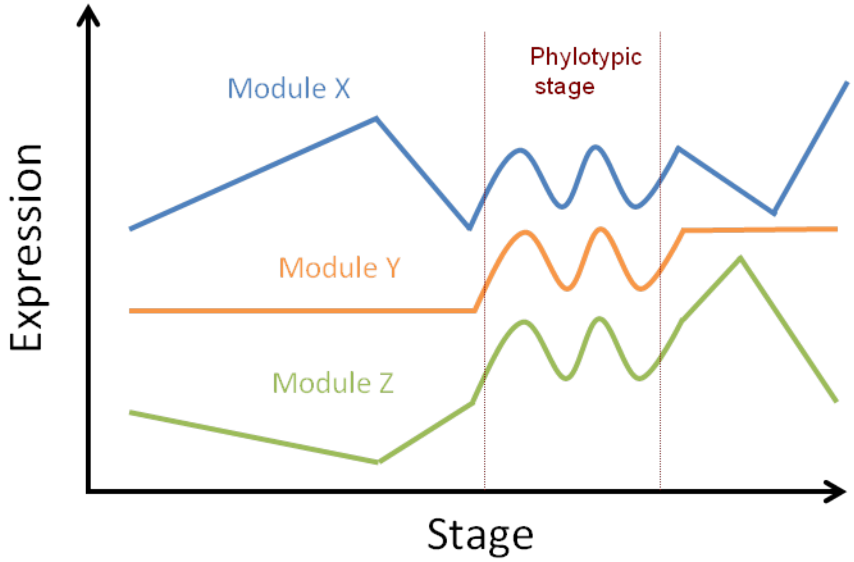

Fig. S3e2 - Schematic of correlated modular expressions during phylotypic stage in worm

This schematic shows that worm modular expressions (e.g., Modules X, Y and Z) correlate during the phylotypic stage, but do not correlate before or after the phylotypic stage, which is parallel to Fig. ED6c.

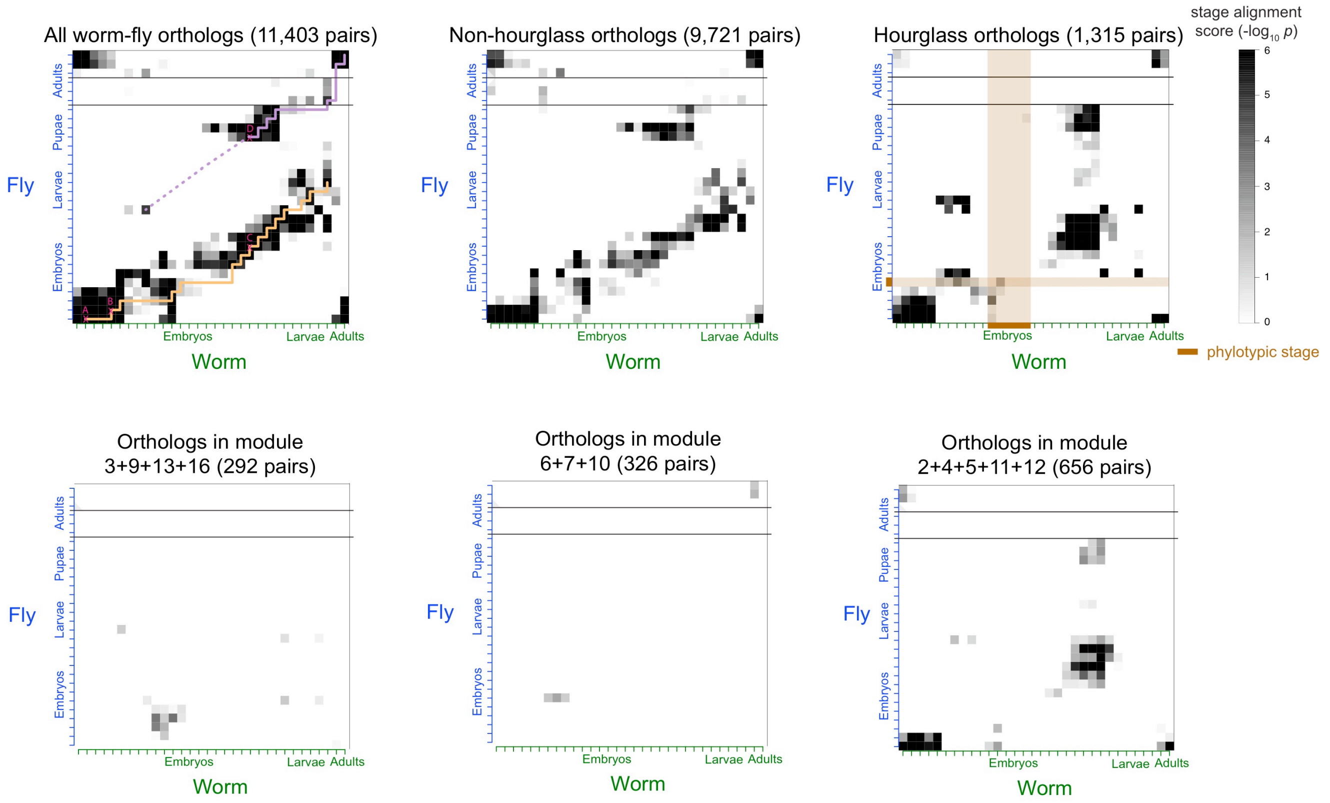

Fig. S3f - Worm-Fly Developmental Stage Alignment.

Fig. S3f1 - Alignment of worm and fly developmental stages based on worm-fly orthologs.

(Top left) An alignment of worm and fly developmental stages based on all worm-fly orthologs (11,403 pairs, including one-to-one, one-to-many, many-to-many pairs). (Top middle) An alignment of worm and fly developmental stages based on 9,721 worm-fly ortholog pairs that do not involve hourglass genes. (Top right) An alignment of worm and fly developmental stages based on 1,315 worm-fly ortholog pairs that are hourglass genes in both species. (Bottom left) An alignment of worm and fly developmental stages based on 292 worm-fly ortholog pairs in modules 3, 9, 13 and 16. (Bottom middle) An alignment of worm and fly developmental stages based on 326 worm-fly ortholog pairs in modules 6, 7 and 10. (Bottom right) An alignment of worm and fly developmental stages based on 1,184 worm-fly ortholog pairs in modules 2, 4, 5, 11, and 12.

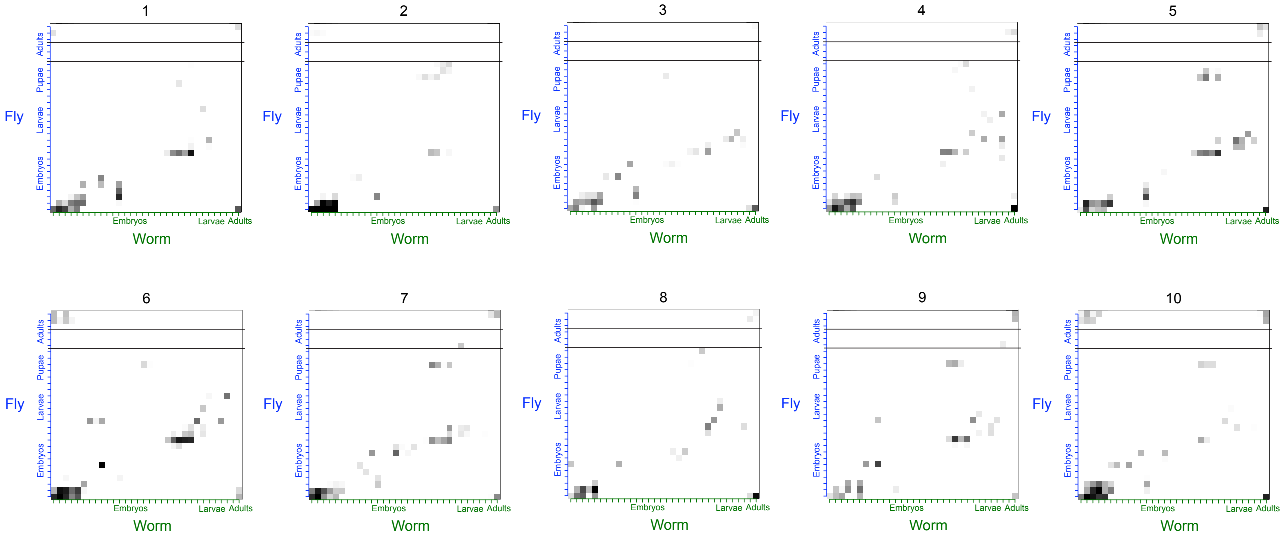

Fig. S3f2 - Alignment simulations by subsampling 1,315 ortholog pairs from all worm-fly orthologs.

Here presents the result of 10 simulations where each shows an alignment of worm and fly developmental stages based on randomly sampled 1,315 worm-fly ortholog pairs from the total of 11,403 pairs. Compared to these simulations, the top right heatmap in Fig. S3f1 shows a much stronger alignment based on the 1,315 worm-fly ortholog pairs that are hourglass genes.

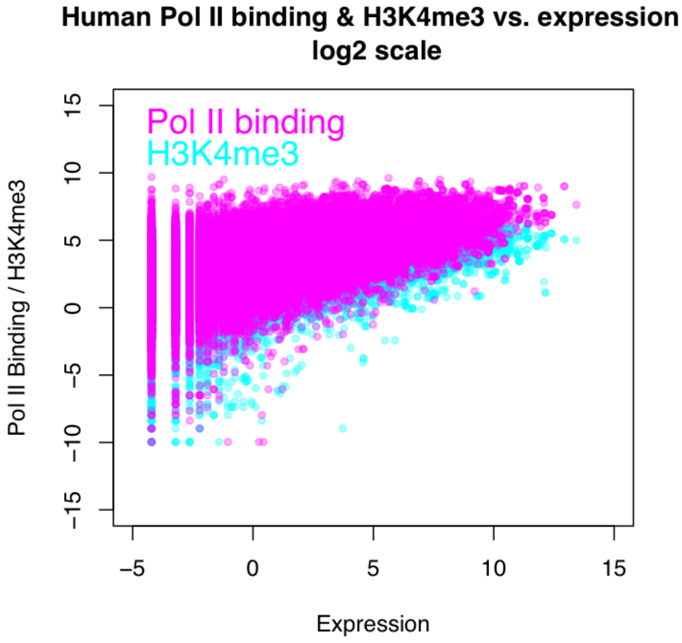

Fig. S4 – Figures Giving More Details on “Modeling Gene Expression with Chromatin and TFs”.

Fig. S4a - H3K4me3 vs. expression.

We show the correlation of gene expression levels with both the amount of Pol II and H3K4me3 marks proximal to the genes (bin centered on the TSS) in worm, human, and fly in this supplementary exhibit. Pol II and expression data for worm and fly are measured in the early embryo stage, while the human data was measured in the H1 embryo stem cell line. H3K4me3 histone mark data are all from embryo stages.

Fig. S4a1 - Human scatterplot.

Spearman’s correlation of Pol II binding and gene expression is 0.67. Spearman’s correlation of H3K4me3 and gene expression is 0.43.

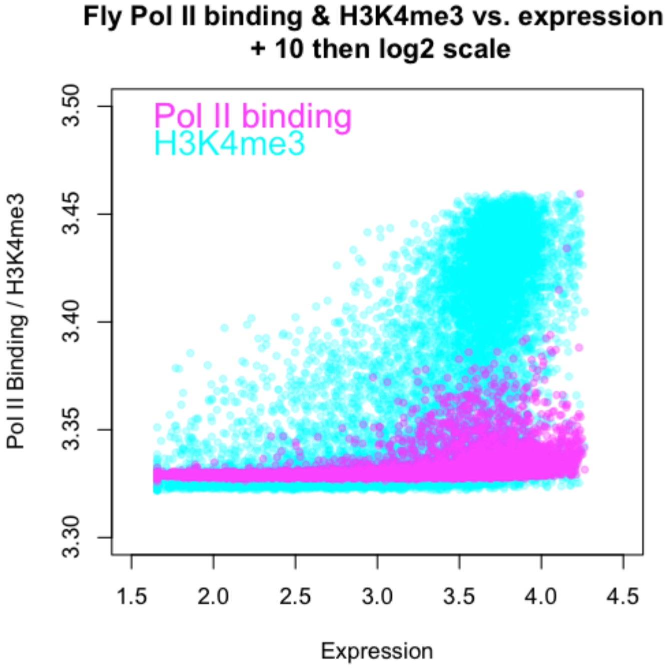

Fig. S4a2 - Fly scatterplot.

Spearman's correlation of Pol II binding and gene expression is 0.62. Spearman's correlation of H3K4me3 and gene expression is 0.77.

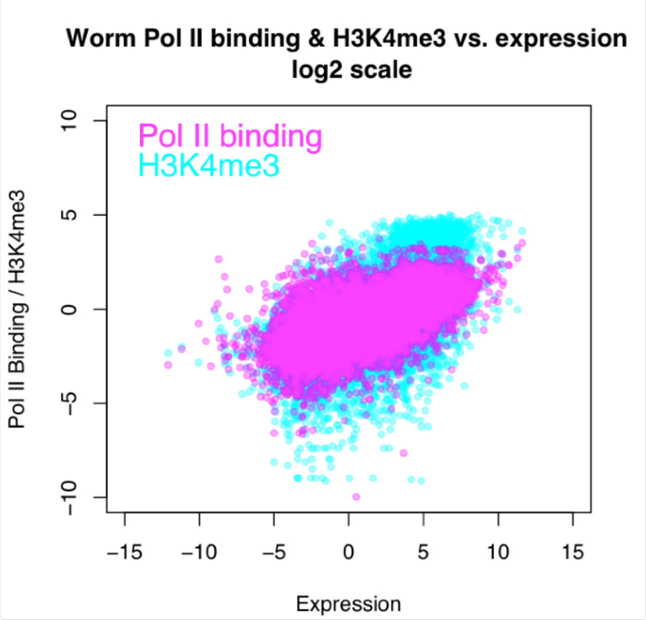

Fig. S4a3 - worm scatterplot.

Spearman's correlation of Pol II binding and gene expression is 0.64. Spearman's correlation of H3K4me3 and gene expression is 0.58.

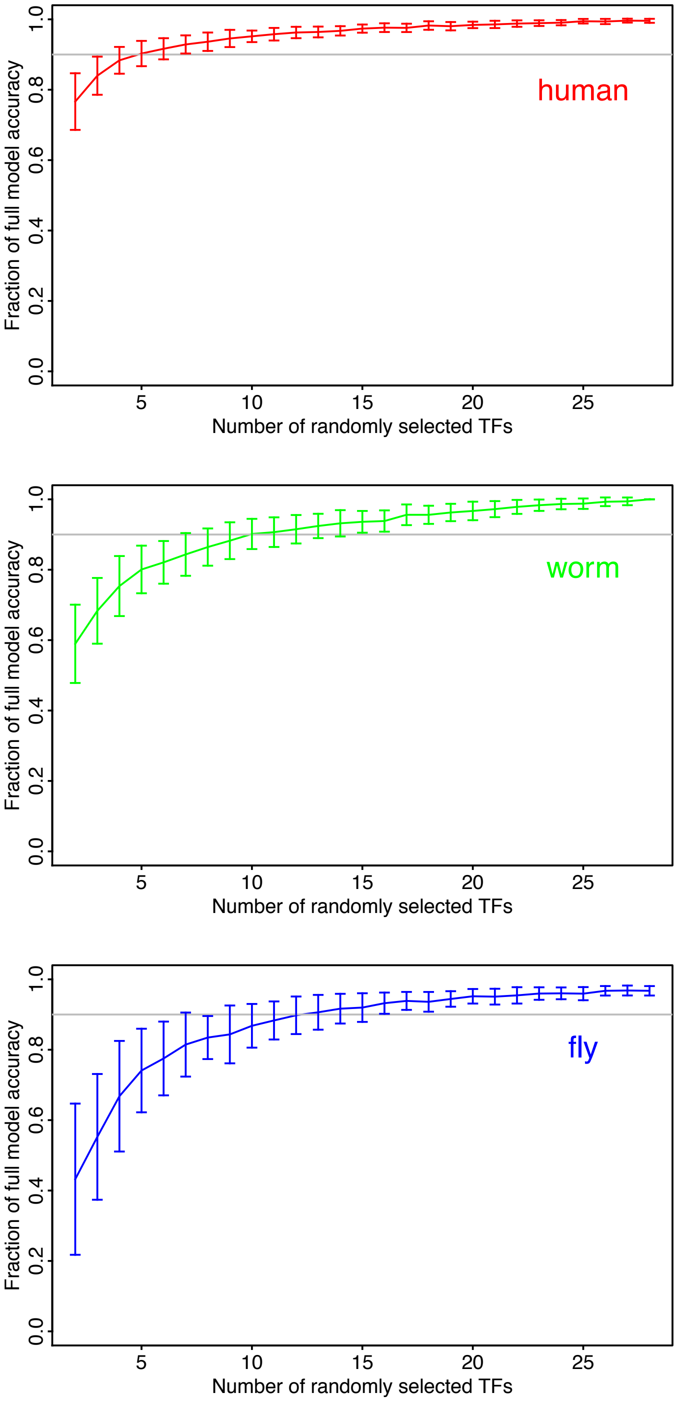

Fig. S4b - More Details on the Predictive Model for Gene Expression.

Fig. S4b1 - Average predictive accuracy of models with different number of randomly selected TFs.

We randomly selected n TFs as predictors and examined the predictive accuracy by cross-validation, with n was taken from 2 to 28. The curve shows the average predictive accuracy and the bars indicate the standard deviation of all models with the same number of predictors.

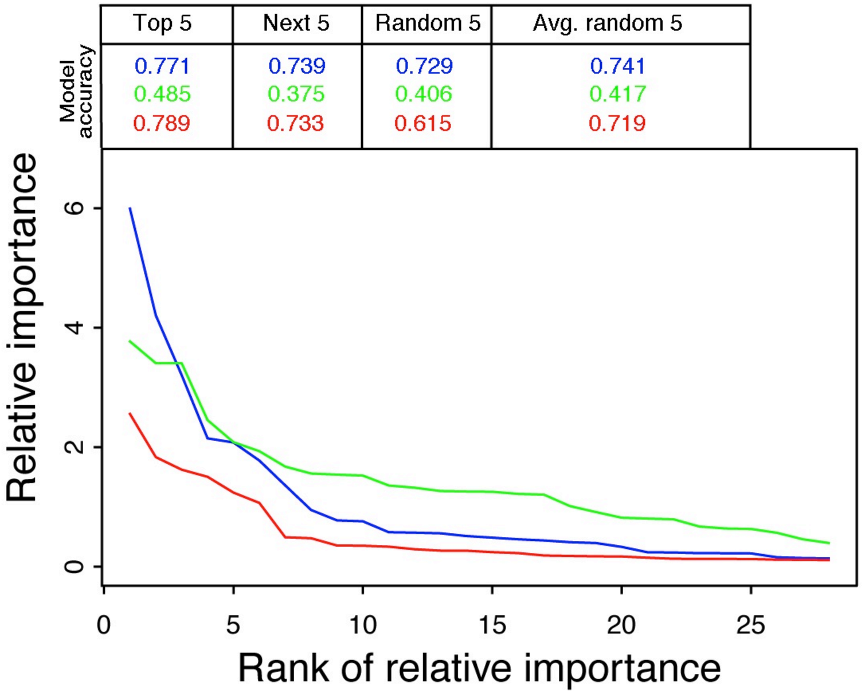

Fig. S4b2 - Relationship between TF number in a model with predictive accuracy.

TFs were ranked based on their relative importance in the full model that contains all TFs as predictors. The predictive accuracy of each model was calculated by selecting the top 5, next 5, and 5 random TFs. The average predictive accuracy of models with 5 randomly selected TFs was also calculated. The accuracy is represented as the Pearson correlation coefficient between the predicted values and the actual expression levels in the test data. (To fit the curves for all three species, we plot the models with 28 TFs.)

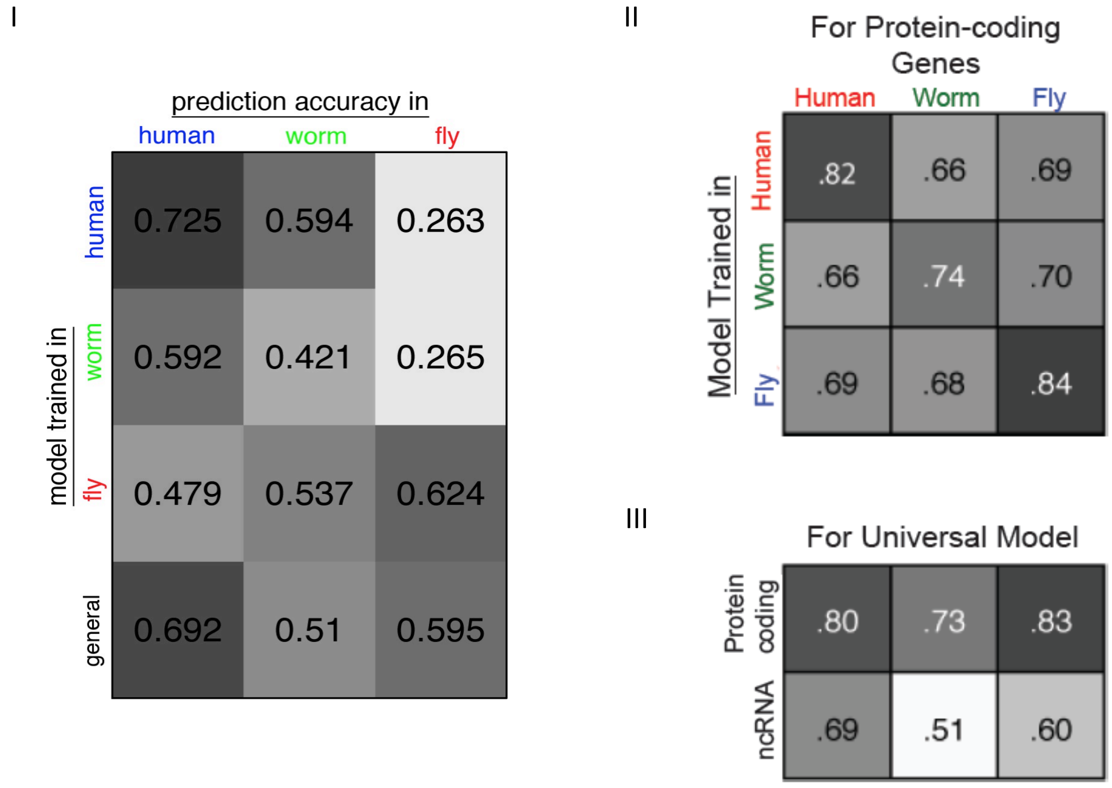

Fig. S4b3 - Application of protein-coding models to ncRNAs.

Application of histone models trained by protein-coding genes to predict expression levels of non-coding RNAs. I). In each organism, a histone model is trained on the protein-coding data and then employed to expression prediction for non-coding genes. The general (universal) model is trained on a mix of protein-coding genes with equal fraction from human, worm and fly. II and III show the performance on predicting the expression of protein-coding genes for comparison. For ncRNAs, the model performed better in human and fly but not as well in worm, perhaps reflecting less precise TSS definitions.

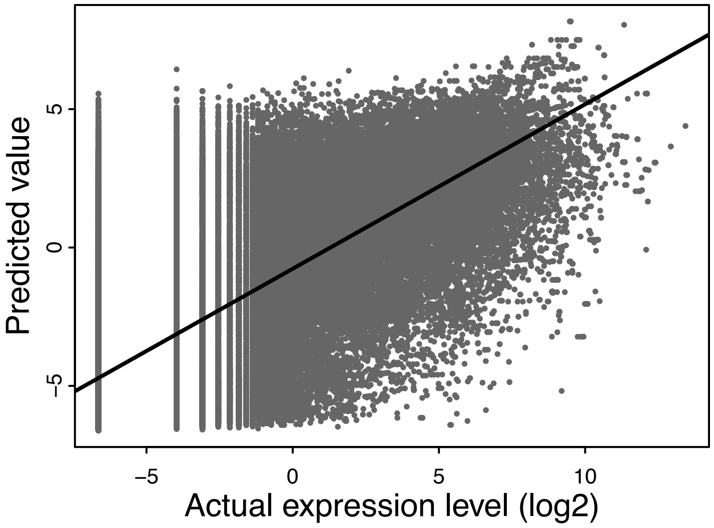

Fig. S4b4 - Comparison of predicted expression values with experimental measured levels of human protein-coding TSSs.

Histone modification signals in bin 1 (the first bin upstream of TSS of genes) are used to predict TSS expression.

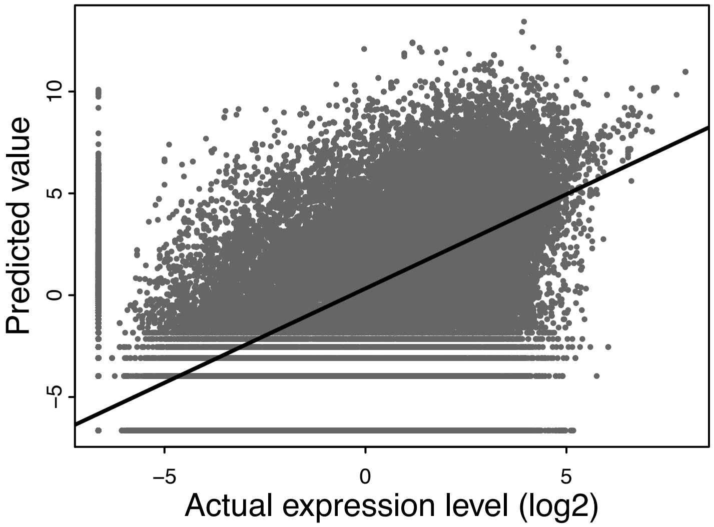

Fig. S4b5 - Predicted TSS expression values from two-step human histone model.

In our model, Random Forest classification is used to predict whether a protein-coding TSS is expressed or not. If a TSS is predicted to be not expressed, it is assigned a value of zero. Otherwise, the Random Forest regression model is then used to predict its expression value.
